# The morphology and metabolic changes of *Actinobacillus pleuropneumoniae* during its growth as a biofilm

**DOI:** 10.1186/s13567-023-01173-x

**Published:** 2023-05-26

**Authors:** Qiuhong Zhang, Lu Peng, Weiyao Han, Hongyu Chen, Hao Tang, Xiabing Chen, Paul R. Langford, Qi Huang, Rui Zhou, Lu Li

**Affiliations:** 1 National Key Laboratory of Agricultural Microbiology, College of Veterinary Medicine, Huazhong Agricultural University, Wuhan, 430070 Hubei China; 2 Key Laboratory of Preventive Veterinary Medicine in Hubei Province, The Cooperative Innovation Center for Sustainable Pig Production, Wuhan, 430070 Hubei China; 3 Institute of Animal Husbandry and Veterinary Science, Wuhan Academy of Agricultural Sciences, Wuhan, 430070 Hubei China; 4 Section of Paediatric Infectious Disease, Imperial College London, St Mary’s Campus, London, W2 1PG UK; 5 International Research Center for Animal Disease, Ministry of Science and Technology of the People’s Republic of China, Wuhan, 430070 Hubei China

**Keywords:** *Actinobacillus pleuropneumoniae*, biofilm, planktonic, morphology, transcription, metabolism, regulator

## Abstract

*Actinobacillus pleuropneumoniae* is an important swine respiratory pathogen. Previous studies have suggested that growth as a biofilm is a natural state of *A. pleuropneumoniae* infection. To understand the survival features involved in the biofilm state, the growth features, morphology and gene expression profiles of planktonic and biofilm *A. pleuropneumoniae* were compared. *A. pleuropneumoniae* in biofilms showed reduced viability but maintained the presence of extracellular polymeric substances (EPS) after late log-phase. Under the microscope, bacteria in biofilms formed dense aggregated structures that were connected by abundant EPS, with reduced condensed chromatin. By construction of Δ*pga* and Δ*dspB* mutants, polymeric β-1,6-linked N-acetylglucosamine and dispersin B were confirmed to be critical for normal biofilm formation. RNA-seq analysis indicated that, compared to their planktonic counterparts, *A. pleuropneumoniae* in biofilms had an extensively altered transcriptome. Carbohydrate metabolism, energy metabolism and translation were significantly repressed, while fermentation and genes contributing to EPS synthesis and translocation were up-regulated. The regulators Fnr (HlyX) and Fis were found to be up-regulated and their binding motifs were identified in the majority of the differentially expressed genes, suggesting their coordinated global role in regulating biofilm metabolism. By comparing the transcriptome of wild-type biofilm and Δ*pga*, the utilization of oligosaccharides, iron and sulfur and fermentation were found to be important in adhesion and aggregation during biofilm formation. Additionally, when used as inocula, biofilm bacteria showed reduced virulence in mouse, compared with planktonic grown cells. Thus, these results have identified new facets of *A. pleuropneumoniae* biofilm maintenance and regulation.

**Supplementary Information:**

The online version contains supplementary material available at 10.1186/s13567-023-01173-x.

## Introduction

Biofilms are compacted and functionally coordinated aggregates of bacterial cells attached to or embedded on biotic or abiotic surfaces, and their metabolic state, pathogenicity and immunogenicity are different from those of bacteria in the planktonic state [1, 2]. Biofilms contain extracellular polymeric substances (EPS) comprising cross-linked polysaccharides, proteins and/or extracellular DNA, which form a solid physical barrier enabling defense against external environmental stresses [3]. EPS can also mask antigenic epitopes on bacterial surfaces [4]. Bacteria in biofilms can release extracellular nucleases to degrade neutrophil extracellular traps (NETs) [5]. Such features result in biofilms inducing a lower host inflammatory response compared with their planktonic counterparts, and facilitate immune escape [6]. Bacteria inside the biofilm initiate the stringent response, enter dormancy, express efflux pumps and transfer drug-resistant genes horizontally, all of which contribute to resistance against adverse stressors [7]. Hence, the biofilm mode of growth is often associated with persistent infection and acts as a reservoir for recurrent infection.

Biofilm formation depends on the host environment and is regulated by many bacterial signal transduction systems including quorum sensing, two-component, secondary messenger transduction and stringent response systems [8–10]. In *Staphylococcus aureus*, the Agr quorum sensing (QS) system coordinates with multiple global regulatory factors including SarA, CcpA, Fur and two-component systems including SaeRS and LytSR to regulate the production of extracellular DNA, lectin and bacterial surface proteins, all of which are the main components of biofilm EPS [10, 11]. The secondary messenger molecule C-di-GMP is also a known key factor of bacteria involved in biofilm formation [12]. In *Pseudomonas aeruginosa* and *Escherichia coli*, C-di-GMP regulates the production of extracellular polysaccharides and cellulose [13].

*Actinobacillus pleuropneumoniae* is an important respiratory pathogen of swine, which has high lethality rate resulting in significant economic losses in the world swine industry [14]. In addition to acute infection, *A. pleuropneumoniae* can persist in the tonsils and lungs of sub-clinically infected pigs, becoming a potential source of disease outbreaks [15, 16]. It has been reported that most field isolates of *A. pleuropneumoniae* can form biofilms [17], and pigs naturally infected with the bacterium can be present as biofilm aggregates in the lungs [18]. A recent study found a negative correlation between the ability of field isolates of *A. pleuropneumoniae* to form biofilms in vitro and the severity of pig lung pathological injury [17]. Additionally, it has also been reported that *A. pleuropneumoniae* present in swine farm environmental samples show strong biofilm formation in vitro [19], and the bacterium can use products of other bacteria to enable its growth in mixed biofilms [20, 21]. *A. pleuropneumoniae* growing in the biofilm mode of growth has enhanced antibiotic resistance [22], and a low propensity to stimulate the immune system because of lipid A modification [23]. Therefore, growth in biofilms is considered a natural state of *A. pleuropneumoniae* during persistent infection [21]. Understanding the structure and survival mechanisms of *A. pleuropneumoniae* in biofilms is crucial for developing methods of prevention and eliminatation.

The regulation and formation of *A. pleuropneumoniae* biofilms is a complex process. Previous studies have shown that poly-beta (1,6)-N-acetyl-d-glucosamine (PNAG), synthesized and exported by proteins encoded by the *pgaABCD* locus, is a major polysaccharide component of the EPS of some Gram-negative bacteria including *A. pleuropneumoniae* [24]. *A. pleuropneumoniae* also possesses the gene encoding dispersin B (*dspB*) which is a glycosidase that degrades PNAG [25]. The histone-like nucleoid structuring protein H-NS and sigma E directly represses and activates the expression of *pga*, respectively [26]. Many other genes have been found to directly or indirectly affect *A. pleuropneumoniae* biofilm formation, including those encoding the type 2 quorum sensing signal synthetic enzyme LuxS, the two-component signaling systems (TCS) ArcAB and CpxAR, and the stringent response system [27–32]. The transcriptomes of static and biofilms at different growth stages in a drip-flow apparatus have also been compared [33]. Generally, genes involved in energy metabolism were down-regulated, while genes encoding some transporters were up-regulated in biofilms, compared with the planktonic counterparts. The study [33] used microarray hybridization, a technique that has now been superseded by RNA-seq.

To further understand the survival and regulation mechanisms of *A. pleuropneumoniae* growing as a biofilm, in this study, we compared the growth features, morphologies, gene expression profiles (using RNA-Seq) and virulence in mice of planktonic and biofilm grown bacteria. Significant differences in growth, morphology, transcriptome and virulence were found, and three major regulators (Fnr/HlyX and Fis) were identified as being important for the biofilm mode of growth. Our results deepen the understanding of the *A. pleuropneumoniae* biofilm mode of growth.

## Materials and methods

### Bacterial strains and growth conditions

The strains, plasmids and primers used in this study are listed in Additional file 3. All *A. pleuropneumoniae* strains were cultured at 37 °C on TSA plates or in TSB medium (Becton, Dickinson and Company, NJ, USA) supplemented with 10 μg/mL NAD. Chloramphenicol at 2 μg/mL was added into the medium when screening for *A. pleuropneumoniae* mutants or complementary strains containing recombinant plasmid. All *E. coli* strains were cultured at 37 °C on LA plate or in LB medium (OXOID, Hampshire, UK). *E. coli* DH5α, TOP10 and β2155 containing recombinant plasmids were screened on LA plates containing 20 μg/mL chloramphenicol. An additional 50 μg/mL 2,6-diaminoheptanoic acid (DAP, Sigma-Aldrich, Darmstadt, Germany) supplementation was required for the cultivation of strain β2155.

### Culture and detection of growth feature of planktonic bacteria

For each *A. pleuropneumoniae* culture replicate, a single colony of the bacteria on TSA plate kept in 4 °C less than 4 days was incubated into TSB medium overnight (12–14 h) by shaking at 180 r/min at 37 °C. For culture of *A. pleuropneumoniae* planktonic bacteria (PK), overnight-cultures were transferred to 5 mL fresh TSB medium at 1:100 and cultured at 37 °C at 180 r/min. Samples of planktonic bacteria were taken at 0, 2.5, 4, 5.5, 8, 12 and 24 h. At each timepoint, the OD_600nm_ of the culture was measured and bacterial numbers were determined by viable bacterial counts by plating the cultures onto TSA plates after serial dilutions in normal saline from 10^–3^ to 10^–6^. Three plating replicates were conducted for each dilution. After incubation of the plates at 37 °C overnight, the colony forming units (CFU) on each plate were recorded and the original bacterial numbers in each group were calculated.

### Culture and detection of growth feature of biofilm

For culture of biofilm (BF), overnight-cultures of *A. pleuropneumoniae* were transferred into BHI (OXOID, Hampshire, UK) [8] at 1:75 in 12-wells flat bottom cell culture plates (LabServ; Thermo Fisher Scientific, Shanghai, China) at 2 mL per well and incubated at 37 °C statically. Samples were collected at 0, 2.5, 4, 5.5, 8, 12 and 24 h for bacterial numbers measurement and crystal violet (CV) staining of biofilm. The upper suspension was separated and diluted in normal saline for determination of bacterial numbers. The adhesion layer bacteria were collected as biofilms and resuspended in 2 mL of saline, dispersed totally by vortex and diluted in saline. The bacterial numbers in the dilutions were determined by viable bacterial counts as described above.

For quantifications of the biomass of the adherent layer, the upper suspensions were discarded and the wells were gently washed twice with normal saline to remove unattached bacteria and residual medium, followed by fixation of the adherent layer after air-dry. Then, 2 mL of 0.1% Crystal Violet Ammonium Oxalate Solution (Beijing Solarbio Science & Technology Co., Ltd. China) was added into each well to stain the fixed biofilm layer, which was incubated at 37 °C for 15 min. After washing the wells with saline, the dyed adherent layers were dissolved by 2 mL of 33% glacial acetic acid solution per well and resuspended by pipetting. The OD_595nm_ of the dissolved solutions in the wells were measured.

### Construction of mutants and complementary strains

The mutants Δ*pgaABCD* and Δ*dspB* were constructed according to the methods described in our previous studies [34]. Briefly, using *A. pleuropneumoniae* 4074 genome as template, upstream and downstream fragments of *pgaABCD* and *dspB* were amplified with primers *pga*1/2-*pga*3/4 and *dspB*1/2-*dspB*3/4, respectively (Additional file 3). The amplified products were ligated to the SalI/NotI site of pEMOC2 [35] using the ClonExpress MultiS One Step Cloning Kit (Nanjing Vazyme Biotech Co., Ltd., China). Then the recombinant plasmid was transformed from *E. coli* β2155 to *A. pleuropneumoniae* WT strain by conjugation [35]. The suspected mutants were screened using the plates supplemented with 2 μg/mL chloramphenicol and 10% (v/w) sucrose and mutants confirmed by PCR using the primers in Additional file 3.

The complementary strains were constructed using the plasmid pMC-express [36] (Additional file 3). The full-length fragments of *pgaABCD* and *dspB* were amplified with primers pMC-*pga* F/R and pMC-*dspB* F/R, respectively, using *A. pleuropneumoniae* 4074 genome as template (Additional file 3). The amplification products were cloned into pMC-express vector cut with KpnI and NotI. The recombinant plasmid was transformed into the corresponding mutant strains by electroporation. The transformants were screened using the plates with chloramphenicol (2 μg/mL) and the complementary strains were confirmed by PCR using the primers in Additional file 3.

### Morphological observations

The planktonic and biofilm grown bacteria were cultured to logarithmic phase as described above and observed under a transmission electron microscope (TEM) and a scanning electron microscope (SEM). Planktonic or bacteria obtained from the biofilm adhesion layer were centrifuged at 5000 *g* for 5 min and fixed in 2.5% glutaraldehyde. For observation under TEM, the fixed cells were rinsed for 15 min in 0.1 M phosphate buffer (pH 7.4) 3 times. The cells were further fixed with 1% osmic acid-0.1 M phosphate buffer (pH 7.4) at room temperature for 2 h and rinsed with 0.1 M phosphate buffer (pH 7.2) 3 times. The samples were dehydrated by an alcohol gradient (30%, 50%, 70%, 80%, 90%, 95%, 100%) and permeated by epoxy (2:1), acetone: epoxy (1:1) and epoxy. The epoxy was embedded and sectioned, and then double-stained with uranium and the bacterial morphologies were observed under TEM (Tecnai G20 TWIN, FEI, USA).

For observation under SEM, the immobilized bacterial samples were washed with PBS (0.1 M, no NaCl) 3–5 times. After gradient dehydration with alcohol, the samples were treated with isoamyl acetate 3 times each for 20 min. The dehydrated samples were dried and sprayed in vacuum. Finally, the morphology of bacteria was observed using high-resolution SEM (Hitachi U8010, Hitachi, Ltd., Japan).

### RNA extraction and transcriptome analysis

After overnight culture, the WT planktonic bacteria were sub-cultured for 4 h to log-phase. WT and Δ*pga* were sub-cultured in 12-well plates at 37 °C for 5 h (log-phase). WT biofilms were sampled from the adherent layer. Since *Δpga* does not form any adherent layer, the whole cultures of this strain were sampled. Then, the planktonic and biofilm grown cells were collected by centrifugation at 5000 *g*, and total RNA extracted using the Total RNA Extraction Kit (Tianmo Biotech, Beijing, China) according to the protocol recommended by the manufacturer. Each group of samples had three independent biological replicates.

After rRNA removal and quality control, the RNA samples were sequenced. Paired-end sequencing was performed on the Illumina sequencing platform and data quality assessment and filtering was performed by FASTQC and Trimmomatic software. The high-quality sequences obtained after quality control were mapped to the *A. pleuropneumoniae* 4074 genome (NZ_CP029003.1) based on the Burrows-Wheeler method. The RPKM (Reads per Kilobase per Million Reads) value was used as the index of gene expression. The genes with fold change ≥ 2 and *p* adjust ≤ 0.05 were regarded as differentially expressed genes. Hierarchical clustering was used to classify genes with different expression regulation patterns. Pathway analysis was performed by KEGG pathway enrichment of differentially expressed genes (*p* value ≤ 0.05).

### Quantitative real-time RT-PCR

Culture and RNA extraction of bacteria were described above. Residual gDNA was digested and cDNA was synthesized using the HiScript II 1st Strand cDNA Synthesis Kit (+ gDNA wiper; Nanjing Vazyme Biotech Co., Ltd., China). Using the obtained cDNA as template, qPCR was performed with TB Green Premix Ex Taq II Kit (Tli RNaseH Plus) (Takara Biomedical Technology (Beijing) Co., Ltd., China) using primers of differentially expressed genes listed in Additional file 3. The 2^−ΔΔCt^ of each gene was calculated as the relative expression level with the 16S rRNA expression amount as the endogenous reference.

### Mouse infection assay

A model of intranasal infection in mice was used to compare the virulence of *A. pleuropneumoniae* planktonic with biofilm bacteria as described in a previous study [34]. PK and BF bacterial solutions for infection were prepared from log-phase culture as described above. Four-week-old female Kunming mice were anesthetized with 20 μL/g of Avertin (1.25% tribromoethanol) and intranasally infected with 20 μL inoculum containing 4 × 10^6^ CFU biofilm (*n* = 6) or the same dose of planktonic bacteria (*n* = 6), and number of mice surviving recorded for 72 h. For detection of the bacterial loads in the lungs after sub-lethal dose infection of biofilm (*n* = 6) or planktonic bacteria (*n* = 6), mice were infected intranasally with bacterial cultures from mid-log phase at a dose of 2 × 10^6^ CFU. Then, mice were euthanized at 12, 48 and 72 h after infection. The lungs were collected and thoroughly homogenized and plated on TSA plates after dilution, and the CFUs determined.

### Sequence analysis of potential binding sites of regulators

For binding site analysis of differential gene promoter regions, the reference binding motifs of Fnr and Fis were extracted from DB database. The MEME-FIMO online tool (Find Individual Motif Occurences, *p*-value < 0.001) was used to search the promoter regions of selected genes for the specific binding motifs of Fnr, Fis and H-NS.

### Statistical analysis

A two-tailed t-test was used for statistical analysis of biofilm quantification. The log rank (Mantel-Cox) test was performed for comparison of mortalities of mice in the infection assay. Bacterial loads in the lungs of mice were statistically analyzed using the Mann–Whitney test. Differences with *p* < 0.05 were considered to be significant. For RNA-seq and pathway analysis, the statistical methods have been described above.

## Results

### *A. pleuropneumoniae* biofilm differs from planktonic bacteria in their growth curves

**Figure 1:**
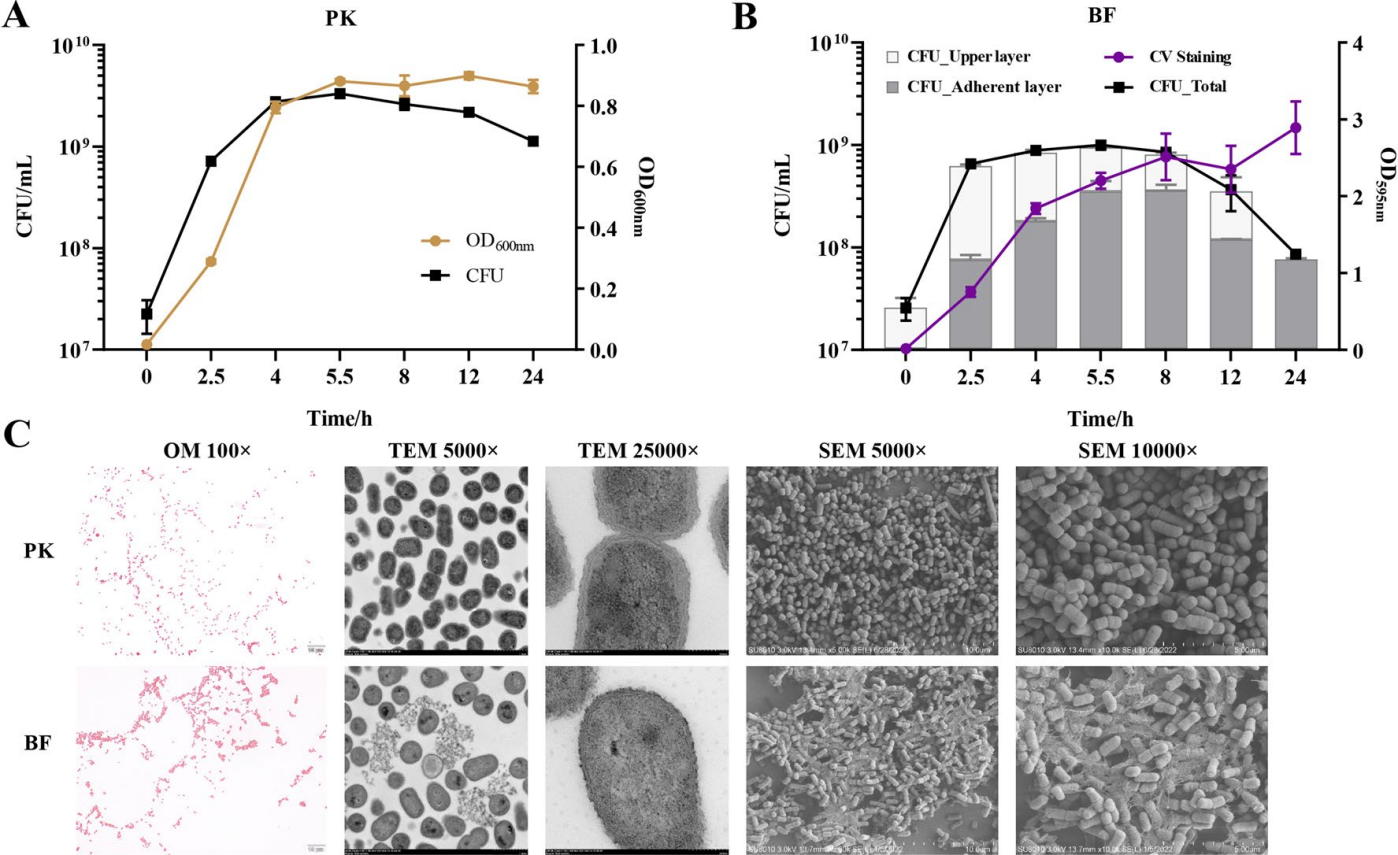
**Growth curve and morphological structures of** ***A. pleuropneumoniae*** **biofilm and planktonic bacteria.** **A** Growth curves represented by bacterial culture absorbance at OD_600nm_ and colony forming units (CFU) of *A. pleuropneumoniae* planktonic bacteria (PK) with shaking at 37 °C. **B** Growth curves of biofilm cells (BF) shown by CFU of the upper and adhesion layers of the biofilm cultures grown statically in 12-well plates (bar graphs). The total CFU of both upper and adhesion layers of the biofilm is shown (black line). Biomass of the biofilms were determined by crystal violet (CV) staining of the adhesion layer (purple line). **C** Morphology of PK and BF grown cells under an optical microscope (OM; Gram staining), transmission electron microscope (TEM) and scanning electron microscope (SEM). The magnification of the image is shown. For growth curves and CV staining of biofilms, data are shown as means ± SD from three independent replicates.

*A. pleuropneumoniae* planktonic or biofilm growth were determined by detecting OD_600nm_ and colony-forming units (CFU) (Figures 1A, B). Bacteria growing in shaking culture or statically in microtiter plates were the source of planktonic and biofilm cells, respectively. For biofilm grown bacteria, CFUs of adherent and non-adherent (upper layer) were determined. Biomass was also quantified by crystal violet (CV) staining. In the early stage of culture (5–6 h), the growth trends of planktonic bacteria and biofilm were similar (Figures 1A, B). Thereafter, as the biofilm approached maturity, planktonic bacteria entered the stationary phase with the highest CFU (Figures 1A, B). The biomass detected by CV staining increased in the early stage of biofilm formation (2.5–5.5 h), which was consistent with the trend of bacterial proliferation as detected by the CFU in both upper and adherent layers (Figure 1B). As the biofilm matured, the quantities of viable bacterial numbers reached its peak at 5.5–8 h, corresponding with the thickest EPS of the biofilm according to the CV staining (Figure 1B). At the later stage of biofilm formation, the quantities of the biomass were stable until 24 h, but the number of viable bacteria in the biofilm layer and upper layer decreased (Figure 1B). Taken together, under the conditions used in this study, the biofilm growth of *A. pleuropneumoniae* entered maturation at 5–5.5 h, the same time as that of late-log phase and the beginning of the stationary phase of planktonic bacteria. Thereafter, the biomass of biofilm was maintained to 24 h, but the number of viable bacteria declined, while the viable bacteria in planktonic cultures was maintained to 24 h.

### Morphological structure of *A. pleuropneumoniae* biofilms

The morphologies of biofilms in the mature stage (5 h) were observed. Gram staining showed that, compared with planktonic bacteria, those grown as biofilm aggregated into clusters with flocculent impurities that could be stained around the clusters (Figure 1C). Transmission electron microscopy revealed that, compared to planktonic bacteria, there were numerous interconnected extracellular flocs in biofilm grown bacteria (Figure 1C), inside of which condensed chromatin could be seen as black clumps or filaments. Compared with planktonic bacteria, there were fewer black masses inside the biofilm (Figure 1C). In mature biofilms observed under scanning electron microscopy, there were dense aggregated structures connected by abundant EPS (Figure 1C).

### PNAG and dispersin B are critical for *A. pleuropneumoniae* normal biofilm formation

**Figure 2:**
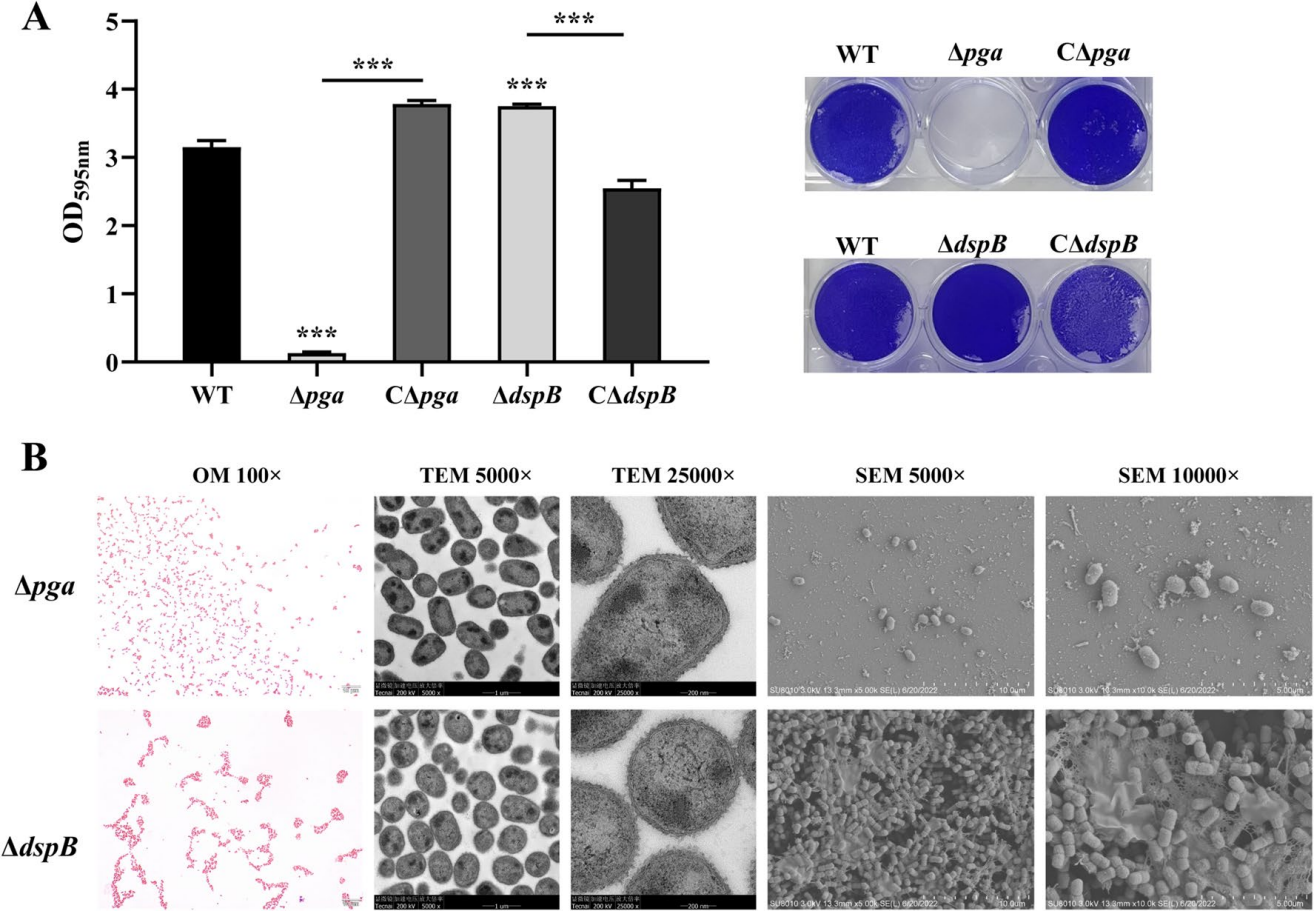
**Morphology of Δ*pga* and Δ*dspB.*** **A** CV staining of biofilms formed by WT, Δ*pga* and Δ*dspB* cultured for 5 h in 12-well plates. Data are shown as means ± SD from three independent replicates. A two-tailed t-test was used to compare the biofilm quantities of WT and the Δ*pga* or Δ*dspB* (***, *p* < 0.001). **B** Morphology of Δ*pga* and Δ*dspB* under optical microscopy (OM; Gram staining), transmission electron microscope (TEM) and scanning electron microscope (SEM). The magnifications of the images are shown

It has been reported that PNAG and dispensin B are essential for the formation and degradation of *A. pleuropneumoniae* biofilm extracellular matrix [25]. To verify the role of PNAG in biofilm formation and the role of dispersin B in biofilm dispersion of *A. pleuropneumoniae*, we constructed the deletion mutants and complementary strains of *pgaABCD* and *dspB*, respectively (Additional file 1). Deletion of *pgaABCD* (Δ*pga*) and *dspB* (Δ*dspB*) did not affect *A. pleuropneumoniae* growth (Additional file 2). Gram-staining and electron microscopy indicated that Δ*pga* failed to adhere to microplate surfaces/slide to form a biofilm structure (Figure 2). The results confirmed that PNAG was essential for the formation of a biofilm. In contrast, *ΔdspB* exhibited obvious enhanced biofilm formation, with more aggregates and extracellular matrix (Figure 2). Therefore, the above results confirmed that *A. pleuropneumoniae* formed biofilm dependent on PNAG as the main component of the EPS, while dispersin B was involved in limiting biofilm formation.

### *A. pleuropneumoniae* grown in biofilms have significantly altered gene transcriptional patterns from planktonic bacteria

**Figure 3:**
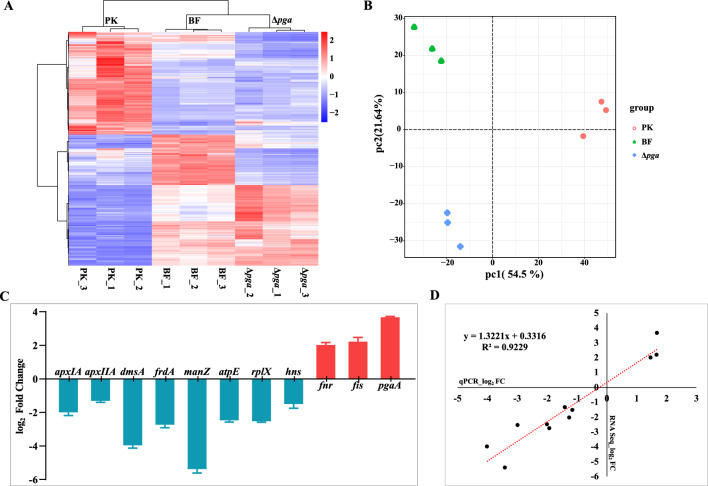
**Transcriptome levels and gene expression difference analysis of PK, BF and Δ*pga.*** **A** Heatmap of differentially expressed genes from all comparison groups (PK, BF and Δ*pga*) after RNA-seq analysis. The horizontal line represents genes, while each column represents a sample. Each treatment group consisted of three independent biological replicates. Red: genes with increased expression levels. Blue: genes with decreased expression levels. **B** Principal component analysis of gene expressions from all comparison groups (PK, BF and Δ*pga*) after RNA-seq analysis. Different colors represent different groups. **C** The log_2_ fold change values of qRT-PCR showing the transcriptional changes of 11 differentially expressed genes in BF compared with PK group from RNA-seq analysis. **D** Pearson coefficient correlation analysis between qRT-PCR and RNA-seq analysis. X-axis: log_2_ value from qRT-PCR; Y-axis: log_2_ value from RNA-seq.

Planktonic wild-type (WT) bacteria were grown to mid-log phase, and WT and Δ*pga* were also grown statically in microplates until the biofilm maturity stage (5 h). The biofilm/adhesion layer of WT (BF), *Δpga* (unable to generate adhesion layer), and planktonic WT (PK) were harvested for RNA sequencing and analysis. According to the hierarchical clustering heatmap (Figure 3A) and principal component analysis (Figure 3B), there was good reproducibility among the three independent samples in each group. The PK and BF groups had different hierarchical clustering patterns (Figure 3A) and great distance on the x-axis (Figure 3B), indicating that the transcriptional patterns of bacteria grown in biofilms were significantly different to those grown planktonically. Compared with the PK group, there were 646 differentially expressed genes (332 up-regulated, 314 down-regulated) in the BF group (Additional file 4). Generally, the transcriptional pattern of the Δ*pga* group was more similar with that of BF group (Figures 3A, B, Additional file 5), with fewer differentially expressed genes (198 up-regulated, 15 down-regulated). Transcript levels of 11 genes were determined by qRT-PCR under the same sampling conditions as RNA-Seq (Figure 3C) and correlated well with transcriptome data (Figure 3D).

### The transcriptional levels of genes in multiple metabolic pathways of *A. pleuropneumoniae* grown in biofilms were significantly changed

**Figure 4:**
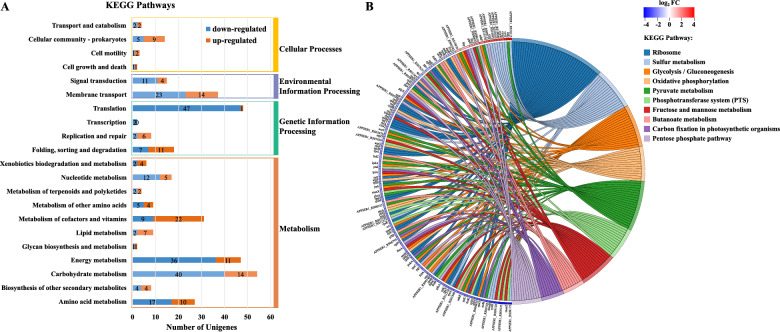
**Functional classification and pathway enrichment analysis of differentially expressed genes in BF compared with PK bacteria.** **A** Statistical histogram showing pathway classification of the distribution of differentially expressed genes in BF compared with PK group at different KEGG pathway levels. **B** KEGG pathway enrichment chord diagram showing differentially expressed genes in BF compared with PK group corresponding to significantly enriched pathways (*p* value ≤ 0.05). The left half circle is the differentially expressed genes arranged according to the value of log_2_FC from large to small. The right half circle is the KEGG pathways of the significantly enriched differentially expressed genes

**Figure 5:**
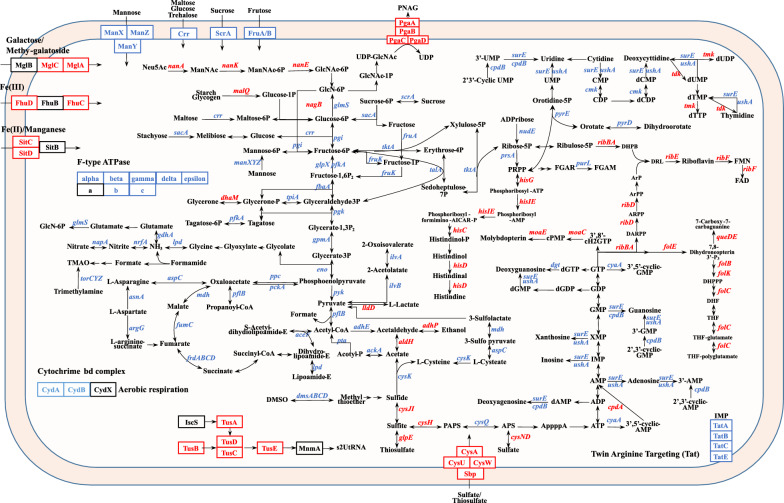
**Schematic diagram of metabolic pathway changes of BF compared with PK bacteria.** Red: up-regulated genes; Blue: down-regulated genes.

KEGG pathway enrichment analysis was performed on genes differentially expressed in the BF group compared with the PK group, and 124 up-regulated and 201 down-regulated genes were matched into multiple pathways (Figure 4). Among them, the genes involved in metabolism, genetic information and environmental information processing pathways accounted for 45.5%, 23.4% and 16.0% of the total, respectively. Genes involved in carbohydrate metabolism (19.9%), energy metabolism (17.9%), translation (23.4%) and membrane transport (11.4%) accounted for 72.6% (146/201) of the down-regulated, but only 32.3% (40/124) of the up-regulated genes (Figure 4A). Significantly down-regulated pathways that were enriched included oxidative phosphorylation, phosphotransferase system (PTS), glycolysis/gluconeogenesis, pyruvate metabolism, citrate cycle (TCA cycle), and ribosome synthesis (Figures 4B, 5). ATP synthase and pathways involved in aerobic respiration were significantly down-regulated. Similarly, reduction pathways such as nitrate, TMAO\DMSO and fumaric acid reductions involved in the electron transport chain of anaerobic respiration were also significantly down-regulated (Figure 5). In contrast, the fermentation pathways for lactic acid and ethanol production were up-regulated (Figure 5). The riboflavin metabolic pathway, with its product flavin mononucleotide (FMN) as a cofactor of L-lactate dehydrogenase LldD and various enzymes [37], was up-regulated (Figure 5). The catabolism of stored carbon sources, starch glycogen and N-acetylneuraminate (Neu5Ac), were up-regulated. Notably, three enzymes (Nan A/K/E) leading to the production of GlcNAc-6P, the precursor for synthesis of PNAG, peptidoglycan and lipid A [38–40], were up-regulated. As expected, the *pgaABCD* operon encoding the PNAG synthetase and export proteins was significantly up-regulated (Figure 5). In addition, up-regulated genes involved in other pathways were enriched, for example many genes involved in sulfur metabolism, which is critical for providing sulfur atoms for methionine, glutathione and iron-sulfur clusters (Figure 5). Molybdenum cofactor and folate synthesis, histidine metabolism and some transporters of Fe (III) and Fe (II) were also up-regulated. Altogether, the transcriptional changes of many metabolic genes indicated that bacteria grown in biofilms significantly reduced energy metabolism and genetic activities, increased fermentation and related cofactor synthesis, and accumulated extracellular matrix PNAG.

### Analysis of transcriptional regulation in biofilm grown *A. pleuropneumoniae*

As previously described, the formation of a bacterial biofilm and its maintenance requires signal transduction systems and regulators in response to specific conditions or stimuli. We next focused on the differentially expressed transcriptional regulators in *A. pleuropneumoniae* grown in biofilms. In particular, the global regulatory proteins Fnr (also named as HlyX) which is important for the transition from aerobic to anaerobic growth, the nucleoid associated protein Fis, and the histone-like nucleoid associated protein H-NS, were differentially expressed in the biofilm transcriptome.

Fnr is a known global regulator in response to oxygen levels [41]. It was up-regulated in *A. pleuropneumoniae* grown as a biofilm (log_2_FC = 1.470, Figure 3). Fnr of *A. pleuropneumoniae* consists of 255 amino acids, which is homologous to Fnr of *E. coli* with 75% sequence identity and also with a similar function [42]. The DNA binding motif of Fnr in the upstream region of differentially expressed genes or operons of BF were searched (Additional file 4). The results showed that 63% of these genes had Fnr binding site(s) (Table 1 and Additional file 4, *p* < 0.001). Among these genes, most of which were down-regulated, were those involved in transcription and translation, glycolysis, the TCA cycle and anaerobic respiratory electron transport chain. The up-regulated genes mainly participated in L-lactate fermentation, cofactor synthesis (FMN, molybdopterin), amino acid metabolism, Neu5Ac metabolism, PNAG synthesis, and *fnr* itself. Notably, Fnr binding sites were also found in the promoter regions of *apxICABD* and *apxIICA* encoding the important virulence factors of *A. pleuropneumoniae*, the RTX toxins [43]. These genes were also significantly down-regulated when *A. pleuropneumoniae* was growing as a biofilm.

**Table 1:** **Identification of Fnr binding sites**^**a**^** in the promoter regions of differentially expressed genes (BF compared with PK)**

Functional class	Target genes^b^	Transcription change^c^
Transcription, Translation	***rplKA****, ****rplJL****, ****rpsJ- rplCDWB- rpsS- rplV-rpsC-rplP- rpmC-rpsQ, rplNXE-rpsNH-rplFR-rpsE-rpmD-rplO-secY-rpmJ****, rpsMKD-rpoA-rplQ, ****rplU-rpmA****, ****raiA****, ****rraA***	Down
	***argS****, folE-truA, ttcA, ****rpoE***	Up
Energy metabolism	***cydAB****, ****ppc****, ****pta-ackA****, atpEFHAGDC, fbaA, ****glpX****, gpmA, ****torY****, torA, ****dmsABCD****, ****nrfABCD****, ****ppa****,**** pfkA****, ****napFDA****, ****nqrABCDEF****, ****hypBDE****, ****hypF****, ****eno, nudE-cysQ***	Down
	***glpE-ybbN, can, cysJL***	Up
Carbohydrate metabolism	***pgk, pyk, tkt, frdABCD, tpiA, pckA, ilvB, mdh,**** gloA,**** hxpB, tal,**** fumC, focA-pflB, ****lpd-aceF***	Down
	***fbp, APPSER1_RS05435, indK, lldD, gntR, APPSER1_RS09580-nanEKA-nagB***	Up
Amino acids	*metE, asnA, pepA, ****adhE, ilvE, cysK, proB, dapA, gdhA, argG, aspC, mmsB***	Down
	***gshAB, tyrA, hisC, hisIE,**** hisG, metJ*	Up
Membrane transport	*APPSER1_RS06785, ****scrA, afuA, fruBKA, ptsH, ptsI-crr***	Down
	***sbp-cysUWA, fhuCD, znuC,**** malK-lamB-malM,**** sitCD***	Up
Signal transduction	***hybOAB, sodA, cydAB, ducB, eno, pfkA, frdA, hfq***	Down
	***fnr, fbp,**** APPSER1_RS05435,**** htpG***	Up
Cofactors and Vitamins	*fabB, ****pdxST, pntAB***	Down
	***folB, ribF, ribDEBA, hemA, folE-truA, folC, lipA, cysGHDN***	Up
Lipid metabolism	*fabB, ****adhE***	Down
	***adhP, glpQ, psd***	Up
Folding, sorting and degradation	***hfq, eno, pfkA***	Down
	*moaCDE, ****rppH, pcnB-folK, tusE, htpG, tusBCD***	Up
Cellular community	***hfq****, cyaA*	Down
	***fis****, pgaABCD, ****ribDEBA, APPSER1_RS00145***	Up
Replication and repair	***dnaE***	Down
	***rnhA, nfo,**** ung*	Up
Nucleotide metabolism	***ushA, cpdB,**** pyrE, cyaA*	Down
	*dtd, ****rsml***	Up
Virulence factor	*apxICA, ****apxIICA***	Down

Genes in bold indicate those also with Fis binding sites in the promoter regions.

^a^The Fnr motif used for searching was TTGATNWNDMKCAH.

^b^Target genes were predicted from the MEME-FIMO online tool, *p*-value < 0.001.

^c^Transcriptional changes of target genes were derived from differentially expressed genes in RNA-Seq data, Down, down-regulated, log_2_FC ≤ -1, FDR < 0.05; Up, up-regulated, log_2_FC ≥ 1, FDR < 0.05.

As a multifunctional protein involved in regulation of transcription, replication and recombination, Fis is considered to be an important regulator controlling the transition from log phase to stationary phase during growth [44, 45] and was significantly up-regulated in *A. pleuropneumoniae* biofilms (log_2_FC = 1.672, Figure 3). Fis of *A. pleuropneumoniae* had 71% sequence identity with that of *E. coli*, with a conserved DNA binding domain at the C-terminus. The Fis binding motif was found in the upstream sequences of 68% of the differentially expressed genes of the BF group (Table 2 and Additional file 4, *p* < 0.001). The down-regulated genes with a Fis binding motif in the upstream region were involved in transcription and translation, glycolysis, the TCA cycle, multiple terminal respiratory enzymes and also included *apxIICA*. The up-regulated genes were involved in fermentation, FMN metabolism and folate biosynthesis. Among these genes potentially regulated by Fis, 77.5% contained Fnr binding motifs in their promoters, indicating that these genes were cross-regulated by Fnr and Fis in *A. pleuropneumoniae* biofilms (Table 2). In addition, Fis and HlyX were found to have binding sites of each other in their promoter regions (Table 2).

**Table 2:** **Identification of Fis binding sites**^**a**^** in the promoter regions of differentially expressed genes (BF compared with PK)**

Functional class	Target genes^b^	Transcription change^c^
Transcription, Translation	***rplNXE-rpsNH-rplFR-rpsE-rpmD-rplO-secY-rpmJ****, rpmBG, ****rplU-rpmA****, ****rplKA****, rpsP-rimM-trmD-rplS, rpsT, rpsB-tsf, rpsO, rplM-rpsI, ****rpsJ-rplCDWB- rpsS- rplV-rpsC-rplP- rpmC-rpsQ****, rpsF-priB-rpsR-rplI, rpsL, ****rplJL****, rpoZ, frr, ****raiA****, ****rraA***	Down
	***argS****, ****rpoE,**** rimP-nusA-infB*	Up
Energy metabolism	***cydAB****, ****ppc****, ****pta-ackA****, ****dmsABCD****, ****napFDA****, ****nrfABCD****, ****ppa****, ****pfkA****,**** torY****, ****glpX****,**** eno****, ****hypBDE****, ****hypF****, ****nqrABCDEF****, ****nudE-cysQ***	Down
	***glpE-ybbN****, ****can****, ****cysJL****, fdhE*	Up
Carbohydrate metabolism	***pgk, tkt, frdABCD, tpiA, pckA, ilvB, mdh, hxpB, tal,**** manXYZ, fucO, ****lpd-aceF***	Down
	***fbp, APPSER1_RS05435, indK, lldD, APPSER1_RS09580-nanEKA-nagB, gntR***	Up
Amino acids	*glmS, ****adhE, ilvE, cysK, proB, dapA, gdhA, argG, aspC, mmsB***	Down
	***gshAB, tyrA, hisC, hisIE,**** hisD, APPSER1_RS10930*	Up
Membrane transport	***scrA, afuA, fruBKA, ptsH, ptsI-crr,**** xylGH, cbiKLM, ompW*	Down
	***sbp-cysUWA, fhuCD, znuC,**** fliY, ****sitCD***	Up
Signal transduction	***hybOAB, sodA, cydAB, ducB, eno, pfkA, frdA, hfq***	Down
	***fnr, fbp, htpG***	Up
Cofactors and Vitamins	*ushA, ****pdxST, pntAB****, ilvB, ilvE*	Down
	***folB, ribF, ribDEBA, hemA, folE-truA, folC, lipA, cysGHDN***	Up
Lipid metabolism	***adhE***	Down
	***adhP, glpQ, psd***	Up
Folding, sorting and degradation	***hfq, eno, pfkA,**** secG*	Down
	***rppH, pcnB-folK, tusE, htpG, tusBCD***	Up
Cellular community	***hfq,**** secG*	Down
	***fis, ribDEBA,**** dksA,**** APPSER1_RS00145***	Up
Replication and repair	***dnaE***	Down
	*holD-rimI-srmB-pdxH, dnaG, ****rnhA, nfo***	Up
Nucleotide metabolism	***ushA, cpdB***	Down
	*trmA, ****rsml***	Up
Virulence factor	***apxIICA***	Down

Genes in bold indicate those also with Fnr binding sites in the promoter regions.

^a^The Fis motif used for searching was GNNBRWWWWWTVNNCRN.

^b^Target genes were predicted from the MEME-FIMO online tool, *p*-value < 0.001.

^c^Transcriptional changes of target genes were derived from differentially expressed genes in RNA-Seq data, Down, down-regulated, log_2_FC ≤ -1, FDR < 0.05; Up, up-regulated, log_2_FC ≥ 1, FDR < 0.05.

The known *A. pleuropneumoniae* biofilm regulatory protein H-NS [26] was down-regulated in the biofilm transcriptome (log_2_FC = − 1.158, Figure 3). H-NS binding sites were found in the upstream regions of 20.7% differentially expressed genes of the BF group (Table 3 and Additional file 4, *p* < 0.001), i.e., less than that with Fnr and Fis binding motifs.

**Table 3:** **Identification of H-NS binding sites**^**a**^** in the promoter regions of differentially expressed genes (BF compared with PK)**

Functional class	Target genes^b^	Transcription change^c^
Transcription, Translation	*rpsP-rimM-trmD-rplS, psG-fusA-tuf, rpsT, rpsL*	Down
	*rimP-nusA-infB*	Up
Energy metabolism	*ppc, pta-ackA, nrfABCD, atpEFHAGDC, glpX, fumC*	Down
	*fdhE, fdhC, can*	Up
Carbohydrate metabolism	*tpiA, mdh, tal*	Down
	*lld*	Up
Amino acids	*mmsB, cysK, metE, ackA*	Down
	*marC, filY*	Up
Membrane transport	*afuA*	Down
	*fhuCD, fliY, sitCD*	Up
Signal transduction	*hfq, htpG*	Up
Cofactors and Vitamins	*fabB, pntAB*	Down
	*folB, ribF, ribDEBA, hemA, folC, lipA*	Up
Lipid metabolism	*fabB*	Down
	*glpQ*	Up
Folding, sorting and degradation	*hfq, secG*	Down
	*rppH, htpG*	Up
Cellular community	*cyaA, secG, hfq*	Down
	*fis, cpdA, APPSER1_RS00145*	Up
Replication and repair	*rnhA, nfo*	Up
Nucleotide metabolism	*ushA, cpdB, pyrE, purL-APPSER1_RS09210, cyaA*	Down
	*cpdA, rsml*	Up

^a^The H-NS motif used for searching was RATAWH.

^b^Target genes were predicted from the MEME-FIMO online tool, *p*-value < 0.001.

^c^Transcriptional changes of target genes were derived from differentially expressed genes in RNA-Seq data, Down, down-regulated, log_2_FC ≤ -1, FDR < 0.05; Up, up-regulated, log_2_FC ≥ 1, FDR < 0.05.

### Genes associated with bacterial aggregation and adhesion during biofilm formation

Using the transcriptome of Δ*pga* which cannot adhere and form biofilms as the control, the genes differentially expressed in BF were considered as being associated with bacterial aggregation and adhesion during biofilm formation. Among these genes, 198 were up-regulated and 15 were down-regulated, with more than 64% of the genes involved in metabolism. The up-regulated genes included those taking part in metabolism and transport of galactose, mannose, maltose, fucose and Neu5Ac (Table 4 and Additional file 5). These oligosaccharides usually form or modify cell membrane glycoconjugates such as glycoproteins and lipopolysaccharides, which are involved in cell structure, bacterial recognition and adhesion. Four enzymes involved in fermentation were all up-regulated in biofilms, as were those involved in the utilization of iron and sulfur sources. Among the 15 down-regulated genes, 11 encoded multiple terminal respiratory enzymes (the nitrate/nitrite reductase, TMAO reductase, DMSO reductase and hydrogenase). Therefore, the utilization of oligosaccharides, iron and sulfur, fermentation and repressed anaerobic respiration may be involved in mediating the adhesion or aggregation of bacteria during *A. pleuropneumoniae* biofilm formation.

**Table 4:** **The differentially expressed genes of BF compared with Δ*pga***

Function	Gene_ID	Gene_Name	Description
Up-regulated genes/operons (log_2_FC ≥ 1, FDR < 0.05)			
Carbohydrate metabolism	APPSER1_RS05460-APPSER1_RS05465	*galT/K*	galactokinase
	APPSER1_RS07965-APPSER1_RS07970	*mglAC*	galactose/methyl galactoside ABC transporter
	APPSER1_RS09115-APPSER1_RS09120	*manYZ*	mannose PTS system
	APPSER1_RS09135	*idnK*	gluconokinase
	APPSER1_RS06715-APPSER1_RS06725	*malM-*APPSER1_RS06720-*malK*	maltose/maltodextrin ABC transporter
	APPSER1_RS06730, APPSER1_RS06740	*malE-malG*	maltose/maltodextrin ABC transporter
	APPSER1_RS05415	APPSER1_RS05415	glycosyltransferase
	APPSER1_RS09220	*fucRIKU*	L-fucose isomerase; L-fuculokinase; L-fucose mutarotase
	APPSER1_RS09580-APPSER1_RS09605	APPSER1_RS09580-*nanEKA-nagBA*	N-acetylmannosamine kinase; N-acetylneuraminate lyase; glucosamine-6-phosphate deaminase; N-acetylglucosamine-6-phosphate deacetylase
Fermentation	APPSER1_RS02385-APPSER1_RS02390	*lldEF*	L-lactate dehydrogenase
	APPSER1_RS02395	*lctP*	L-lactate permease
	APPSER1_RS10140	*lldD*	FMN-dependent L-lactate dehydrogenase LldD
	APPSER1_RS10665	*adhP*	alcohol dehydrogenase AdhP
Transporter	APPSER1_RS07470-APPSER1_RS07490	*ccmEDCBA*	cytochrome c maturation protein; heme exporter protein
	APPSER1_RS04515-APPSER1_RS04525	APPSER1_RS04515-APPSER1_RS04525	dipeptide/oligopeptide/nickel ABC transporter
	APPSER1_RS09260-APPSER1_RS09265	APPSER1_RS09260-APPSER1_RS09265	amino acid ABC transporter
	APPSER1_RS09375	*ulaAB*	PTS ascorbate transporter
	APPSER1_RS07040-APPSER1_RS07045	*mlaEF*	phospholipid ABC transporter
	APPSER1_RS10020	*glpT*	glycerol-3-phosphate transporter
	APPSER1_RS10940-APPSER1_RS10945	*fhuCD*	iron-siderophore ABC transporter
	APPSER1_RS10115-APPSER1_RS10130	*sbp-cysTW-cysA*	sulfate ABC transporter
Reductase	APPSER1_RS10085-APPSER1_RS10090	*cysJI*	assimilatory sulfite reductase; assimilatory sulfite reductase
	APPSER1_RS10095-APPSER1_RS10110	*cysGHDN*	phosphoadenylyl-sulfate reductase
Others	APPSER1_RS10595	*cpdA*	3',5'-cyclic-AMP phosphodiesterase
	APPSER1_RS02045-APPSER1_RS02050	*ribDE*	uracil reductase RibD; riboflavin synthase
	APPSER1_RS05435	*katE*	catalase
	APPSER1_RS10970	*hisG*, *hisD*,* hisC*	ATP phosphoribosyltransferase; histidinol dehydrogenase; histidinol-phosphate transaminase
Down-regulated genes/operons (log_2_FC ≤ -1, FDR < 0.05)			
Reductase	APPSER1_RS00525-APPSER1_RS00530	*nrfAB*	nitrite reductase
	APPSER1_RS07905-APPSER1_RS07915	*napFDA*	nitrate reductase
	APPSER1_RS09175-APPSER1_RS09190	*dmsABCD*	anaerobic dimethyl sulfoxide reductase
	APPSER1_RS03675	*torY*	trimethylamine-N-oxide reductase
	APPSER1_RS07240	*hyaA*	hydrogenase 2

### Biofilm-grown *A. pleuropneumoniae* showed reduced virulence in mice

**Figure 6:**
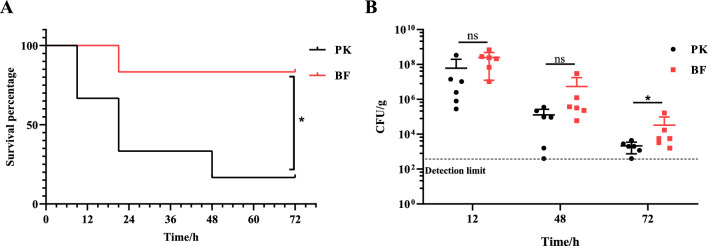
**Pathogenicity of** ***A. pleuropneumoniae*** **biofilm (BF) and planktonic (PK) grown bacteria for mice.** **A** Survival rates of mice (6 per group) intranasally infected with PK and BF, respectively, at 4 × 10^6^ CFU in 20 μL for each mouse. Log rank (Mantel-Cox) test was performed for statistical analysis (*, *p* < 0.05). **B** Bacterial loads in the lungs of mice intranasally infected with PK or BF at a dose of 2 × 10^6^ CFU per mouse. Dotted line: the limit of detection (400 CFU/g). Two-tailed Mann–Whitney test was used for statistical analysis (*, *p* < 0.05; ns: not significant).

To compare the virulence of biofilm and planktonic bacteria, mice were infected intranasally with log-phase biofilm and planktonic bacteria. A lethal dose of *A. pleuropneumoniae* (4 × 10^6^ CFU) resulted in acute mortality in mice, with the earliest death occurring even within 12 h and the lethality stabilized within 72 h (Figure 6A). Compared with that infected by planktonic bacteria, the lethality of mice infected by biofilm-grown bacteria was significantly lower (*p* < 0.05), and the onset of mouse death was delayed in the biofilm infection group (Figure 6A). A sub-lethal dose (2 × 10^6^ CFU) was also used to assess bacterial colonization capacity. *A. pleuropneumoniae* proliferated rapidly in mice, with the numbers peaking near 12 h (Figure 6B). There was no significant difference in the bacterial load in vivo between BF and PK at 12 h or 48 h post-infection (Figure 6B). Bacteria were gradually cleared as the infection progressed, and it was seen that the BF-infected group had a higher number of bacteria surviving in vivo than PK at 72 h (Figure 6B). In summary, when used as an inoculum, *A. pleuropneumoniae* biofilm grown bacteria had reduced virulence in mice, and presented a more sustained in vivo colonization than PK.

## Discussion

Bacteria living as biofilms is a common trait of prokaryotes. The structure of biofilm aggregates facilitates bacterial resistance to environmental stresses and survival. Persistent and/or latent infections arising from the biofilm mode of growth have become a major factor in the persistence of disease and its re-emergence [46]. As an important respiratory pathogen, it has been reported that most field isolates of *A. pleuropneumoniae* form biofilms in the laboratory when initially isolated [17]. In pigs, aggregates of *A. pleuropneumoniae* in host lungs is considered a biofilm mode of growth, which may act as a reservoir for outbreaks of infection when exposed to stressors or when other pathogens evade the host [18]. Therefore, the biofilm mode of growth can be considered as facilitating survival of *A. pleuropneumoniae* in the host. In this study, we compared the growth features and gene expression patterns of *A. pleuropneumoniae* grown in biofilms with planktonic cells. A simple in vitro culture method in 12-well plates was used to generate stable *A. pleuropneumoniae* biofilms, and there were obvious differences in growth curves and morphology, compared with that of the planktonic bacteria obtained from shake cultures (Figure 1). Through construction and comparative analysis of WT, Δ*pga* and complimentary strains, it was confirmed that PNAG, a known polysaccharide component of EPS for biofilm formation of many bacteria, was critical for *A. pleuropneumoniae* biofilm formation (Figure 2). Similarly, it was also confirmed that dispersin B (encoded by *dspB*), an enzyme that degrades PNAG was also involved in the regulation of *A. pleuropneumoniae* biofilm formation (Figure 2). These results are consistent with previous studies [24, 25].

Numerous genes have been reported to affect *A. pleuropneumoniae* biofilm formation [27–32]. Among these genes, the H-NS and the TCS CpxAR have been validated to direct regulate the genes *pgaABCD* encoding PNAG synthesis proteins [26, 32]. For other genes, it is still relatively unknown how they influence *A. pleuropneumoniae* formation and/or maintenance. In this study, we used RNA-seq analysis to compare the gene expression of *A. pleuropneumoniae* biofilm and planktonic cultures. This contrasts with a previous study also with 4074, that used microarray hybridization to compare transcriptional profiles of *A. pleuropneumoniae* biofilms at different stages in a drip-flow apparatus and also under static culture [33]. The results indicated that some genes involved in energy metabolism were down-regulated and genes encoding transporters were up-regulated in *A. pleuropneumoniae* biofilms, some of which were also found in this work. For example, the down-regulated genes *ppc* (encodes phosphoenolpyruvate carboxylase), *pckA* (encodes phosphoenolpyruvate carboxykinase), *dmsABC* (encodes anaerobic dimethyl sulfoxide reductase) and genes encoding ribosomal proteins, and the up-regulated genes *znuC* (zinc ABC transporter ATP-binding protein, *fdhC* (formate/nitrite transporter family protein) and *ribD* (5-amino-6-(5-phosphoribosylamino) uracil reductase) were also identified in this study. Unlike hybridization-based microarray technology, RNA-Seq has no upper limit of quantification, which correlates with the number of sequences obtained, and thus it can detect a large range of dynamic expression levels of transcripts [47]. In addition, the advantages of high resolution and low background signal of RNA-seq allow for a high level of reproducibility of gene expression detections, as verified by qRT-PCR in this work (Figures 3C, D). Four times of the number of differentially expressed genes in biofilms were identified in this study compared to that using microarray hybridization, thus expanding our understanding of gene transcription profile of biofilm-grown *A. pleuropneumoniae* (Figure 5).

The gene expression of *A. pleuropneumoniae* grown in biofilms was distinct from growth planktonically. In general, many different metabolic pathways and their components were down-regulated in biofilms. These included, enzymes involved in glycolysis, pentose phosphate pathway, TCA cycle and the oxidative phosphorylation pathway. In addition to aerobic respiration, genes involved in anaerobic respiration using alternative electrons (nitrate, DMSO TMSO) were also down-regulated. In contrast, sulfur and histidine metabolism, iron transport and coenzyme synthesis were up-regulated. Biofilm grown bacteria had significantly up-regulated PNAG synthesis and amino sugar metabolic pathways, which are essential for the formation of cell wall and EPS components of biofilm [48]. These results suggest that *A. pleuropneumoniae* growing in biofilms have a reduced metabolism compared to their planktonic counterparts, reprogramming of metabolic reactions, and a shift to synthesis and translocation of the cell wall and EPS components, most likely occurring to enable survival to stress.

Antibiotic resistance often contributes to the difficulty of biofilm eradication [21]. It has been reported that *A. pleuropneumoniae* field isolates contained multiple antibiotic resistance genes and were positively correlated with their ability to form biofilms [49]. In our study, many antibiotic resistance genes were up-regulated in biofilms (Additional file 4), including *tet* (APPSER1_RS02405) encoding a tetracycline resistance efflux pump [50], *vcaM* (APPSER1_RS06625) encoding an ABC-type multidrug efflux pump [51], *macB* (APPSER1_RS03395) encoding a macrolide transport system ATP-binding/permease protein [52], *rarD* (APPSER1_RS05710) encoding a predicted chloramphenicol resistance permease, and *marC* (APPSER1_RS06555) encoding a predicted multiple antibiotic resistance protein. In addition, a low growth rate also potentially contributes to resistance to antibiotics. Traditional antibiotics usually target bacterial components essential for proliferation, such as proteins involved in cell wall synthesis, DNA synthesis and protein translation. Metabolic processes have also been found to be affected by antibiotics and directly linked to antibiotic efficacy. Bactericidal antibiotics can kill bacteria by inducing their respiration [53]. Biocides and antibiotics can cause elevated ATP and respiration in bacteria by the PTS and the cAMP-Crp cascade, resulting in over-production of reactive oxygen species to a lethal concentration [54]. Therefore, the decreased metabolic activity and ATP biosynthesis found in the biofilm the “resting-mode” can make bacteria more tolerance to antibiotics [4].

It was noted that the genes encoding the major virulence genes of *A. pleuropneumoniae*, the *apxIA* and *apxIIA*, were significantly down-regulated in biofilms. *A. pleuropneumoniae* has four types of RTX toxins, the ApxI, ApxII, ApxIII and ApxIV [15]. The strain used in this study is a virulent strain of serovar 1, which possess both ApxI and ApxII that are both necessary for full virulence of *A. pleuropneumoniae* [55]. Consistent with the down-regulation of the structural genes of both ApxI and ApxII, when used as inocula, biofilm grown bacteria showed reduced lethality for mice (Figure 6A). When the mice were infected with sub-lethal doses, the bacterial numbers recovered from the lungs showed no significant difference between biofilm and planktonic bacteria groups up to 48 h post-infection (Figure 6B), indicating that the difference in lethality was not related to differences in bacterial proliferation in vivo. At 72 h post-infection, the viable number in the lungs of mice infected with biofilm was higher than that infected with planktonic bacteria (Figure 6B), suggesting that the survival time of biofilm was longer in vivo. These results indicate that *A. pleuropneumoniae* growing as as biofilm are less virulent, which is in agreement with previous studies that *A. pleuropneumoniae* biofilm grown cells trigger weaker immune responses [23], and negatively correlates with the severity of lung lesions of pigs [17]. Therefore, it can be inferred that *A. pleuropneumoniae* growing in biofilms down-regulate some main virulence factors and basic metabolic activities, while up-regulating the expression of antibiotic resistance genes and those involved in the maintenance of EPS structures, which can facilitate long-lasting survival in the host.

The biofilm mode of growth is known to be dependent on signal transduction proteins and regulators [8–10]. In this study, in addition to H-NS that was up-regulated in biofilms, two other regulators were identified to be up-regulated, Fnr (HlyX) and Fis. Up-regulated expression of Fnr may be activated by the anaerobic environment inside the biofilm [56]. Many differentially expressed genes involved in the metabolic pathways in *A. pleuropneumoniae* biofilms have been reported to be regulated by Fnr [57]. Proteomic and transcriptomic analyses showed that Fnr (HlyX) induces the expression of genes encoding alternative terminal reductases and hydrogenases which are functional in the anaerobic metabolism of *A. pleuropneumoniae* [57]. However, in this study, Fnr was induced in biofilms, but a lot of its target genes involved in energy and carbohydrate metabolism, including multiple terminal respiratory enzymes, mannose/fructose transporter and the enzymes involved in TCA cycle were down-regulated (Table 1). Simultaneously, *fis* encoding the global regulator Fis [45], was also up-regulated. By analyzing the binding motifs of Fis and Fnr in the database in the upstream regions of the differentially expressed genes in biofilms, we found that most of the upstream regions of these genes had binding sites of both Fis and Fnr, with more genes potentially regulated by Fis (Tables 1, 2). As a typical nucleoid associated protein that functions by binding to specific DNA sequences, Fis has the ability to mask the Fnr binding site, thereby inhibiting the activity of Fnr on the target genes [58, 59]. In *E. coli*, a combined RNA-seq and ChIP-seq analysis has illustrated the extensive role of Fis in regulation of sugar metabolism and transport, as well as the genes involved in cell division, bacteriocin transport and biofilm formation [60]. The overlapping of the regulons of *A. pleuropneumoniae* Fis and Fnr provides an explanation for differential regulation of Fnr target genes in the biofilm transcriptome. Binding of Fis with *nrf* and *nir* promoters is known to inhibit Fnr regulation [61, 62]. Additionally, *A. pleuropneumoniae* Fis and Fnr may regulate each other’s expression since there are binding sites of each other in the upstream regions of their coding genes (Tables 1, 2). A detailed analysis of the cross-regulation of Fis and Fnr on *A. pleuropneumoniae* biofilm gene expression will be the subject of further study.

At present, effective strategies to prevent biofilm formation include the inhibition of sessile bacterial adhesion and initial structure formation by interfering with EPS secretion, such as the utilization of peptidoglycan hydrolase [63], and the use of an antibody against specific EPS components [64]. Lysis of intercellular junctions using DNases and proteases [65, 66] and release of antibiotic-sensitive individual cells from biofilm aggregates by changing the levels of signaling molecules [67, 68] have also been reported to be effective for biofilm treatment. Based on the metabolic behavior of *A. pleuropneumoniae* growing in biofilms, according to the gene expression profile, we speculate that reducing the availability of specific nutrients including oligosaccharides (sialic acid, fucose, galactose*.*), amino acids, iron or sulfur sources may reduce the adhesion and aggregation of *A. pleuropneumoniae* and the formation of biofilms.

**Figure 7:**
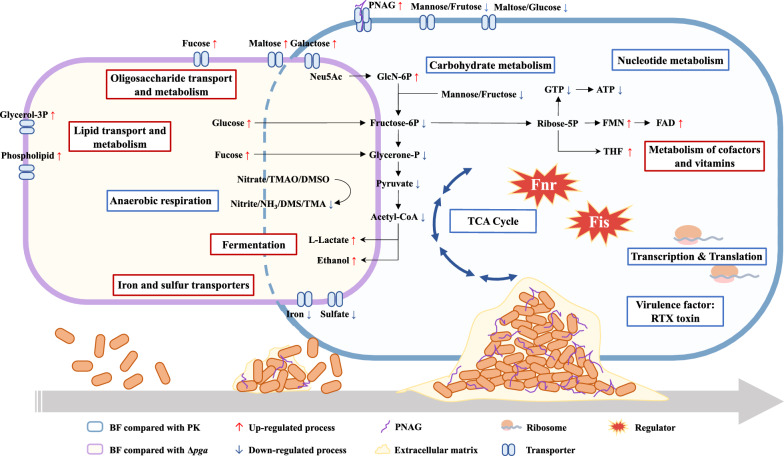
**Metabolic changes of** ***A. pleuropneumoniae*** **living in biofilms compared with planktonic bacteria.** The contents in the purple outlined cell indicate the pathways closely related to adhesion and aggregation during biofilm formation. The pathways in blue outlined cell indicate those altered during the biofilm mode of growth. Red box: up-regulated pathways. Blue box: down-regulated pathways.

In summary, the growth features, morphology, gene expression and virulence of *A. pleuropneumoniae* biofilm grown bacteria were compared with their planktonic counterparts. The results confirmed that *A. pleuropneumoniae* self-aggregated and adhered to surfaces and secreted EPS to form biofilm. The gene expression profiles of *A. pleuropneumoniae* in biofilms were altered extensively, suggesting a lower level of basic metabolic activity, and a fermentation mode of growth accompanied by expression of genes for major EPS synthesis (Figure 7). The regulators Fnr and Fis appear to coordinately have a global regulatory role in maintaining the metabolism of *A. pleuropneumoniae* biofilms. Down-regulation of known virulence genes, e.g., the Apx toxins, is consistent with the reduced virulence of biofilm grown bacteria compared to their planktonic counterparts. These results increase our understanding of *A. pleuropneumoniae* biofilm regulation and development of its clearance strategies.

## Supplementary Information

**Additional file 1: Validation of the mutants Δ*pga*, Δ*dspB*** **and their complementary strains.** (A-B) gDNA was extracted from *A. pleuropneumoniae* wild type (WT), Δ*pga*, Δ*dspB* and their complementary strains. (C-D) RNA was extracted from *A. pleuropneumoniae* WT, Δ*pga*, Δ*dspB* and their complementary strains, and cDNA was synthesized by RT-PCR after gDNA erasure. PCR identification was performed using the indicated primers with gDNA or cDNA as template. Primers *pga*-UF and *pga*-DR bind to the upstream and downstream regions of the *pga* operon, respectively; the primers Δpga-F/R amplify the internal fragment of the pga operon; Primers APPSER1_RS10490 -F/R and APPSER1_RS10515-F/R are used to amplify the upstream and downstream genes of pga operon, respectively. Primers *dspB*-F/R amplify the full-length fragment of the *dspB*; Primers Δ*dspB* -F/R amplifie the internal fragment of the *dspB*; Primers APPSER1_RS05990-F/R and APPSER1_RS06000-F/R are used to amplify the upstream and downstream genes of *dspB*, respectively.

**Additional file 2: Growth curves of *A. pleuropneumoniae* wild type (WT), Δ*pga*, Δ*dspB* and their complementary strains.** The absorbances at OD_600nm_ of bacterial cultures were determined every 2 h. Data are shown as means ± SD from three independent replicates.

**Additional file 3: Bacterial strains, plasmids and primers used in this study.**

**Additional file 4: The differentially expressed genes of BF compared with PK and analysis of transcription factor binding sites on their promoters.**

**Additional file 5: The differentially expressed genes of BF compared with Δ*pga.***

## Data Availability

The dataset supporting the conclusions of this article is available in the NCBI’s Gene Expression Omnibus repository and are accessible through GEO Series accession number GSE221940.
