# Supplementary figures and images for: The morphology and metabolic changes of Actinobacillus pleuropneumoniae during its growth as a biofilm
Source: Vet Res. 2023 May 26;54:42. doi: 10.1186/s13567-023-01173-x (PMC10224306; doi:10.1186/s13567-023-01173-x)

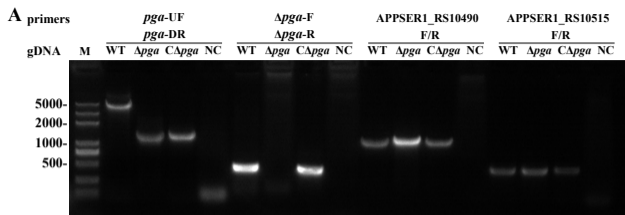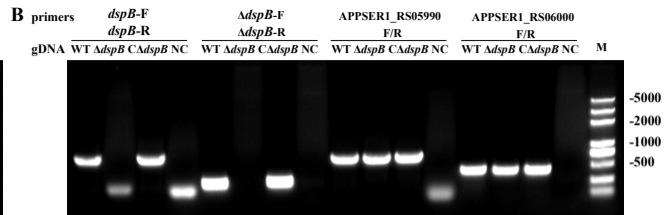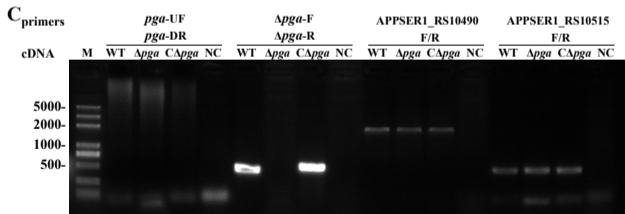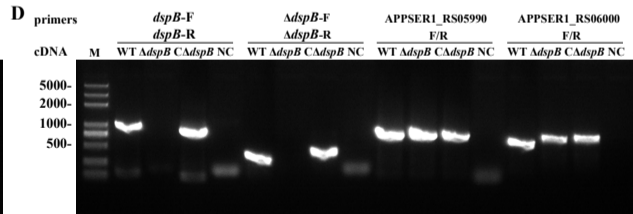

Supplement: Supplementary file 1 — Additional file 1: Validation of the mutants Δpga, ΔdspB and their complementary strains. (A-B) gDNA was extracted from A. pleuropneumoniae wild type (WT), Δpga, ΔdspB and their complementary strains. (C-D) RNA was extracted from A. pleuropneumoniae WT, Δpga, ΔdspB and their complementary strains, and cDNA was synthesized by RT-PCR after gDNA erasure. PCR identification was performed using the indicated primers with gDNA or cDNA as template. Primers pga-UF and pga-DR bind to the upstream and downstream regions of the pga operon, respectively; the primers Δpga-F/R amplify the internal fragment of the pga operon; Primers APPSER1_RS10490 -F/R and APPSER1_RS10515-F/R are used to amplify the upstream and downstream genes of pga operon, respectively. Primers dspB-F/R amplify the full-length fragment of the dspB; Primers ΔdspB -F/R amplifie the internal fragment of the dspB; Primers APPSER1_RS05990-F/R and APPSER1_RS06000-F/R are used to amplify the upstream and downstream genes of dspB, respectively. [file 13567_2023_1173_MOESM1_ESM.pdf]

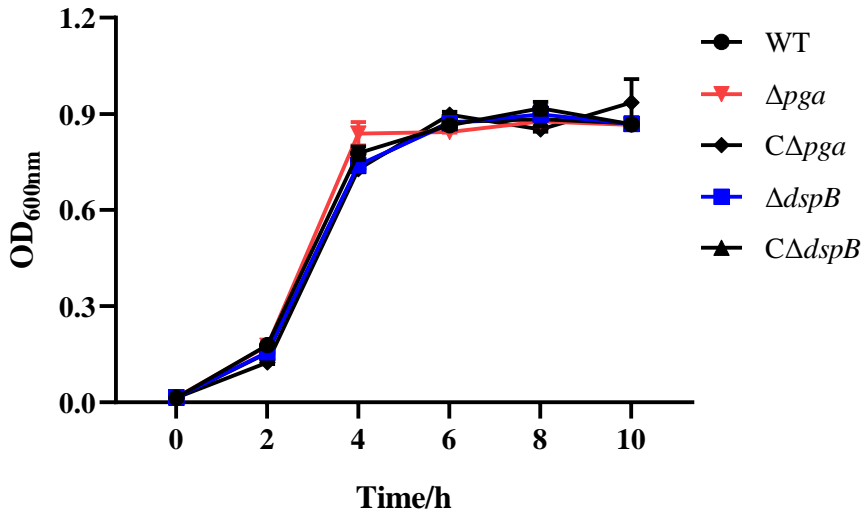

Supplement: Supplementary file 2 — Additional file 2: Growth curves of A. pleuropneumoniae wild type (WT), Δpga, ΔdspB and their complementary strains. The absorbances at OD600nm of bacterial cultures were determined every 2 h. Data are shown as means ± SD from three independent replicates. [file 13567_2023_1173_MOESM2_ESM.pdf]
