# Supplementary material for: The morphology and metabolic changes of Actinobacillus pleuropneumoniae during its growth as a biofilm
Source: Vet Res. 2023 May 26;54:42. doi: 10.1186/s13567-023-01173-x (PMC10224306; doi:10.1186/s13567-023-01173-x)
Supplement: Supplementary file 3 — Additional file 3: Bacterial strains, plasmids and primers used in this study. [file 13567_2023_1173_MOESM3_ESM.docx]

| **Additional file 3. Bacterial strains, plasmids and primers used in this study.** | | |
| --- | --- | --- |
| **Strain/plasmid/primer** | **Characteristics and/or sequences*** | **Source** |
| **Strains** | | |
| ***A. pleuropneumoniae*** | | |
| 4074 | Reference strain of Serovar1, the wild-type | [28] |
| Δ*pga* | *A. pleuropneumoniae* 4074 *pgaABCD* operon deleted mutant | This study |
| CΔ*pga* | *A. pleuropneumoniae* 4074 *pgaABCD operon* complemented strain | This study |
| Δ*dspB* | *A. pleuropneumoniae* 4074 *dspB* deleted mutant | This study |
| CΔ*dspB* | *A. pleuropneumoniae* 4074 *dspB* complemented strain | This study |
| ***E. coli*** | | |
| DH5a | F^-^*endA1 glnV44 thi-1 recA1 relA1 gyrA96 deoRnupG*Φ80d*lacZ*ΔM15Δ(*lacZYA-argF*) U169, *hsdR17*(r _K_^-^m _K_^+^), λ^-^ | TransGen Biotech |
| β2155 | Transconjugation donor. *thrB1004 pro thi strA hsdS lacZ*△*M15(F’ lacZ*△*M15lacI^q^ traD36 proA*^+^ *proB*^+^) *dap*::*erm recA*::*RP4-2-tet*::*Mu-km λpir*, Erm^r^ Tet^r^ Kan^r^ | [35] |
| TOP10 | F-mcrA △(mrr-hsdRMS-mcrBC) φ80lacZ△M15 △lacX74 recA1 ara△139 △(ara-leu) 7697 galU galK rpsL (StrR ) endA1 nupG | Yeasen Biotechnology |
| **Plasmid** | | |
| pEMOC2 | Transconjugation vector: ColE1 *orimob*RP4 *sacB*, Amp^r^ Chl^r^ | [35] |
| pEMOC2-*pga* | pEMOC2 carrying the upstream and downstream regions of *pga* operon | This study |
| pEMOC2-*dspB* | pEMOC2 carrying the upstream and downstream regions of *dspB* | This study |
| pMC-Express | Shuttle vector for protein expression in *A. pleuropneumoniae* | [36] |
| pMC-*pga* | pMC-Express carrying *A. pleuropneumoniae pga* operon | This study |
| pMC-*dspB* | pMC-Express carrying *A. pleuropneumoniae dspB* | This study |
| **Primers** | | |
| **For construction of mutant and complemented strain** | | |
| pEMOC2-testF | GTTATTGGTGCCCTTAAACGCC | This study |
| pEMOC2-testR | GCCAAGCCCGCCGATG | This study |
| *pga*-1 | GGCCCCCCCCTCGAGGTCGACGCTTCGGTATGTTGCTTGGC | This study |
| *pga*-2 | GGTTCGTATGTCGTCGGATAAAGAGCATGGAGAGGCT | This study |
| *pga*-3 | CCATGCTCTTTATCGACGACATACGAACCGATCAA | This study |
| *pga*-4 | GATCCCACCGCGGTGGCGGCCGCTTGTTGCGGTTGCCCTAAGA | This study |
| *pga*-UF | GTGGCGGTATGTTGTTTGACT | This study |
| *pga*-DR | AGCGGGTCAATCAGTCATCG | This study |
| Δ*pga*-F | ATACCGAGCCGAATCTTTCA | This study |
| Δ*pga*-R | AATAGGCGAGTTCTTCTGGT | This study |
| APPSER1_RS10490-F | AATAAACTTGAATGAAACGACCCT | This study |
| APPSER1_RS10490-R | AACTGAACCGGCAAAAATAAACT | This study |
| APPSER1_RS10515-F | GTCGTCCACTTGTAATGGGT | This study |
| APPSER1_RS10515-R | TACGCTTTCGCTTTTTCCG | This study |
| *dspB*-1 | ggcccccccctcgaggtcgacGCAGTTAAAGGTACGATACATTTCATAA | This study |
| *dspB*-2 | ccaatgATTTAGAGGTAGAGTATAGACTCGTTTTGC | This study |
| *dspB*-3 | ctctacctctaaatCATTGGAAATTCTGCTACTATAGCCT | This study |
| *dspB*-4 | gatcccaccgcggtggcggccgcTAAATTGTAGATATAGGCGTGATCCAT | This study |
| *dspB*-F | ATTATGCGCGGTACTTCTCT | This study |
| *dspB*-R | TGCGATTTCGGATCATTAGTT | This study |
| Δ*dspB*-F | ATTGTGCCTGAAGTCGATAGC | This study |
| Δ*dspB*-R | TCTTTGGCTTGAGCGTCAC | This study |
| APPSER1_RS05990-F | TACACAACTTAGTTATAAAGACGC | This study |
| APPSER1_RS05990-R | CTAGATGATTTCAACTTGTTCCG | This study |
| APPSER1_RS06000-F | TGAATTCAACCCCAAAAGCC | This study |
| APPSER1_RS06000-R | TCTGATAGATATTCGTTAAGCCCT | This study |
| pMC-testF | GTAACCGAGCCCGCCTAATG | This study |
| pMC-testR | ATGCTTCCGGCTCGTATGTT | This study |
| pMC-*pga* F | aattctgcagtcgacggtaccCGTTAATTTAAGCTGTTACGTTTGTATG | This study |
| pMC-*pga* R | ttggctgcatctagagcggccgcTTAAGGTTTTTTATGGCGACTTTTAG | This study |
| pMC_*dspB*-F | aattctgcagtcgacggtaccTTTTAAACCTCATTATGAAAAAAGCAA | This study |
| pMC_*dspB*-R | ttggctgcatctagagcggccgcATTTTCTAATGCGATTTCGGATCA | This study |
| **For qRT-PCR** | | |
| q_*apxIA*-F | GACCTGGGTTTGATGCCGTA | This study |
| q_*apxIA*-R | CGCTTTTGTACCTGCATCGG | This study |
| q_*apxIIA*-F | TCGGGTCAAGGAAATGGAGT | This study |
| q_*apxIIA*-R | AATTGCTGCGTTCTTCTCGC | This study |
| q_*dmsA*-F | AGTGAAGGGGCTTGGTATGC | This study |
| q_*dmsA*-R | ATGGGCGTTGGGTAGTAAGC | This study |
| q_*frdA*-F | AAGTTGCCGGTGAAAATGCC | This study |
| q_*frdA*-R | TAAGCGTGCCACCACATCTT | This study |
| q_*manZ*-F | ACGGTGCAGACATTGACGAT | This study |
| q_*manZ*-R | TTCCCCAGAAAATCGGGTCG | This study |
| q_*atpE*-F | TGCACTTGGTACGGCTATCG | This study |
| q_*atpE*-R | GCTGCTTGCTAATTCAGGCT | This study |
| q_*rplX*-F | ATGGCTGCTAAAATCCGTCAAA | This study |
| q_*rplX*-R | GTTACCTTACCACGTTTGCCC | This study |
| q_*hns*-F | CGGCAGAAGAACTCGTAGCA | This study |
| q_*hns*-R | GTATTTTGCAGGACGAGGTGC | This study |
| q_*fnr*-F | TTCCAATCCGGCGATGAACT | This study |
| q_f*nr*-R | TTCGCCGCTTTCACTAATCG | This study |
| q_*fis*-F | GCCCAAGCACAACAAGTGAAC | This study |
| q_*fis*-R | TTCTGTCGGATCTTCACCGTTT | This study |
| q_*pgaA*-F | AAGCGGTTGCCGTGTTAGAA | This study |
| q_*pgaA*-R | ACGTTTGCTCGGTCATGGTT | This study |
| q_APP16S-F | CAAGTCGAACGGTAACGGGA | This study |
| q_APP16S-R | AAGCATTACTCACCCGTCCG | This study |
| qt-*16S rRNA*-F | AAGAAGCACCGGCTAACTCC | This study |
| qt-*16S rRNA*-R | ACTTAATCAACCGCCTGCGT | This study |

Chlr, chloramphenicol resistant; Kanr, kanamycin resistant; Ermr, erythromycin resistant; Tetr, tetracycline; Ampr, ampicillin resistant.
