# Supplementary material for: The morphology and metabolic changes of Actinobacillus pleuropneumoniae during its growth as a biofilm
Source: Vet Res. 2023 May 26;54:42. doi: 10.1186/s13567-023-01173-x (PMC10224306; doi:10.1186/s13567-023-01173-x)
Supplement: Supplementary file 4 — Additional file 4: The differentially expressed genes of BF compared with PK and analysis of transcription factor binding sites on their promoters. [file 13567_2023_1173_MOESM4_ESM.docx]

| **Additional file 4. The differentially expressed genes of BF compared with PK and analysis of transcription factor binding sites^a^ on their promoters.** | | | | | | | | |
| --- | --- | --- | --- | --- | --- | --- | --- | --- |
| **Gene_ID** | **Gene_**  **Name** | **log_2_FC (BF/PK)** | **FDR** | **KO_ID** | **Gene_Description** | **Fnr_MEME**  **Matched Strand^b^** | **Fis_MEME**  **Matched Strand^b^** | **H-NS_MEME**  **Matched Strand^b^** |
| APPSER1_RS01835 | *abiQ* | 3.988 | 8.02E-06 | K19167 | hypothetical protein |  | GCGGATTTTATAAGCAA |  |
| APPSER1_RS10110 | *cysG* | 3.426 | 0.000375 | K02302 | siroheme synthase CysG | TTGATATAAAACAA | TTAGATTTTTTAGCCGA |  |
| APPSER1_RS06715 | *malM* | 3.352 | 3.05E-05 | K05775 | maltose operon protein MalM | TTGATGTAGTTCAC |  |  |
| APPSER1_RS09585 | *nanE* | 3.333 | 8.61E-06 | K01788 | N-acetylmannosamine-6-phosphate 2-epimerase | TTGATAAAAAGCGA | GGTTTAATTTTCACTAA |  |
| APPSER1_RS10325 | *ppdA* | 3.297 | 4.26E-05 | K02679 | type II secretion system GspH family protein |  | GTTCAATATTTGGGCGG |  |
| APPSER1_RS10140 | *lldD* | 3.272 | 0.000114 | K00101 | FMN-dependent L-lactate dehydrogenase LldD | TTGATATTTTTCGT | GGCGAATTACTGCTCGA | GATAAC |
| APPSER1_RS02045 | *ribD* | 3.180 | 4.67E-06 | K11752 | bifunctional diaminohydroxyphosphoribosylaminopyrimidine deaminase/5-amino-6-(5-phosphoribosylamino)uracil reductase RibD | TTGATAATAAAGTA | GATTACTTTATTATCAA |  |
| APPSER1_RS09590 | *nanK* | 3.169 | 6.85E-06 | K00885 | N-acetylmannosamine kinase | TTGATAAAAAGCGA | GGTTTAATTTTCACTAA |  |
| APPSER1_RS07080 | *tbpA* | 3.115 | 2.60E-05 | K16087 | TonB-dependent receptor | TTGATGTTGATTAA | GGGCATTTTATAAGTAA |  |
| APPSER1_RS02050 | *ribE* | 3.104 | 4.67E-06 | K00793 | riboflavin synthase | TTGATAATAAAGTA | GATTACTTTATTATCAA |  |
| APPSER1_RS10125 | *cysW* | 3.071 | 0.000306 | K02047 | sulfate ABC transporter permease subunit CysW |  |  |  |
| APPSER1_RS10120 | *cysU* | 3.070 | 0.000148 | K02046 | sulfate ABC transporter permease subunit CysT |  |  |  |
| APPSER1_RS07450 | *-* | 3.067 | 7.43E-05 | - | - |  |  |  |
| APPSER1_RS10100 | *cysD* | 3.066 | 0.000806 | K00957 | sulfate adenylyltransferase subunit CysD |  |  |  |
| APPSER1_RS03300 | *-* | 3.048 | 6.62E-06 | K06956 | L-cystine transporter | TTGATGTAACGGTT | TAACGATTTATCCGCAG | GATAAC |
| APPSER1_RS09485 | *-* | 3.043 | 8.42E-06 | - | pentapeptide repeat-containing protein | TTGTTCCGGTTCAA |  |  |
| APPSER1_RS08760 | *lipA* | 3.041 | 3.40E-06 | K03644 | lipoyl synthase | TTGTTGTGCATTAT | GCGCACTTTTACAACAT | GATAAC |
| APPSER1_RS10095 | *cysN* | 2.995 | 0.000395 | K00956 | GTP-binding protein |  |  |  |
| APPSER1_RS10090 | *cysJ* | 2.992 | 0.000682 | K00380 | assimilatory sulfite reductase (NADPH) flavoprotein subunit | TTAATTTTTTGCAA | GCGTATTTATAGAGCGA |  |
| APPSER1_RS10105 | *cysH* | 2.865 | 0.000977 | K00390 | phosphoadenylyl-sulfate reductase |  |  |  |
| APPSER1_RS09580 | *-* | 2.819 | 5.05E-06 | - | GDSL-type esterase/lipase family protein | TTGATAAAAAGCGA | GGTTTAATTTTCACTAA |  |
| APPSER1_RS10115 | *sbp* | 2.739 | 0.000333 | K23163 | sulfate ABC transporter substrate-binding protein | TTGATATAAAACAA | TTAGATTTTTTAGCCGA | GATAAC |
| APPSER1_RS09595 | *nanA* | 2.668 | 2.02E-05 | K01639 | N-acetylneuraminate lyase | TTGATAAAAAGCGA | GGTTTAATTTTCACTAA |  |
| APPSER1_RS07690 | *-* | 2.605 | 9.77E-06 | - | transposase |  | GCTTATTTTTAAAGCCA |  |
| APPSER1_RS01925 | *nfo* | 2.574 | 3.80E-05 | K01151 | deoxyribonuclease IV | TTGTTCTAACACAG | TGCCGTTTATTCGGCAA | GATAAC |
| APPSER1_RS09135 | *idnK* | 2.560 | 2.55E-05 | K00851 | gluconokinase | GTGATATAGATCTC | TCCTATTTATCAATCAA |  |
| APPSER1_RS10085 | *cysI* | 2.558 | 0.001389 | K00381 | assimilatory sulfite reductase (NADPH) hemoprotein subunit | TTAATTTTTTGCAA | GCGTATTTATAGAGCGA |  |
| APPSER1_RS06720 | *lamB* | 2.548 | 7.74E-05 | K02024 | maltoporin | TTGATGTAGTTCAC |  |  |
| APPSER1_RS09490 | *hslU* | 2.506 | 8.61E-06 | K03667 | HslU--HslV peptidase ATPase subunit | TTGTTCCGGTTCAA |  |  |
| APPSER1_RS05850 | *bpeT* | 2.451 | 0.000117 | K18900 | hypothetical protein |  |  |  |
| APPSER1_RS09265 | *-* | 2.450 | 0.001238 | K02029 | ABC transporter permease subunit | TTGATTTAACGGTA | TCCTAGTTTTTAAGCGT |  |
| APPSER1_RS09260 | *-* | 2.438 | 0.001071 | K02029 | amino acid ABC transporter permease | TTGATTTAACGGTA | TCCTAGTTTTTAAGCGT |  |
| APPSER1_RS00410 | *exbD* | 2.427 | 0.000359 | K03559 | TonB system transport protein ExbD |  | GGGCGTTTTTTAGATGA |  |
| APPSER1_RS06810 | *-* | 2.418 | 0.000106 | K14445 | DASS family sodium-coupled anion symporter | TTGATGAATTGGAC | GCGCGCTTTTTCGCCAA | GATAAC |
| APPSER1_RS07280 | *-* | 2.359 | 0.001195 | - | - |  |  |  |
| APPSER1_RS06725 | *malK* | 2.334 | 3.89E-05 | K10111 | maltose/maltodextrin ABC transporter ATP-binding protein MalK | TTGATGTAGTTCAC |  |  |
| APPSER1_RS00485 | *tusA* | 2.321 | 1.86E-05 | K04085 | sulfurtransferase TusA |  | TTGCAAAATTTATCCAA |  |
| APPSER1_RS04175 | *-* | 2.275 | 7.13E-05 | - | hypothetical protein | TAGATCTCGATTAA | GAGAATTTTATCGTCAT | GATAAC |
| APPSER1_RS04475 | *-* | 2.275 | 8.48E-06 | - | TolC family protein | TTGATTATTATTGA | TCCGATTATTTGCCCAA |  |
| APPSER1_RS10130 | *cysA* | 2.274 | 0.000565 | K02045 | sulfate ABC transporter ATP-binding protein |  |  |  |
| APPSER1_RS03610 | *epmB* | 2.257 | 0.000137 | K19810 | EF-P beta-lysylation protein EpmB | TTGATCTCTAATAC | GTTTAAAATTTATTCAA |  |
| APPSER1_RS03490 | *-* | 2.254 | 0.000119 | - | primosomal replication protein | TTGATATTTATTAA | GTTGTTTTTTTAAACAT | GATAAC |
| APPSER1_RS10320 | *ppdB* | 2.245 | 0.000416 | K02680 | hypothetical protein |  | GTTCAATATTTGGGCGG |  |
| APPSER1_RS04550 | *dppA* | 2.220 | 0.001286 | K12368 | ABC transporter substrate-binding protein |  |  |  |
| APPSER1_RS08105 | *-* | 2.206 | 0.000318 | - | - |  |  |  |
| APPSER1_RS03865 | *cynT* | 2.194 | 1.52E-05 | K01673 | carbonate dehydratase | TTGATATTACGCGG | GTTCACTTTTTTGACTA |  |
| APPSER1_RS09795 | *-* | 2.190 | 0.000193 | K02013 | ABC transporter ATP-binding protein | TTGATAAAAAACAA |  |  |
| APPSER1_RS10410 | *-* | 2.186 | 0.000154 | - | transposase |  | GCTTATTTTTAAAGCCA |  |
| APPSER1_RS10665 | *adhP* | 2.184 | 0.000212 | K13953 | alcohol dehydrogenase AdhP | TTGATTTTGTTAAA | GGTCGATTTTTCCGCAT |  |
| APPSER1_RS02055 | *ribBA* | 2.176 | 1.26E-05 | K14652 | bifunctional 3%2C4-dihydroxy-2-butanone-4-phosphate synthase/GTP cyclohydrolase II | TTGATAATAAAGTA | GATTACTTTATTATCAA |  |
| APPSER1_RS05975 | *arsR* | 2.166 | 0.000172 | K03892 | metalloregulator ArsR/SmtB family transcription factor |  |  |  |
| APPSER1_RS00415 | *exbB* | 2.145 | 0.000504 | K03561 | TonB-system energizer ExbB |  | GGGCGTTTTTTAGATGA |  |
| APPSER1_RS00145 | *-* | 2.144 | 2.02E-05 | K02035 | ABC transporter substrate-binding protein | TTGACTAAAAATAA | GTCGAATTTCTCTACAA | GATAAC |
| APPSER1_RS00405 | *tonB* | 2.132 | 0.001231 | K03832 | energy transducer TonB |  | GGGCGTTTTTTAGATGA | GATAAC |
| APPSER1_RS10970 | *hisG* | 2.130 | 1.86E-05 | K00765 | ATP phosphoribosyltransferase | TTGTTCTAAAGGAA |  | GATATC |
| APPSER1_RS01435 | *tusE* | 2.118 | 7.24E-05 | K11179 | TusE/DsrC/DsvC family sulfur relay protein | TTAATCTAAAACAA |  | GATAAC |
| APPSER1_RS02440 | *znuC* | 2.103 | 6.83E-06 | K09817 | zinc ABC transporter ATP-binding protein ZnuC | TTGATGGCGATCTT | GGTTGATTTTTTAACGC |  |
| APPSER1_RS09495 | *hslV* | 2.101 | 4.14E-05 | K01419 | ATP-dependent protease subunit HslV |  | CCGCATTATTTGAACAA |  |
| APPSER1_RS01305 | *alr* | 2.097 | 4.12E-05 | K01775 | alanine racemase | TTGATCGTAAACAG | GCCGATTTTTTGCAAAA | GATAAC |
| APPSER1_RS09220 | *fucR* | 2.093 | 0.00028 | K02430 | DeoR/GlpR family DNA-binding transcription regulator |  |  |  |
| APPSER1_RS00655 | *-* | 2.082 | 0.00013 | - | LPS biosynthesis choline kinase | TTGATGTTACTCCA | TAGCATTTTTTATCCGT | GATAAC |
| APPSER1_RS04110 | *-* | 2.029 | 0.000598 | - | - |  |  |  |
| APPSER1_RS02445 | *-* | 2.026 | 2.13E-05 | - | murein DD-endopeptidase MepM | TTGATATTGTGCAG | GTAAAGTTATTCACCAA |  |
| APPSER1_RS03580 | *-* | 2.009 | 0.000201 | - | hypothetical protein | TTGATAATGATTAT | GGATGTGATATGAGCAA |  |
| APPSER1_RS02260 | *rnhA* | 1.999 | 0.001276 | K03469 | ribonuclease HI | TTGATCTAATGCTT | TCTTAATTTTTGACCGC | GATATC |
| APPSER1_RS05960 | *clpB* | 1.989 | 0.000168 | K03695 | ATP-dependent chaperone ClpB | TTGACAAGAATCAA | GTATAAATTCTGCTCGA |  |
| APPSER1_RS10655 | *-* | 1.981 | 7.28E-05 | - | transferrin-binding protein-like solute binding protein |  | CACTACTATTTCGGCAA |  |
| APPSER1_RS07365 | *tehB* | 1.958 | 5.39E-05 | K16868 | SAM-dependent methyltransferase TehB | TTGATGTCACCCCA | GCACATCATACAAGCAA |  |
| APPSER1_RS06910 | *rlmN* | 1.954 | 6.00E-05 | K06941 | bifunctional tRNA (adenosine(37)-C2)-methyltransferase TrmG/ribosomal RNA large subunit methyltransferase RlmN | TTGATCGCAAACAT | TAGTGATTTTTAAACGA | GATAAC |
| APPSER1_RS09915 | *-* | 1.946 | 1.29E-05 | K07082 | endolytic transglycosylase MltG |  | TAGCAATTATTCAACAG | GATAAC |
| APPSER1_RS03430 | *rimP* | 1.946 | 0.000188 | K09748 | ribosome maturation factor RimP |  | GTTGTTTTGTTCACCAA | GATAAC |
| APPSER1_RS10310 | *ppdC* | 1.937 | 6.60E-05 | K02681 | DUF5374 domain-containing protein |  | GTTCAATATTTGGGCGG |  |
| APPSER1_RS03575 | *efeB* | 1.933 | 0.000308 | K16301 | iron uptake transporter deferrochelatase/peroxidase subunit | TTGATAATGATTAT | GGATGTGATATGAGCAA | GATAAC |
| APPSER1_RS05415 | *-* | 1.930 | 2.13E-05 | - | glycosyltransferase |  | GCATATTATTTATTCAC |  |
| APPSER1_RS05510 | *-* | 1.927 | 2.02E-05 | K03316 | sodium:proton antiporter |  |  | GATAAC |
| APPSER1_RS10975 | *hisD* | 1.911 | 5.86E-05 | K00013 | histidinol dehydrogenase |  | GGAGATTTCTAAGCCAA |  |
| APPSER1_RS06905 | *pilF* | 1.911 | 2.36E-05 | K02656 | type IV pilus biogenesis/stability protein PilW |  |  |  |
| APPSER1_RS09600 | *nagB* | 1.904 | 0.000114 | K02564 | glucosamine-6-phosphate deaminase | TTGATAAAAAGCGA | GGTTTAATTTTCACTAA |  |
| APPSER1_RS10940 | *fhuC* | 1.891 | 1.40E-05 | K10829 | ATP-binding cassette domain-containing protein | TTGACTTTTTTCAA | ACACATTTTTTATTCAA | GATAAC |
| APPSER1_RS02375 | *-* | 1.886 | 5.41E-05 | - | transposase | TTGATCGGAATGAA | GCTTATTTTTAAAGCCA |  |
| APPSER1_RS11140 | *-* | 1.886 | 0.00072 | - | glycosyl transferase | TTAATACGTATCAT | TCTGATTTTTGAAGCGT |  |
| APPSER1_RS01620 | *-* | 1.881 | 0.000459 | - | hypothetical protein |  | GCCCAGTTTTTCGGCAG |  |
| APPSER1_RS04025 | *rnb* | 1.871 | 0.00019 | K01147 | exoribonuclease II | TTGATTTTTTACAA | GTTGATTTTTTACAAAA |  |
| APPSER1_RS01740 | *prpC* | 1.868 | 2.02E-05 | K20074 | hypothetical protein | TTAATGGCATTGAA | GCTGAATTTAGAACCGA | GATAAC |
| APPSER1_RS06120 | *tsaD* | 1.868 | 0.000303 | K01409 | tRNA (adenosine(37)-N6)-threonylcarbamoyltransferase complex transferase subunit TsaD |  | GCTCAGCTTTTTCCCGA |  |
| APPSER1_RS09015 | *tusB* | 1.866 | 9.46E-05 | K07237 | sulfurtransferase complex subunit TusB | TTGTTAGATTTCAA | GAAGGCTTATTAAGCTA |  |
| APPSER1_RS09920 | *-* | 1.853 | 0.001627 | - | - |  |  |  |
| APPSER1_RS01770 | *-* | 1.846 | 0.000117 | - | hypothetical protein |  |  |  |
| APPSER1_RS09790 | *-* | 1.840 | 0.000309 | K02015 | iron ABC transporter permease | TTGATAAAAAACAA |  |  |
| APPSER1_RS06155 | *-* | 1.838 | 4.87E-05 | K03310 | sodium:alanine symporter family protein | TTGACGATTTTTAA |  | GATAAC |
| APPSER1_RS05430 | *htpG* | 1.829 | 0.001356 | K04079 | molecular chaperone HtpG | TTGTTGATAACCAA | GCTTATTTTTTGTAAAT | GATAAC |
| APPSER1_RS04590 | *-* | 1.826 | 0.000275 | K09917 | YoaH family protein | TTGATGTGAAGCCG |  | GATAAC |
| APPSER1_RS04050 | *arfA* | 1.819 | 0.000208 | K09890 | ribosome alternative rescue factor ArfA | TTGATCTGGACGAA | TCACATATTTTGTACAG | GATAAC |
| APPSER1_RS03730 | *hisC* | 1.804 | 0.00028 | K00817 | histidinol-phosphate transaminase | TTAATATGAATGTA | GGCGAATATTTCCGAAA | GATAAC |
| APPSER1_RS09270 | *-* | 1.795 | 0.009013 | K02028 | amino acid ABC transporter ATP-binding protein | TTGATTTAACGGTA | TCCTAGTTTTTAAGCGT |  |
| APPSER1_RS05915 | *trxA* | 1.791 | 0.000135 | K03671 | thioredoxin |  | GATGATTATTGCCGCAG |  |
| APPSER1_RS08635 | *exbB* | 1.790 | 3.09E-05 | K03561 | MotA/TolQ/ExbB proton channel family protein | TTAATGAAAATCAT | GCCAATTTTATTAGCAT |  |
| APPSER1_RS09450 | *-* | 1.767 | 4.24E-05 | - | hypothetical protein |  | GGATACTTTTCATACGT |  |
| APPSER1_RS08765 | *lipB* | 1.767 | 5.35E-05 | K03801 | lipoyl(octanoyl) transferase LipB |  | GCTTGTATATTACTCGT | GATAAC |
| APPSER1_RS10770 | *-* | 1.762 | 5.39E-05 | - | - |  |  |  |
| APPSER1_RS10265 | *-* | 1.760 | 4.32E-05 | - | DUF5358 family protein | TTGATTTCTTGCAG | TTCTATTTATTAATCAA | GATAAC |
| APPSER1_RS09805 | *-* | 1.759 | 0.00017 | K02016 | ABC transporter substrate-binding protein | TTGATAAAAAACAA |  |  |
| APPSER1_RS01945 | *-* | 1.757 | 9.64E-05 | K03648 | uracil-DNA glycosylase | TTGACCTAATTCGC | GCTTATTTTTTATTCAA | GATAAC |
| APPSER1_RS01035 | *hofQ* | 1.756 | 2.13E-05 | K02507 | type IV pilus secretin PilQ |  | ATTCGATTTTTAAGCGG |  |
| APPSER1_RS02395 | *lctP* | 1.752 | 0.003323 | K03303 | L-lactate permease | TTGTTTTAACTCTA | GGTTATTTTTTAATCAA |  |
| APPSER1_RS00460 | *nudC* | 1.748 | 7.56E-05 | K03426 | NAD(-) diphosphatase | TTGACCAAGAAGAA | GCCCTTTTTTGACCCAA |  |
| APPSER1_RS10135 | *-* | 1.746 | 0.000204 | - | transposase | GTGATGTCGCTGAT | GCTTATTTTTAAAGCCA |  |
| APPSER1_RS03570 | *efeO* | 1.739 | 0.000925 | K07224 | EfeM/EfeO family lipoprotein | TTGATAATGATTAT | GGATGTGATATGAGCAA |  |
| APPSER1_RS07990 | *-* | 1.737 | 0.00061 | - | - | GTGATCTTGTTCAC | GATCACATTTTTAGCAC |  |
| APPSER1_RS05845 | *-* | 1.736 | 0.000119 | - | carboxymuconolactone decarboxylase family protein | TTGATTTAATAGAT | CCTAAATTTTTTAACAA | GATAAC |
| APPSER1_RS10415 | *dnaJ* | 1.725 | 1.49E-05 | K03686 | molecular chaperone DnaJ |  |  |  |
| APPSER1_RS00910 | *dksA* | 1.724 | 0.000929 | K06204 | RNA polymerase-binding protein DksA |  | GCCAGCTTTTCGTCCAA |  |
| APPSER1_RS09445 | *-* | 1.707 | 7.93E-05 | - | - |  |  |  |
| APPSER1_RS10500 | *pgaB* | 1.705 | 0.003521 | K11931 | poly-beta-1%2C6-N-acetyl-D-glucosamine N-deacetylase PgaB | TTAATTTCAATAAA |  |  |
| APPSER1_RS04585 | *truC* | 1.689 | 0.000527 | K06175 | tRNA pseudouridine(65) synthase TruC | TTGATGTGAAGCCG |  | GATAAC |
| APPSER1_RS07160 | *hslO* | 1.684 | 7.56E-05 | K04083 | Hsp33 family molecular chaperone HslO | TTGATAAATTACAA | GATCTCTTTTTCATCGT |  |
| APPSER1_RS10495 | *-* | 1.679 | 0.000914 | - | poly-beta-1%2C6 N-acetyl-D-glucosamine export porin PgaA | TTAATTTCAATAAA |  |  |
| APPSER1_RS10935 | *-* | 1.677 | 0.000669 | K07034 | GPR1/FUN34/YaaH family transporter | TTGACTTTTTTCAA | ACACATTTTTTATTCAA | GATAAC |
| APPSER1_RS03435 | *nusA* | 1.676 | 0.005553 | K02600 | transcription termination factor NusA |  | GTTGTTTTGTTCACCAA | GATAAC |
| APPSER1_RS05435 | *katE* | 1.674 | 0.003143 | K03781 | catalase | TTGATAGATTTTAT | GCTTATTTTTTGTAAAT |  |
| APPSER1_RS10645 | *-* | 1.674 | 0.000241 | - | - |  |  |  |
| APPSER1_RS00985 | *fis* | 1.672 | 8.91E-05 | K03557 | DNA-binding transcriptional regulator Fis | TTGTTTTAAATTTA | GCCTATTTATTGGACAA | GATAAC |
| APPSER1_RS03755 | *mod* | 1.669 | 3.89E-05 | K07316 | site-specific DNA-methyltransferase | TTGATAAAAATTAC | GCACAACTTATCACTAA |  |
| APPSER1_RS05150 | *-* | 1.658 | 3.05E-05 | - | DUF2625 family protein | TTGTTTTTTCGCAA | TTCCAATTCTTCTGCGA | GATAAC |
| APPSER1_RS01025 | *-* | 1.655 | 8.14E-05 | - | hypothetical protein |  | ATTCGATTTTTAAGCGG |  |
| APPSER1_RS08055 | *psd* | 1.653 | 5.08E-05 | K01613 | archaetidylserine decarboxylase | TTGATTTTGTTTAT | GTATCCTTTTTATTCAA |  |
| APPSER1_RS03080 | *-* | 1.649 | 0.00013 | - | - | TTGTTTTTTAGCAT | GGTTATTTTTAGCCCAA | GATAAC |
| APPSER1_RS02130 | *ksgA* | 1.648 | 5.08E-05 | K02528 | 16S rRNA (adenine(1518)-N(6)/adenine(1519)-N(6))-dimethyltransferase RsmA | TAGATATTTATGAT | TTATGTTTATTTACCAA | GATAAC |
| APPSER1_RS11110 | *dppA* | 1.637 | 0.007566 | K12368 | hypothetical protein |  |  |  |
| APPSER1_RS08615 | *-* | 1.634 | 3.49E-05 | - | hypothetical protein |  |  |  |
| APPSER1_RS01030 | *-* | 1.634 | 7.29E-05 | - | hypothetical protein |  | ATTCGATTTTTAAGCGG |  |
| APPSER1_RS04515 | *-* | 1.634 | 6.98E-05 | K02035 | ABC transporter substrate-binding protein | GTGATCTCTCTCAC | TTAGATATATTCAGCAC | GATAAC |
| APPSER1_RS08770 | *-* | 1.628 | 0.000112 | K09158 | DUF493 family protein YbeD |  | GCTTGTATATTACTCGT | GATAAC |
| APPSER1_RS06590 | *betT* | 1.628 | 3.57E-05 | K02168 | BCCT family transporter | TTAATCAATCTGAA |  | GATATC |
| APPSER1_RS06105 | *cyaY* | 1.623 | 0.00114 | K06202 | iron donor protein CyaY | TTAATACGTATCAT | TCTGATTTTTGAAGCGT |  |
| APPSER1_RS00425 | *ybbN* | 1.617 | 0.000201 | K05838 | co-chaperone YbbN | TTGATACCACTCGC | GTTCAATAATTTTCCAT |  |
| APPSER1_RS09480 | *-* | 1.614 | 0.000511 | - | DUF417 family protein | TTAATGTAAATGTA |  | GATAAC |
| APPSER1_RS03420 | *folC* | 1.612 | 4.50E-05 | K11754 | bifunctional tetrahydrofolate synthase/dihydrofolate synthase | GTGTTATCGAACAA | TAATTTTATTTAAGCAT | GATAAC |
| APPSER1_RS05205 | *-* | 1.611 | 9.46E-05 | - | endonuclease SmrB | TTGATGAGATTCGT | GGATGATTTAAAAACGA |  |
| APPSER1_RS01950 | *virK* | 1.610 | 0.000201 | K09824 | VirK/YbjX family protein | TTGATTATGATCGC |  | GATAAC |
| APPSER1_RS08025 | *hsdS* | 1.604 | 0.00031 | K01154 | restriction endonuclease subunit S |  | GTTTACTATTTCAACAA |  |
| APPSER1_RS03210 | *-* | 1.603 | 5.08E-05 | - | CHY zinc finger protein | TTGATCCGGATCTT | GACGGTTATTTGAGAAA |  |
| APPSER1_RS03480 | *-* | 1.602 | 9.64E-05 | - | nicotinate phosphoribosyltransferase |  | GCTTATTTTTAAAACAT |  |
| APPSER1_RS05710 | *rarD* | 1.599 | 4.37E-05 | K05786 | EamA family transporter RarD | TTGATTAAAAATAG | TGTCTATTTTTAATCAA |  |
| APPSER1_RS03900 | *-* | 1.596 | 7.56E-05 | K07150 | DUF554 domain-containing protein |  |  |  |
| APPSER1_RS09800 | *-* | 1.589 | 0.000413 | - | pseudoazurin | TTGATAAAAAACAA |  |  |
| APPSER1_RS04760 | *-* | 1.588 | 0.000669 | - | - |  |  |  |
| APPSER1_RS02495 | *-* | 1.586 | 1.45E-05 | K09160 | YcgN family cysteine cluster protein | TTGATTTAACGCAT | GCCGGTAATTTAAACGC | GATAAC |
| APPSER1_RS00915 | *pcnB* | 1.573 | 0.000134 | K00970 | polynucleotide adenylyltransferase PcnB | GTGTCGTTTATCAA | TGTCGTTTATCAATCGA |  |
| APPSER1_RS02290 | *ndpA* | 1.568 | 2.55E-05 | K06899 | nucleoid-associated protein YejK | CTGATATTACGCAA |  |  |
| APPSER1_RS07110 | *-* | 1.566 | 9.39E-05 | - | TonB-dependent receptor | TTGGTGTGGAAGAA | GGCGAGTTCTTCCACAC | GATAAC |
| APPSER1_RS01055 | *rhtC* | 1.560 | 0.000129 | K05835 | LysE family transporter | TTGATAAGCAGGAA | GGCGGTATTTTAATCAA |  |
| APPSER1_RS09100 | *fliY* | 1.558 | 0.000941 | K02424 | amino acid ABC transporter substrate-binding protein |  | GGTTATTTTTTCAAAAT | GATAAC |
| APPSER1_RS03745 | *-* | 1.556 | 3.09E-05 | - | SNF2-related protein | TTGATAAAGAATAG | TCTCTATTCTTCATCAA |  |
| APPSER1_RS06510 | *-* | 1.555 | 0.000733 | - | PD-(D/E)XK nuclease family protein | TTAATTAGTATGAA | GTCCGAATATTCTGCAT | GATAAC |
| APPSER1_RS08015 | *-* | 1.547 | 7.08E-05 | - | MerR family transcriptional regulator | TTGATGTCTATAAA | GGCTGTTTTTTCATAAA | GATAAC |
| APPSER1_RS08630 | *exbD* | 1.533 | 0.000219 | K03559 | biopolymer transporter ExbD | TTAATGAAAATCAT | GCCAATTTTATTAGCAT |  |
| APPSER1_RS09130 | *-* | 1.532 | 0.001677 | K03299 | GntP family permease | GTGATATAGATCTC | TCCTATTTATCAATCAA |  |
| APPSER1_RS00205 | *-* | 1.529 | 0.000116 | - | glucose-6-phosphate 1-dehydrogenase family protein |  | GGAGATTTTACAAGCGG |  |
| APPSER1_RS09995 | *-* | 1.524 | 3.03E-05 | - | hypothetical protein | TTAATATAAAGGAA | GGTGATTTCTTACTCAC |  |
| APPSER1_RS10765 | *trmA* | 1.523 | 5.77E-05 | K00557 | tRNA (uridine(54)-C5)-methyltransferase TrmA |  | GATAATGCTTTCAGCAA | GATAAC |
| APPSER1_RS11085 | *hemK* | 1.522 | 4.32E-05 | K02493 | peptide chain release factor N(5)-glutamine methyltransferase | TTAACGGAATTCAA | GGTAAATTTTTCAGAAA |  |
| APPSER1_RS09010 | *tusC* | 1.514 | 0.000232 | K07236 | sulfurtransferase complex subunit TusC | TTGTTAGATTTCAA | GAAGGCTTATTAAGCTA |  |
| APPSER1_RS06635 | *-* | 1.507 | 3.89E-05 | - | MlaD family protein | TTAACGTAGAGCAA |  | GATATC |
| APPSER1_RS02245 | *pbpG* | 1.495 | 0.000178 | K07262 | serine hydrolase | TTGACCTAACGGGA | GCACCTTTTTTACGCAT |  |
| APPSER1_RS00660 | *-* | 1.492 | 0.000202 | - | YcfL family protein | TTGATGTTACTCCA | TAGCATTTTTTATCCGT | GATAAC |
| APPSER1_RS01920 | *-* | 1.490 | 0.000367 | - | molecular chaperone | TTGGTTTTTAGTAA | TGCCAAATTTTCCGCTT | GATAAC |
| APPSER1_RS07975 | *-* | 1.473 | 0.000303 | - | PACE efflux transporter | TTGATGGAAAACGA | TCCTATCTATTGATCAC |  |
| APPSER1_RS03520 | *fnr* | 1.471 | 0.000203 | K01420 | FNR family transcription factor | TTGATTTCAAGCAA | GCTCAAGTTTTCTCTAA |  |
| APPSER1_RS10985 | *rayT* | 1.467 | 0.000233 | K07491 | IS200/IS605 family transposase |  |  |  |
| APPSER1_RS07860 | *-* | 1.466 | 0.00121 | - | transposase |  | GCTTATTTTTAAAGCCA |  |
| APPSER1_RS03655 | *rlmM* | 1.465 | 0.001983 | K06968 | 23S rRNA (cytidine(2498)-2'-O)-methyltransferase RlmM | TTGATTAAAAATAC | GTTGTATTTTTAATCAA |  |
| APPSER1_RS02155 | *-* | 1.463 | 0.000127 | - | hypothetical protein |  |  |  |
| APPSER1_RS05840 | *maf* | 1.443 | 0.000218 | K06287 | septum formation inhibitor Maf | TTGATTTAATAGAT |  | GATAAC |
| APPSER1_RS00920 | *folK* | 1.441 | 0.000135 | K00950 | 2-amino-4-hydroxy-6-hydroxymethyldihydropteridine diphosphokinase | GTGTCGTTTATCAA | TGTCGTTTATCAATCGA |  |
| APPSER1_RS04575 | *-* | 1.424 | 0.000303 | - | Fic family protein | TTGATGTGAAGCCG |  | GATAAC |
| APPSER1_RS10945 | *fhuD* | 1.419 | 0.000185 | K23227 | iron-siderophore ABC transporter substrate-binding protein | TTGACTTTTTTCAA | ACACATTTTTTATTCAA | GATAAC |
| APPSER1_RS08670 | *-* | 1.419 | 9.13E-05 | K01586;K01586 | diaminopimelate decarboxylase |  |  |  |
| APPSER1_RS03690 | *moaD* | 1.418 | 0.000541 | K03636 | molybdopterin synthase sulfur carrier subunit | TTAATAACATTGAA |  |  |
| APPSER1_RS05380 | *ttcA* | 1.418 | 3.80E-05 | K14058 | tRNA 2-thiocytidine(32) synthetase TtcA | TTGATAAACTTTAA |  |  |
| APPSER1_RS10385 | *yfiC* | 1.411 | 3.05E-05 | K15460 | methyltransferase | TTGATGTATTGGTA | GGTCATTTTTAATACGC |  |
| APPSER1_RS08605 | *lyxK* | 1.410 | 0.000121 | K00880 | carbohydrate kinase |  |  |  |
| APPSER1_RS03375 | *tdk* | 1.409 | 7.94E-05 | K00857 | thymidine kinase | TTGATTTCATTGAA | TACCAATTCTTGACCAA | GATAAC |
| APPSER1_RS03440 | *infB* | 1.408 | 0.009218 | K02519 | translation initiation factor IF-2 |  | GTTGTTTTGTTCACCAA | GATAAC |
| APPSER1_RS03455 | *mltB* | 1.406 | 0.000335 | K08305 | lytic murein transglycosylase |  |  | GATAAC |
| APPSER1_RS09005 | *tusD* | 1.394 | 0.000547 | K07235 | sulfurtransferase complex subunit TusD | TTGTTAGATTTCAA | GAAGGCTTATTAAGCTA |  |
| APPSER1_RS05295 | *fhaB* | 1.394 | 8.15E-05 | K15125 | hemagglutinin repeat-containing protein | TTAATCTGAATTAC | GACTATTTTATCGGCAG | GATAAC |
| APPSER1_RS06555 | *marC* | 1.393 | 3.19E-05 | K05595 | Multiple antibiotic resistance (MarC)-related protein [Actinobacillus pleuropneumoniae serovar 11 str. 56153]/ MarC family protein |  |  | GATAAC |
| APPSER1_RS04305 | *-* | 1.391 | 0.002874 | K09922 | DMT family protein | GTGTTCTAACGCAA |  |  |
| APPSER1_RS10885 | *-* | 1.387 | 3.09E-05 | K07280 | surface lipoprotein assembly modifier |  | GATAGCTTTTTTATCAT |  |
| APPSER1_RS07965 | *mglA* | 1.383 | 0.000154 | K10542 | galactose/methyl galactoside ABC transporter ATP-binding protein MglA |  |  |  |
| APPSER1_RS08145 | *mnmE* | 1.378 | 7.02E-05 | K03650 | tRNA uridine-5-carboxymethylaminomethyl(34) synthesis GTPase MnmE |  | TGCAAATTTTTTTGCAA | GATAAC |
| APPSER1_RS00195 | *argS* | 1.377 | 6.99E-05 | K01887 | arginine--tRNA ligase | TTGACTCAAATCCA | GTGCTATTTTTGCACGA |  |
| APPSER1_RS06515 | *hpaI* | 1.377 | 5.39E-05 | K02510 | aldolase/citrate lyase family protein |  |  |  |
| APPSER1_RS02025 | *glpQ* | 1.372 | 0.005193 | K01126 | glycerophosphodiester phosphodiesterase | GTGATGTTATTCAC | GCCGGCTTTTTAAGCAA | GATAAC |
| APPSER1_RS08205 | *-* | 1.371 | 0.000606 | - | hypothetical protein |  | GCATATTTTATGACCTC |  |
| APPSER1_RS06520 | *bfr* | 1.371 | 6.31E-05 | K03594 | bacterioferritin |  | GTATATTATTTCTCCAA |  |
| APPSER1_RS10340 | *-* | 1.367 | 0.000178 | K09911 | macrodomain Ter protein MatP | TTGATCTGTTTAAA | TTCTGTTTTTAACACAA |  |
| APPSER1_RS04820 | *-* | 1.364 | 0.006715 | - | - | TTGGTGGGTAGCAA |  |  |
| APPSER1_RS10370 | *nudH* | 1.352 | 0.0201 | K08311 | RNA pyrophosphohydrolase | TTGATATTTTTTAG | TGCGATTTTATCCCCAT | GATAAC |
| APPSER1_RS03820 | *-* | 1.342 | 8.14E-05 | K02013 | ATP-binding cassette domain-containing protein | TTGATTATTAATAT |  |  |
| APPSER1_RS04755 | *fdhE* | 1.333 | 0.000117 | K02380 | formate dehydrogenase accessory protein FdhE |  | GGTGTTTTTTCGCCCAA | GATAAC |
| APPSER1_RS11155 | *-* | 1.331 | 0.000178 | - | hypothetical protein |  |  |  |
| APPSER1_RS09415 | *-* | 1.329 | 0.000796 | - | helix-turn-helix transcriptional regulator | TTGATATTATTTTA | TGATATTATTTTATCAA |  |
| APPSER1_RS10315 | *-* | 1.326 | 0.002093 | - | DUF2572 family protein |  | GTTCAATATTTGGGCGG |  |
| APPSER1_RS06745 | *malQ* | 1.323 | 0.001438 | K00705 | 4-alpha-glucanotransferase | TTGATGTAGTTCAC | GTTCGATTAGTAAACGT |  |
| APPSER1_RS00925 | *-* | 1.320 | 9.88E-05 | - | YnbE family lipoprotein |  |  |  |
| APPSER1_RS00650 | *gloB* | 1.318 | 0.006286 | K01069 | MBL fold metallo-hydrolase | TTGTTTTTTATCCA | TTTTGTTTTTTATCCAT | GATATC |
| APPSER1_RS04675 | *ppdD* | 1.316 | 3.05E-05 | K02682 | prepilin-type N-terminal cleavage/methylation domain-containing protein | CTGATTTCACTCAC | GTTCAAACTTTATCCGA |  |
| APPSER1_RS10400 | *-* | 1.315 | 3.05E-05 | - | alpha/beta hydrolase |  | GTTTATTATTCCGGCAG |  |
| APPSER1_RS09140 | *gntR* | 1.315 | 8.91E-05 | K06145 | gluconate operon transcriptional repressor GntR | TTGTTATTTTGCAA | TTATGTTTTATTTGCAA | GATAAC |
| APPSER1_RS01020 | *-* | 1.313 | 0.000192 | - | hypothetical protein |  | ATTCGATTTTTAAGCGG |  |
| APPSER1_RS00420 | *glpE* | 1.312 | 0.00069 | K02439 | thiosulfate sulfurtransferase GlpE | TTGATACCACTCGC | GTTCAATAATTTTCCAT |  |
| APPSER1_RS10880 | *-* | 1.311 | 5.08E-05 | - | DMT family transporter | TTAATTTTTCTGAA | TTTTAATTTTTCTGAAA |  |
| APPSER1_RS03695 | *moaE* | 1.306 | 0.000562 | K03635 | molybdopterin synthase catalytic subunit MoaE | TTAATAACATTGAA |  |  |
| APPSER1_RS04360 | *-* | 1.299 | 0.014118 | - | - |  |  |  |
| APPSER1_RS06815 | *parE* | 1.296 | 4.94E-05 | K02622 | DNA topoisomerase IV subunit B | TTGTTCCAACGCAT | CAGTATTTTTTACTCAA |  |
| APPSER1_RS05155 | *-* | 1.295 | 5.72E-05 | K09936 | DMT family transporter | TTGTTTTTTCGCAA | TTCCAATTCTTCTGCGA | GATAAC |
| APPSER1_RS01610 | *ycjF* | 1.293 | 0.000198 | K08990 | YcjF family protein | TTGTTGGTACTCAC |  |  |
| APPSER1_RS01790 | *rapZ* | 1.286 | 0.00045 | K06958 | RNase adapter RapZ | TTGATAATACTAAC | GTCCGATTTTTTCGTAA |  |
| APPSER1_RS02985 | *tadG* | 1.280 | 4.21E-05 | K12515 | hypothetical protein | TTGTTTTTAATTAA | GCTAAAGATTTAGCCAA | GATAAC |
| APPSER1_RS00780 | *nfuA* | 1.270 | 9.92E-05 | K07400 | Fe-S biogenesis protein NfuA | TTGATTTATTTCAA | GGCGGTTTTTTCTACTC |  |
| APPSER1_RS03215 | *-* | 1.267 | 0.000109 | - | DUF1919 domain-containing protein | TTGATCCGGATCTT | GACGGTTATTTGAGAAA |  |
| APPSER1_RS09985 | *-* | 1.264 | 0.000211 | - | type II toxin-antitoxin system Phd/YefM family antitoxin | TTGTTTTAAAATAG | GATCTATTTTAAAACAA |  |
| APPSER1_RS04220 | *-* | 1.260 | 5.39E-05 | - | MFS transporter |  |  |  |
| APPSER1_RS00210 | *rlmB* | 1.258 | 0.000571 | K03218 | 23S rRNA (guanosine(2251)-2'-O)-methyltransferase RlmB | TTATTTTTAATCAA | TTACGTTACTTAAGCAA |  |
| APPSER1_RS02890 | *-* | 1.255 | 6.51E-05 | - | DUF1007 family protein |  |  |  |
| APPSER1_RS11015 | *hisIE* | 1.254 | 0.008323 | K11755 | bifunctional phosphoribosyl-AMP cyclohydrolase/phosphoribosyl-ATP diphosphatase HisIE | TTAATACCTAGCAC | GGCTACGATTTAACCCA |  |
| APPSER1_RS01825 | *-* | 1.252 | 0.00037 | - | TPM domain-containing protein | TTGATAATAAAGAT | GCGGATTTTATAAGCAA |  |
| APPSER1_RS01820 | *-* | 1.251 | 0.000482 | K06872 | TPM domain-containing protein | TTGATAATAAAGAT | GCGGATTTTATAAGCAA |  |
| APPSER1_RS04325 | *-* | 1.247 | 0.000265 | - | DEAD/DEAH box helicase | TTGATTTGGCTTAA | GACCAACTTTTTGCCGT |  |
| APPSER1_RS10020 | *glpT* | 1.245 | 0.000358 | K02445 | MFS transporter |  | GTTCGAATTACAATCAA |  |
| APPSER1_RS00670 | *sitD* | 1.238 | 5.08E-05 | K11606 | metal ABC transporter permease | TTGATGTTACTCCA | TAGCATTTTTTATCCGT | GATAAC |
| APPSER1_RS02905 | *-* | 1.236 | 0.011332 | - | ABC transporter ATP-binding protein |  |  |  |
| APPSER1_RS10395 | *fdhC* | 1.233 | 0.003986 | K21993 | formate/nitrite transporter family protein | TTAATGAAAAGGAT | GGTTATATCTCACGCAT | GATAAC |
| APPSER1_RS02335 | *mreC* | 1.229 | 0.00575 | K03570 | rod shape-determining protein MreC | TTGATAATAAACAA | GTCGGCTTATTCATCGC | GATAAC |
| APPSER1_RS01015 | *-* | 1.228 | 0.00022 | - | hypothetical protein |  | ATTCGATTTTTAAGCGG |  |
| APPSER1_RS01595 | *-* | 1.226 | 0.000133 | - | DMT family transporter | TTGTTGGTACTCAC |  |  |
| APPSER1_RS07275 | *hypA* | 1.222 | 0.000319 | K04651 | hydrogenase maturation nickel metallochaperone HypA | TTAATTTAAAATAA |  |  |
| APPSER1_RS11080 | *-* | 1.222 | 4.32E-05 | - | tetratricopeptide repeat protein | TTAACGGAATTCAA | GGTAAATTTTTCAGAAA |  |
| APPSER1_RS10650 | *-* | 1.222 | 4.83E-05 | - | transferrin-binding protein-like solute binding protein |  | GATCGGCATTTGTGCAA | GATATC |
| APPSER1_RS00675 | *sitC* | 1.221 | 6.41E-05 | K11605 | metal ABC transporter permease | TTGATGTTACTCCA | TAGCATTTTTTATCCGT | GATAAC |
| APPSER1_RS08700 | *-* | 1.220 | 0.000111 | - | stealth family protein | TTGATGAGTTTCAT | GATGTTTTTTTATACGC | GATATC |
| APPSER1_RS09910 | *tmk* | 1.217 | 5.68E-05 | K00943 | dTMP kinase |  | TAGCAATTATTCAACAG |  |
| APPSER1_RS10505 | *pgaC* | 1.212 | 0.009809 | K11936 | poly-beta-1%2C6 N-acetyl-D-glucosamine synthase | TTAATTTCAATAAA |  |  |
| APPSER1_RS10920 | *pdxH* | 1.212 | 0.000462 | K00275 | pyridoxamine 5'-phosphate oxidase |  | GTTTGTTTTTAGTCCAG |  |
| APPSER1_RS05835 | *pbuG* | 1.208 | 0.003094 | K06901 | NCS2 family permease | TTGATAAAGATTTA | TTCTAAATCTTTATCAA |  |
| APPSER1_RS09535 | *-* | 1.206 | 0.016506 | - | - |  |  |  |
| APPSER1_RS09420 | *hipA* | 1.201 | 0.0002 | K07154 | type II toxin-antitoxin system HipA family toxin | TTGATATTATTTTA | TGATATTATTTTATCAA |  |
| APPSER1_RS04580 | *-* | 1.201 | 0.000397 | - | YqcC family protein | TTGATGTGAAGCCG |  | GATAAC |
| APPSER1_RS04595 | *-* | 1.200 | 0.000225 | - | hypothetical protein | TTGATGTGAAGCCG |  | GATAAC |
| APPSER1_RS01250 | *mutT* | 1.194 | 0.000104 | K03574 | 8-oxo-dGTP diphosphatase MutT | TTGATACCTCATAA | GGTCATTTTTTCAGGAA | GATATC |
| APPSER1_RS06165 | *cybC* | 1.193 | 0.014001 | K15536 | cytochrome b562 | TTGATGAAGCGCAT | TACGATTTTTTAATCTA | GATAAC |
| APPSER1_RS04600 | *ygiF* | 1.188 | 9.92E-05 | K18446 | CYTH domain-containing protein | TTGATATGCAATAG | GATCATTTTCCAAACAT | GATAAC |
| APPSER1_RS06375 | *-* | 1.185 | 0.003683 | - | hypothetical protein | TTGATACTACTCCT | TTTTATTTATTCGGCAT |  |
| APPSER1_RS10240 | *ftsN* | 1.182 | 0.012261 | K03591 | cell division protein FtsN | TTGACATTTATCTT | GCGTAAATTTTTAACAA | GATAAC |
| APPSER1_RS06010 | *queE* | 1.181 | 0.000316 | K10026 | 7-carboxy-7-deazaguanine synthase QueE | TTGATTTCAAAGAG | TTGCAATTTTTTTGCAA |  |
| APPSER1_RS01785 | *ubiI* | 1.178 | 0.001269 | K18800 | FAD-dependent 2-octaprenylphenol hydroxylase | TTGATAATACTAAC | GTCCGATTTTTTCGTAA |  |
| APPSER1_RS01935 | *-* | 1.176 | 7.08E-05 | - | hypothetical protein | GTGATTTTACTGAT |  |  |
| APPSER1_RS06330 | *-* | 1.176 | 0.000295 | - | transferrin-binding protein-like solute binding protein | TTGATATTAAACCT | GAGCGAATTATACACAA |  |
| APPSER1_RS04800 | *folE* | 1.175 | 0.019075 | K01495 | GTP cyclohydrolase I FolE | TTGATGTCAATTTG | GTATGATTTTTTACCAC |  |
| APPSER1_RS06950 | *yoeB* | 1.168 | 0.000254 | K19158 | Txe/YoeB family addiction module toxin |  |  |  |
| APPSER1_RS10000 | *rpoE* | 1.166 | 6.82E-05 | K03088 | sigma-70 family RNA polymerase sigma factor | TTAATATAAAGGAA | GGTGATTTCTTACTCAC |  |
| APPSER1_RS04795 | *truA* | 1.165 | 0.005884 | K06173 | tRNA pseudouridine(38-40) synthase TruA | TTGATGTCAATTTG | GTATGATTTTTTACCAC |  |
| APPSER1_RS01605 | *dusC* | 1.164 | 0.000376 | K05541 | tRNA dihydrouridine(16) synthase DusC | TTGTTGGTACTCAC |  |  |
| APPSER1_RS07935 | *napC* | 1.160 | 0.006847 | K02569 | cytochrome c3 family protein | TTAATTTATACCAC | TTTCACTTTATCACCGT |  |
| APPSER1_RS10915 | *srmB* | 1.159 | 0.002048 | K05590 | ATP-dependent RNA helicase SrmB |  | GTTTGTTTTTAGTCCAG |  |
| APPSER1_RS00955 | *tyrA* | 1.156 | 9.56E-05 | K14187 | bifunctional chorismate mutase/prephenate dehydrogenase | TTGATTGAAAGCCT | TCTTAATTTTTCAGCAA | GATAAC |
| APPSER1_RS07970 | *mglC* | 1.155 | 0.000276 | K10541 | galactose/methyl galactoside ABC transporter permease MglC |  |  |  |
| APPSER1_RS03395 | *macB* | 1.155 | 8.14E-05 | K05685 | MacB family efflux pump subunit | TTAATTTTTTGCAA | GGCATATTTTTGTGCAT |  |
| APPSER1_RS04905 | *-* | 1.151 | 0.000321 | - | hypothetical protein |  |  |  |
| APPSER1_RS03180 | *-* | 1.145 | 0.000112 | K07014 | DUF3413 domain-containing protein |  |  |  |
| APPSER1_RS04440 | *lysE* | 1.145 | 9.51E-05 | K06895 | LysE/ArgO family amino acid transporter | TTGATCTTTATAAT |  | GATATC |
| APPSER1_RS10630 | *dtd* | 1.144 | 7.11E-05 | K07560 | D-aminoacyl-tRNA deacylase | TTGATAATGTTTCA | TTTTTATTATTAAACAT | GATAAC |
| APPSER1_RS10930 | *-* | 1.139 | 0.007088 | K00128 | aldehyde dehydrogenase family protein |  | GCTCACATTTTTCACAA | GATAAC |
| APPSER1_RS08625 | *-* | 1.139 | 0.001784 | - | transferrin-binding protein-like solute binding protein | TTAATGAAAATCAT | GCCAATTTTATTAGCAT |  |
| APPSER1_RS05125 | *-* | 1.139 | 0.001031 | - | - |  |  |  |
| APPSER1_RS02765 | *-* | 1.137 | 0.000109 | - | hypothetical protein | TTGATTATGATTAA | GCAGAATATTTAACCAA |  |
| APPSER1_RS06820 | *phoR* | 1.131 | 7.87E-05 | K07636 | phosphate regulon sensor histidine kinase PhoR | TTGTCGAAGATGAA | TTGCAAAATTTTAGCAA |  |
| APPSER1_RS06605 | *tehA* | 1.123 | 0.000154 | K03304 | dicarboxylate transporter/tellurite-resistance protein TehA | TTGATTTCATTCAT | GCCGATTATTTTAGCTA |  |
| APPSER1_RS08610 | *gnl* | 1.119 | 0.004315 | K01053 | SMP-30/gluconolactonase/LRE family protein |  |  |  |
| APPSER1_RS02165 | *hemA* | 1.112 | 0.002147 | K02492 | glutamyl-tRNA reductase | TTGATTTATCACAA | TGTGAATATTTAACCGC | GATAAC |
| APPSER1_RS03635 | *slt* | 1.112 | 7.83E-05 | K08309 | transglycosylase SLT domain-containing protein |  | GGTTTTACTTTGAGCAA |  |
| APPSER1_RS00565 | *putP* | 1.107 | 0.003038 | K11928 | sodium/proline symporter PutP | TTGATTTAATTCGT | GAACAAATCTTCTGCGA |  |
| APPSER1_RS03150 | *parC* | 1.105 | 0.003404 | K02621 | DNA topoisomerase IV subunit A |  | ATCCAATTGTTAAGCAA | GATAAC |
| APPSER1_RS07735 | *dnaG* | 1.102 | 0.000241 | K02316 | DNA primase |  | GGTCAAATTTCAATAAA |  |
| APPSER1_RS01060 | *yfiF* | 1.101 | 0.000136 | K03214 | rRNA methyltransferase |  | GTTCATAATTCCACCGG |  |
| APPSER1_RS02405 | *tet35* | 1.099 | 0.000362 | K18218 | Na-/H- antiporter NhaC family protein |  |  |  |
| APPSER1_RS10905 | *holD* | 1.098 | 0.004005 | K02344 | DNA polymerase III subunit psi |  | GTTTGTTTTTAGTCCAG |  |
| APPSER1_RS04190 | *-* | 1.097 | 0.000179 | - | GIY-YIG nuclease family protein | TTATTCTTGAGCAA |  |  |
| APPSER1_RS05060 | *-* | 1.095 | 0.003492 | - | substrate-binding domain-containing protein | TTGGTCATTATCAT | GGATAAGATTTTTCCAA |  |
| APPSER1_RS03685 | *moaC* | 1.092 | 0.001209 | K03637 | cyclic pyranopterin monophosphate synthase MoaC | TTAATAACATTGAA |  |  |
| APPSER1_RS00200 | *obgE* | 1.091 | 7.13E-05 | K03979 | Obg family GTPase CgtA |  | GGAGATTTTACAAGCGG |  |
| APPSER1_RS03850 | *tgt* | 1.090 | 0.00565 | K00773 | tRNA guanosine(34) transglycosylase Tgt |  |  |  |
| APPSER1_RS10475 | *metJ* | 1.086 | 6.29E-05 | K03764 | met regulon transcriptional regulator MetJ | TTGACGACGAACAA | TGTTACATTTTAACCAA |  |
| APPSER1_RS01465 | *rep* | 1.085 | 0.000309 | K03656 | DNA helicase Rep | TTGATTTTTAAGGC | TTTCATTTTATGCCCTA |  |
| APPSER1_RS02230 | *pgpB* | 1.083 | 0.000146 | K01096 | phosphatase PAP2 family protein |  | CGCCAAATTTTATTCAC |  |
| APPSER1_RS00640 | *ycbB* | 1.082 | 0.007463 | K21470 | L%2CD-transpeptidase family protein | TTGATTTTATATAG | GCAATTCTTTTCATCAA |  |
| APPSER1_RS05395 | *ilvY* | 1.080 | 7.32E-05 | K02521 | HTH-type transcriptional activator IlvY |  |  |  |
| APPSER1_RS06945 | *yefM* | 1.077 | 0.000366 | K19159 | YoeB-YefM toxin-antitoxin system antitoxin YefM | TTGATTCATATGTA |  | GATAAC |
| APPSER1_RS00430 | *dhaM* | 1.069 | 0.042378 | K05881 | PTS-dependent dihydroxyacetone kinase phosphotransferase subunit DhaM | TTAATAAGGATTAC |  |  |
| APPSER1_RS02365 | *sanA* | 1.067 | 0.000273 | K03748 | YdcF family protein | TTGATGTCGCTTAC | GTCTAATTTTTAAGTAT |  |
| APPSER1_RS09785 | *-* | 1.065 | 0.019064 | - | ABC transporter substrate-binding protein | TTGATAAAAAACAA |  | GATAAC |
| APPSER1_RS00225 | *ribF* | 1.064 | 0.002399 | K11753 | bifunctional riboflavin kinase/FAD synthetase | TTGATTTATCTAAA | GTTTTTTATTTAAACGA | GATAAC |
| APPSER1_RS03860 | *cspA* | 1.063 | 0.002708 | K03704 | cold shock domain-containing protein CspD | TTGATATTACGCGG | GCGGAATAGTTAAGCAA |  |
| APPSER1_RS04430 | *envC* | 1.062 | 0.000211 | K22719 | murein hydrolase activator EnvC | GTGATGTTAATTTA | GATGTTAATTTAACCGA | GATATC |
| APPSER1_RS10910 | *rimI* | 1.060 | 0.002653 | K03789 | ribosomal protein S18-alanine N-acetyltransferase |  | GTTTGTTTTTAGTCCAG |  |
| APPSER1_RS07270 | *hypC* | 1.058 | 0.002332 | K04653 | hydrogenase maturation factor HybG | TTGATAAGAATCAT | GTTAATATTCTAATCAA |  |
| APPSER1_RS07900 | *-* | 1.057 | 0.000218 | K00355 | NAD(P)H-dependent oxidoreductase | TTGATAATAATCAA | GGTCATTTTTTAAATGT | GATAAC |
| APPSER1_RS09145 | *lacI* | 1.053 | 0.000443 | K02529 | substrate-binding domain-containing protein | TTGTTATTTTGCAA | GCCTAAATGTTCGGCAA |  |
| APPSER1_RS06005 | *queD* | 1.048 | 0.003076 | K01737 | 6-carboxytetrahydropterin synthase QueD | TTGATTTCAAAGAG | TTGCAATTTTTTTGCAA |  |
| APPSER1_RS01390 | *-* | 1.046 | 8.91E-05 | K01990 | ABC transporter ATP-binding protein | GTAATTTTGCTCAA | GTCTATTTTTTATATAA | GATAAC |
| APPSER1_RS03085 | *-* | 1.045 | 0.000165 | - | ABC transporter ATP-binding protein |  |  |  |
| APPSER1_RS07325 | *folB* | 1.043 | 0.000412 | K01633 | dihydroneopterin aldolase | TTGATATTTTGAAA | TGCTATATTCTCGGCAA | GATAAC |
| APPSER1_RS06625 | *vcaM* | 1.042 | 7.09E-05 | K18893 | ABC transporter ATP-binding protein/permease | TTGATATTTCGCCG | TAATAATTTCTCATCAA |  |
| APPSER1_RS07420 | *rsmI* | 1.041 | 0.000107 | K07056 | 16S rRNA (cytidine(1402)-2'-O)-methyltransferase | TTGATATAAAACTC | CGCTTTATTTTCATCAA | GATATC |
| APPSER1_RS03905 | *-* | 1.040 | 0.001353 | K09979 | hypothetical protein |  |  |  |
| APPSER1_RS09980 | *-* | 1.031 | 0.003137 | - | type II toxin-antitoxin system VapC family toxin | TTGTTTTAAAATAG | GATCTATTTTAAAACAA |  |
| APPSER1_RS02885 | *-* | 1.026 | 0.03641 | - | - |  |  |  |
| APPSER1_RS08080 | *waaC* | 1.025 | 0.008228 | K02841 | lipopolysaccharide heptosyltransferase RfaC | TTGTTCTTATCCAA | GGCGTCGTTTCAAGCAA |  |
| APPSER1_RS04300 | *ispA* | 1.025 | 0.000631 | K00795 | (2E%2C6E)-farnesyl diphosphate synthase | GTGACACAACTCAA | TTTTACTTTTTCACCGC | GATAAC |
| APPSER1_RS05505 | *ppiD* | 1.024 | 0.048861 | K03770 | SurA N-terminal domain-containing protein |  |  | GATAAC |
| APPSER1_RS10595 | *cpdA* | 1.023 | 0.005228 | K03651 | 3'%2C5'-cyclic-AMP phosphodiesterase |  |  | GATAAC |
| APPSER1_RS01050 | *pgpA* | 1.019 | 0.000743 | K01095 | phosphatidylglycerophosphatase A | TTGATAAGCAGGAA | GGCGGTATTTTAATCAA | GATAAC |
| APPSER1_RS08020 | *gshA* | 1.018 | 0.004398 | K01919 | bifunctional glutamate--cysteine ligase GshA/glutathione synthetase GshB | GTGATATTGATCAC | GTATTTTTTATAACCAA |  |
| APPSER1_RS03295 | *-* | 1.013 | 0.000136 | - | Zn-ribbon-containing protein | TTAATTTCGAGTAT | GCTAAATTTTTAACCAA |  |
| APPSER1_RS10235 | *-* | 1.011 | 0.004397 | - | transposase | TTGATTATTCGCAA | GTTCATATTTCAAAAAA |  |
| APPSER1_RS07685 | *fbp* | 1.009 | 0.012999 | K03841 | class 1 fructose-bisphosphatase | GTGACATTGATCAC | GCCGAGTTCTCGAGCAA | GATAAC |
| APPSER1_RS00175 | *-* | 1.008 | 0.000219 | - | isoprenylcysteine carboxylmethyltransferase family protein | TTGATCTTGCGGAC | GGTCAAATTTAGACCGT |  |
| APPSER1_RS04070 | *queA* | 1.002 | 0.000187 | K07568 | tRNA preQ1(34) S-adenosylmethionine ribosyltransferase-isomerase QueA |  | GGCAGTTTTCTAACCAG | GATAAC |
| APPSER1_RS06135 | *-* | 1.001 | 0.000364 | - | YwiC-like family protein | TTGACGGGATTCAT | TTCGAATTTTTTCTCAG |  |
| APPSER1_RS09905 | *holB* | 1.001 | 7.29E-05 | K02341 | DNA polymerase III subunit delta' |  | TAGCAATTATTCAACAG |  |
| APPSER1_RS03120 | *pdxS* | -6.515 | 8.49E-07 | K06215 | pyridoxal 5'-phosphate synthase lyase subunit PdxS | TTAATATTTATTAA | GGTCTATTTATAGCCAA |  |
| APPSER1_RS03125 | *pdxT* | -6.183 | 8.49E-07 | K08681 | pyridoxal 5'-phosphate synthase glutaminase subunit PdxT | TTAATATTTATTAA | GGTCTATTTATAGCCAA |  |
| APPSER1_RS07890 | *-* | -4.449 | 7.74E-05 | - | hypothetical protein |  | GATTATCTGTTATTCAT |  |
| APPSER1_RS03675 | *torY* | -4.092 | 6.83E-06 | K07821 | NapC/NirT family cytochrome c | TTGATCTTAAACAA | GAAGGCATTTCATGCAA |  |
| APPSER1_RS09175 | *dmsA* | -4.008 | 3.40E-06 | K07306 | molybdopterin-dependent oxidoreductase | TTGATCCTTATCAG | TTTTATTATTTCACCTT |  |
| APPSER1_RS04790 | *raiA* | -3.748 | 4.24E-05 | K05809 | ribosome-associated translation inhibitor RaiA | TTGATTATAATCTA | GGATAGATTATAATCAA |  |
| APPSER1_RS01780 | *-* | -3.717 | 4.32E-05 | K09924 | hypothetical protein |  | GTCTGTTTTTTAGTCAG | GATATC |
| APPSER1_RS09190 | *dmsD* | -3.649 | 0.000148 | K23349 | Tat proofreading chaperone DmsD | TTGATCCTTATCAG | TTTTATTATTTCACCTT |  |
| APPSER1_RS09120 | *manY* | -3.511 | 2.80E-05 | K02795 | PTS mannose/fructose/sorbose transporter subunit IIC | TAGATCTAGATCAT | GCTTTTTTTATGATCTA |  |
| APPSER1_RS07165 | *dcuB* | -3.486 | 4.67E-06 | K07792 | anaerobic C4-dicarboxylate transporter | TTGTTTTTGATCAA | TGTTATTATTTAAACGA |  |
| APPSER1_RS09115 | *manZ* | -3.417 | 1.86E-05 | K02796 | PTS mannose transporter subunit IID | TAGATCTAGATCAT | GCTTTTTTTATGATCTA |  |
| APPSER1_RS07240 | *hyaA* | -3.386 | 1.06E-05 | K06282 | hydrogenase 2 small subunit | TTGATAAGAATCAT | GTTAATATTCTAATCAA |  |
| APPSER1_RS01285 | *cysK* | -3.293 | 0.000467 | K01738 | cysteine synthase A | TTAATATAACGGAA | GGCCAGTTTTTGAGCGT | GATAAC |
| APPSER1_RS04555 | *sdaC* | -3.255 | 3.40E-06 | K03837 | serine/threonine transporter | TTGACTTGTTTCAT | CTTTGTTTTTTAAGCGT |  |
| APPSER1_RS07955 | *-* | -3.211 | 0.000425 | K03286 | porin OmpA | TTGTCATCGATTAA | GTCTATTTTTTAATCAA | GATAAC |
| APPSER1_RS09675 | *rplN* | -3.210 | 7.87E-05 | K02874 | 50S ribosomal protein L14 | TTGATTTATAATAG | TTGTAATTTTTAAGCGA |  |
| APPSER1_RS07905 | *napF* | -3.182 | 4.67E-06 | K02572 | ferredoxin-type protein NapF | TTGATAATAATCAA | GTGCGATACTTCAACAA |  |
| APPSER1_RS09180 | *dmsB* | -3.151 | 2.43E-05 | K07307 | dimethylsulfoxide reductase subunit B | TTGATCCTTATCAG | TTTTATTATTTCACCTT |  |
| APPSER1_RS09185 | *dmsC* | -3.133 | 0.000131 | K07308 | dimethyl sulfoxide reductase anchor subunit | TTGATCCTTATCAG | TTTTATTATTTCACCTT |  |
| APPSER1_RS03070 | *afuA* | -3.074 | 6.62E-06 | K02012 | ABC transporter substrate-binding protein | TTGTTGTTTTACAA | TGTGAAATTTTTTGCAA | GATAAC |
| APPSER1_RS01940 | *grcA* | -3.039 | 0.000413 | K06866 | autonomous glycyl radical cofactor GrcA |  | GCTTATTTTTTATTCAA | GATAAC |
| APPSER1_RS09680 | *rplX* | -2.989 | 5.39E-05 | K02895 | 50S ribosomal protein L24 |  |  |  |
| APPSER1_RS10725 | *rpmB* | -2.920 | 7.56E-05 | K02902 | 50S ribosomal protein L28 |  | GATTATTTTTCCTTCGA |  |
| APPSER1_RS03485 | *-* | -2.912 | 0.001805 | - | porin | TTGATATTTATTAA | AACCATTTTTTAAACGT | GATAAC |
| APPSER1_RS05760 | *cyaA* | -2.875 | 7.94E-05 | K05851 | class I adenylate cyclase | TTGACATCTCGCAA |  | GATAAC |
| APPSER1_RS00525 | *nrfA* | -2.796 | 1.12E-05 | K03385 | ammonia-forming nitrite reductase cytochrome c552 subunit | TTGATTTATTTCAA | TGCCGATTTTTATCCGT | GATAAC |
| APPSER1_RS07220 | *hypB* | -2.685 | 8.61E-06 | K04652 | hydrogenase nickel incorporation protein HypB | TTGATCAGGATCAC | GTTTAAATTTTCTCTAA |  |
| APPSER1_RS04135 | *pyrD* | -2.684 | 8.61E-06 | K00254 | quinone-dependent dihydroorotate dehydrogenase | TTGTCTTCAAAGAA | GCACCGTTTTTAACCAA |  |
| APPSER1_RS07910 | *napD* | -2.669 | 1.40E-05 | K02570 | chaperone NapD | TTGATAATAATCAA | GTGCGATACTTCAACAA |  |
| APPSER1_RS01590 | *pal* | -2.604 | 0.001148 | K03640 | peptidoglycan-associated lipoprotein Pal | TTCATCATAATCAT | GCCTAATTTTTGGGCGT |  |
| APPSER1_RS03955 | *secG* | -2.584 | 7.74E-05 | K03075 | preprotein translocase subunit SecG |  | GCCTTATTTTTCAGCAG | GATAAC |
| APPSER1_RS10870 | *rplU* | -2.565 | 6.99E-05 | K02888 | 50S ribosomal protein L21 | TTAATTTTTCTGAA | TTTTAATTTTTCTGAAA | GATAAC |
| APPSER1_RS00605 | *hupA* | -2.562 | 0.000254 | K05787 | HU family DNA-binding protein | TTAATCTAAAATAA | GGTTAAAATTTAACCAA | GATAAC |
| APPSER1_RS07065 | *mdh* | -2.547 | 5.48E-06 | K00024 | malate dehydrogenase | TTGATTTAGATTGA | TACTAATTTATGACCGA | GATAAC |
| APPSER1_RS05955 | *ompW* | -2.520 | 9.77E-06 | K07275 | outer membrane beta-barrel protein | TTGACAAGAATCAA | GTATAAATTCTGCTCGA |  |
| APPSER1_RS04435 | *gpmA* | -2.472 | 1.86E-05 | K01834 | 2%2C3-diphosphoglycerate-dependent phosphoglycerate mutase | TTGTTGTTGTTTAA |  | GATATC |
| APPSER1_RS03950 | *-* | -2.471 | 6.20E-05 | - | - |  |  |  |
| APPSER1_RS07225 | *hypD* | -2.461 | 1.10E-05 | K04654 | hydrogenase formation protein HypD | TTGATCAGGATCAC | GTTTAAATTTTCTCTAA |  |
| APPSER1_RS06930 | *-* | -2.439 | 0.002948 | - | - |  |  |  |
| APPSER1_RS06320 | *-* | -2.422 | 3.09E-05 | K03319 | DASS family sodium-coupled anion symporter | TTGATATTAATTAA | GCCTAATTTAACCTCGA |  |
| APPSER1_RS00535 | *nrfC* | -2.393 | 1.86E-05 | K04014 | cytochrome c nitrite reductase Fe-S protein | TTGATTTATTTCAA | TGCCGATTTTTATCCGT | GATAAC |
| APPSER1_RS06925 | *-* | -2.356 | 0.015095 | - | - |  |  |  |
| APPSER1_RS00530 | *nrfB* | -2.335 | 2.13E-05 | K04013 | cytochrome c nitrite reductase pentaheme subunit | TTGATTTATTTCAA | TGCCGATTTTTATCCGT | GATAAC |
| APPSER1_RS05675 | *focA* | -2.320 | 8.61E-06 | K06212 | formate transporter FocA | TTGATCTGTATCAA |  |  |
| APPSER1_RS09720 | *rplO* | -2.273 | 0.00046 | K02876 | 50S ribosomal protein L15 |  |  |  |
| APPSER1_RS09715 | *rpmD* | -2.265 | 0.000177 | K02907 | 50S ribosomal protein L30 |  |  |  |
| APPSER1_RS09395 | *rplK* | -2.240 | 0.000172 | K02867 | 50S ribosomal protein L11 | TTAATTTCAATCTT | GATCATTTTTCAAGCAA | GATAAC |
| APPSER1_RS01845 | *fruK* | -2.239 | 7.98E-05 | K00882 | 1-phosphofructokinase | GTGATTTAAATCAT | TATCTTTTATTGAGCAA |  |
| APPSER1_RS09740 | *rpsK* | -2.232 | 0.000179 | K02948 | 30S ribosomal protein S11 | TTCACGTTTAACAA |  |  |
| APPSER1_RS08255 | *-* | -2.226 | 0.000204 | - | - |  |  |  |
| APPSER1_RS00785 | *nqrA* | -2.222 | 1.35E-05 | K00346 | Na(-)-translocating NADH-quinone reductase subunit A | TTGATTTATTTCAA | GGCGGTTTTTTCTACTC | GATATC |
| APPSER1_RS09695 | *rpsH* | -2.218 | 0.000127 | K02994 | 30S ribosomal protein S8 |  |  |  |
| APPSER1_RS08100 | *tuf* | -2.204 | 0.000292 | K02358 | elongation factor Tu | TTAACGGAGATCCA |  | GATAAC |
| APPSER1_RS09660 | *rplP* | -2.200 | 6.58E-05 | K02878 | 50S ribosomal protein L16 | TTGACATTAAATAA | GGTTTTTTTATTAGCTA |  |
| APPSER1_RS05545 | *adhE* | -2.199 | 0.000198 | K04072 | bifunctional acetaldehyde-CoA/alcohol dehydrogenase | TTGACATAAATCAA | TGATAATTATTATACGA | GATATC |
| APPSER1_RS09625 | *rplC* | -2.182 | 5.29E-05 | K02906 | 50S ribosomal protein L3 | TTGACATTAAATAA | GGTTTTTTTATTAGCTA |  |
| APPSER1_RS09935 | *acpP* | -2.176 | 0.000176 | K02078 | acyl carrier protein | TTGACCTTTCTTAA | CTGCAATTTTCGATCAA | GATATC |
| APPSER1_RS07245 | *-* | -2.156 | 6.99E-05 | - | hydrogenase 2 operon protein HybA | TTGATAAGAATCAT | GTTAATATTCTAATCAA |  |
| APPSER1_RS00555 | *uraA* | -2.153 | 0.000309 | K02824 | NCS2 family protein | TTAATATTTATCCC | GCTTAATATTTATCCCC |  |
| APPSER1_RS08950 | *glmS* | -2.138 | 2.02E-05 | K00820 | glutamine--fructose-6-phosphate transaminase (isomerizing) |  | GGTTATTTTTTCAGAAA | GATAAC |
| APPSER1_RS00900 | *-* | -2.134 | 0.000447 | - | amidohydrolase family protein |  | GATAGATTTATATGCAA |  |
| APPSER1_RS06410 | *pnuC* | -2.121 | 3.05E-05 | K03811 | nicotinamide riboside transporter PnuC | TTAATGTAAAATAT |  |  |
| APPSER1_RS09665 | *rpmC* | -2.113 | 6.91E-05 | K02904 | 50S ribosomal protein L29 | TTGACATTAAATAA | GGTTTTTTTATTAGCTA |  |
| APPSER1_RS03515 | *uspA* | -2.063 | 0.00022 | K06149 | universal stress protein | TTGATTTCAAGCAA | GCTCAAGTTTTCTCTAA |  |
| APPSER1_RS03935 | *rpsA* | -2.053 | 0.000523 | K02945 | 30S ribosomal protein S1 |  |  |  |
| APPSER1_RS10875 | *rpmA* | -2.005 | 6.91E-05 | K02899 | 50S ribosomal protein L27 | TTAATTTTTCTGAA | TTTTAATTTTTCTGAAA |  |
| APPSER1_RS09730 | *rpmJ* | -2.003 | 7.13E-05 | K02919 | 50S ribosomal protein L36 |  |  |  |
| APPSER1_RS01850 | *fruB* | -2.003 | 0.000172 | K11183 | fused PTS fructose transporter subunit IIA/HPr protein | GTGATTTAAATCAT | TATCTTTTATTGAGCAA |  |
| APPSER1_RS00020 | *-* | -1.999 | 0.000335 | K04565 | superoxide dismutase family protein | TTGATATCGAAGAA |  | GATATC |
| APPSER1_RS09070 | *atpE* | -1.999 | 2.02E-05 | K02110 | F0F1 ATP synthase subunit C | TTGTTGTTAATAAA |  | GATAAC |
| APPSER1_RS09765 | *rimM* | -1.992 | 3.03E-05 | K02860 | ribosome maturation factor RimM |  | GTCCACGTTTTGCACAA | GATAAC |
| APPSER1_RS09640 | *rplB* | -1.987 | 9.51E-05 | K02886 | 50S ribosomal protein L2 | TTGACATTAAATAA | GGTTTTTTTATTAGCTA |  |
| APPSER1_RS09610 | *fumC* | -1.981 | 0.000245 | K01679 | class II fumarate hydratase | GTGACATGGATCAT |  | GATAAC |
| APPSER1_RS09655 | *rpsC* | -1.980 | 7.28E-05 | K02982 | 30S ribosomal protein S3 | TTGACATTAAATAA | GGTTTTTTTATTAGCTA |  |
| APPSER1_RS06205 | *pgi* | -1.977 | 0.000395 | K01810 | glucose-6-phosphate isomerase | TTGTTATAAATCGA |  |  |
| APPSER1_RS06315 | *metE* | -1.966 | 0.00072 | K00549 | 5-methyltetrahydropteroyltriglutamate--homocysteine S-methyltransferase | TTAATATTATGCAA | GCTTGTTTTTTATATAG | GATATC |
| APPSER1_RS09760 | *rpsP* | -1.965 | 3.23E-05 | K02959 | 30S ribosomal protein S16 |  | GTCCACGTTTTGCACAA | GATAAC |
| APPSER1_RS08090 | *rpsG* | -1.960 | 0.000138 | K02992 | 30S ribosomal protein S7 | TTAACGGAGATCCA |  | GATAAC |
| APPSER1_RS09645 | *rpsS* | -1.952 | 5.72E-05 | K02965 | 30S ribosomal protein S19 | TTGACATTAAATAA | GGTTTTTTTATTAGCTA |  |
| APPSER1_RS02080 | *-* | -1.931 | 0.000116 | - | 5'-nucleotidase%2C lipoprotein e(P4) family | TTGATTTAAATTAA | GCAAAAGTTTTACTCAC |  |
| APPSER1_RS04260 | *pckA* | -1.927 | 0.00039 | K01610 | phosphoenolpyruvate carboxykinase (ATP) | TTGACTTCTCGCAA | GTCAAAAATTTAATCAG |  |
| APPSER1_RS08360 | *frdA* | -1.923 | 3.89E-05 | K00244 | fumarate reductase (quinol) flavoprotein subunit | TAGATATTAATCAA | TGTTAATTTTTAAGCAA | GATAAC |
| APPSER1_RS09670 | *rpsQ* | -1.916 | 8.87E-05 | K02961 | 30S ribosomal protein S17 | TTGACATTAAATAA | GGTTTTTTTATTAGCTA |  |
| APPSER1_RS04140 | *prsA* | -1.915 | 1.10E-05 | K00948 | ribose-phosphate pyrophosphokinase | TTGTCTTCAAAGAA | GCACCGTTTTTAACCAA |  |
| APPSER1_RS02300 | *yfiH* | -1.904 | 0.000108 | K05810 | peptidoglycan editing factor PgeF | TTAACGTTACTGAA |  |  |
| APPSER1_RS06085 | *eno* | -1.902 | 0.000224 | K01689 | phosphopyruvate hydratase | GTGATTTAGCTCAC | GTTTATATTTTTAGTAA | GATAAC |
| APPSER1_RS09735 | *rpsM* | -1.897 | 0.000117 | K02952 | 30S ribosomal protein S13 | TTCACGTTTAACAA |  |  |
| APPSER1_RS00790 | *nqrB* | -1.896 | 1.40E-05 | K00347 | NADH:ubiquinone reductase (Na(-)-transporting) subunit B | TTGATTTATTTCAA | GGCGGTTTTTTCTACTC |  |
| APPSER1_RS10040 | *asnA* | -1.894 | 0.000413 | K01914 | aspartate--ammonia ligase | TTAATATAATTCGT | GCTTATTTTACGTGCAT |  |
| APPSER1_RS08565 | *rpsT* | -1.893 | 0.000189 | K02968 | 30S ribosomal protein S20 | TTGATCTTAATCGC | TTTTAATTTTTACACAG | GATAAC |
| APPSER1_RS06140 | *pfkA* | -1.893 | 3.80E-05 | K00850 | 6-phosphofructokinase | TTGACGGGATTCAT | TTCGAATTTTTTCTCAG |  |
| APPSER1_RS09710 | *rpsE* | -1.891 | 0.000104 | K02988 | 30S ribosomal protein S5 |  |  |  |
| APPSER1_RS02280 | *gdhA* | -1.890 | 0.000178 | K00262 | NADP-specific glutamate dehydrogenase | GTGATATTACTTAC | GGTTGTTTAATAGTCAA |  |
| APPSER1_RS02770 | *-* | -1.887 | 0.005193 | - | phage holin%2C lambda family |  | GCAGAATATTTAACCAA | GATAAC |
| APPSER1_RS09745 | *rpsD* | -1.881 | 0.000119 | K02986 | 30S ribosomal protein S4 | TTCACGTTTAACAA |  |  |
| APPSER1_RS09770 | *trmD* | -1.874 | 7.11E-05 | K00554 | tRNA (guanosine(37)-N1)-methyltransferase TrmD |  | GTCCACGTTTTGCACAA | GATAAC |
| APPSER1_RS07590 | *-* | -1.872 | 0.000117 | K07040 | 23S rRNA accumulation protein YceD | TTGATATAAGGTAA |  |  |
| APPSER1_RS09060 | *atpH* | -1.869 | 2.02E-05 | K02113 | F0F1 ATP synthase subunit delta | TTGTTGTTAATAAA |  | GATAAC |
| APPSER1_RS03090 | *rpsB* | -1.857 | 0.000172 | K02967 | 30S ribosomal protein S2 |  | GACGAAATTACCAGCAA |  |
| APPSER1_RS05515 | *rpsO* | -1.854 | 0.001505 | K02956 | 30S ribosomal protein S15 | TTGATGTTAAAGAT | GGTTAAATTTCAACTAA |  |
| APPSER1_RS03670 | *torZ* | -1.848 | 6.01E-05 | K07812 | trimethylamine-N-oxide reductase TorA | TTGATTTAAATTAT |  |  |
| APPSER1_RS07615 | *manX* | -1.840 | 9.59E-05 | K02794 | PTS mannose transporter subunit IIAB |  | GTATGTTATATAACCAA |  |
| APPSER1_RS08835 | *-* | -1.827 | 0.000193 | - | YfcZ/YiiS family protein | TTGATATATATTAG | GGTCGTATTTTCACCGG |  |
| APPSER1_RS00795 | *nqrC* | -1.826 | 3.09E-05 | K00348 | Na(-)-translocating NADH-quinone reductase subunit C | TTGATTTATTTCAA | GGCGGTTTTTTCTACTC |  |
| APPSER1_RS05765 | *fabB* | -1.819 | 0.000192 | K00647 | beta-ketoacyl-ACP synthase I | TTGACATCTCGCAA |  | GATAAC |
| APPSER1_RS09970 | *rpoZ* | -1.817 | 0.001569 | K03060 | DNA-directed RNA polymerase subunit omega |  | TGTCTTTTTATAAACGA |  |
| APPSER1_RS09400 | *rplA* | -1.811 | 0.000192 | K02863 | 50S ribosomal protein L1 | TTAATTTCAATCTT | GATCATTTTTCAAGCAA |  |
| APPSER1_RS06790 | *fbaA* | -1.811 | 0.000101 | K01624 | class II fructose-bisphosphate aldolase | TTGATCTTGTGTAG |  | GATATC |
| APPSER1_RS06795 | *pgk* | -1.800 | 6.76E-05 | K00927 | phosphoglycerate kinase | TTGATCTATATTCG | GATCTATATTCGCCCAA | GATATC |
| APPSER1_RS02790 | *-* | -1.792 | 0.002661 | - | DUF1441 family protein | TTGATTTTTAGGAT | TTTCGATTTCTAGGCAT |  |
| APPSER1_RS07195 | *ptsH* | -1.784 | 0.000272 | K02784 | phosphocarrier protein Hpr | TTAATATTTATCAA | GCGTATTAATTAATCAA | GATAAC |
| APPSER1_RS05670 | *pflD* | -1.783 | 0.0002 | K00656 | formate C-acetyltransferase | TTGATCTGTATCAA |  |  |
| APPSER1_RS09750 | *rpoA* | -1.779 | 0.000171 | K03040 | DNA-directed RNA polymerase subunit alpha | TTCACGTTTAACAA |  |  |
| APPSER1_RS03270 | *rplM* | -1.777 | 0.00028 | K02871 | 50S ribosomal protein L13 | TTAACATGGTACAA | GCTCAAATTTTCAAAAT |  |
| APPSER1_RS09725 | *secY* | -1.776 | 0.000232 | K03076 | preprotein translocase subunit SecY |  |  |  |
| APPSER1_RS06450 | *kefB* | -1.776 | 1.86E-05 | K11747 | monovalent cation:proton antiporter-2 (CPA2) family protein | TTGTTAAAACTCAA | GGTTGAGTTTTAACAAA |  |
| APPSER1_RS02795 | *-* | -1.775 | 0.002264 | - | phage terminase large subunit family protein |  |  |  |
| APPSER1_RS09065 | *atpF* | -1.772 | 2.13E-05 | K02109 | F0F1 ATP synthase subunit B | TTGTTGTTAATAAA |  | GATAAC |
| APPSER1_RS00540 | *nrfD* | -1.769 | 5.35E-05 | K04015 | cytochrome c nitrite reductase subunit NrfD | TTGATTTATTTCAA | TGCCGATTTTTATCCGT | GATAAC |
| APPSER1_RS09620 | *rpsJ* | -1.758 | 9.56E-05 | K02946 | 30S ribosomal protein S10 | TTGACATTAAATAA | GGTTTTTTTATTAGCTA |  |
| APPSER1_RS09690 | *rpsN* | -1.758 | 0.000111 | K02954 | 30S ribosomal protein S14 |  |  |  |
| APPSER1_RS09650 | *rplV* | -1.752 | 4.32E-05 | K02890 | 50S ribosomal protein L22 | TTGACATTAAATAA | GGTTTTTTTATTAGCTA |  |
| APPSER1_RS07915 | *napA* | -1.745 | 4.48E-05 | K02567 | nitrate reductase catalytic subunit NapA | TTGATAATAATCAA | GTGCGATACTTCAACAA |  |
| APPSER1_RS09700 | *rplF* | -1.744 | 0.000198 | K02933 | 50S ribosomal protein L6 |  |  |  |
| APPSER1_RS06400 | *rpsF* | -1.743 | 9.59E-05 | K02990 | 30S ribosomal protein S6 |  | GACGGTATTTTAAACGG | GATAAC |
| APPSER1_RS08095 | *fusA* | -1.741 | 0.000166 | K02355 | elongation factor G | TTAACGGAGATCCA |  | GATAAC |
| APPSER1_RS01840 | *fruA* | -1.741 | 0.000401 | K02770 | fructose-specific PTS transporter subunit EIIC | GTGATTTAAATCAT | TATCTTTTATTGAGCAA |  |
| APPSER1_RS09775 | *rplS* | -1.735 | 4.34E-05 | K02884 | 50S ribosomal protein L19 |  | GTCCACGTTTTGCACAA | GATAAC |
| APPSER1_RS00810 | *nqrF* | -1.727 | 4.24E-05 | K00351 | NADH:ubiquinone reductase (Na(-)-transporting) subunit F | TTGATTTATTTCAA | GGCGGTTTTTTCTACTC |  |
| APPSER1_RS08735 | *-* | -1.715 | 2.55E-05 | K07080 | TAXI family TRAP transporter solute-binding subunit |  |  |  |
| APPSER1_RS02655 | *-* | -1.714 | 0.011184 | - | hypothetical protein |  | GGGCTTTTTTTTAACAA |  |
| APPSER1_RS06395 | *priB* | -1.710 | 0.000131 | K02686 | primosomal replication protein N |  | GACGGTATTTTAAACGG |  |
| APPSER1_RS05350 | *glnE* | -1.708 | 0.000412 | K00982 | bifunctional [glutamate--ammonia ligase]-adenylyl-L-tyrosine phosphorylase/[glutamate--ammonia-ligase] adenylyltransferase | TTGTTGATTTGCAA | GTCCGATTTTTGAAAGA | GATAAC |
| APPSER1_RS08085 | *rpsL* | -1.702 | 0.000227 | K02950 | 30S ribosomal protein S12 |  | GGTAAAATATTCGCCAT | GATAAC |
| APPSER1_RS07205 | *crr* | -1.691 | 0.000117 | K02777 | PTS glucose transporter subunit IIA | TTGTTATTACTGCA |  |  |
| APPSER1_RS10355 | *-* | -1.685 | 0.001659 | - | hypothetical protein | TTGATCGAAAAGAC |  |  |
| APPSER1_RS09630 | *rplD* | -1.677 | 0.000183 | K02926 | 50S ribosomal protein L4 | TTGACATTAAATAA | GGTTTTTTTATTAGCTA |  |
| APPSER1_RS08355 | *frdB* | -1.670 | 6.00E-05 | K00245 | succinate dehydrogenase/fumarate reductase iron-sulfur subunit | TAGATATTAATCAA | TGTTAATTTTTAAGCAA |  |
| APPSER1_RS00805 | *nqrE* | -1.655 | 3.80E-05 | K00350 | NADH:ubiquinone reductase (Na(-)-transporting) subunit E | TTGATTTATTTCAA | GGCGGTTTTTTCTACTC |  |
| APPSER1_RS07200 | *ptsI* | -1.650 | 4.04E-05 | K08483 | phosphoenolpyruvate-protein phosphotransferase PtsI | TTGTTATTACTGCA |  |  |
| APPSER1_RS09055 | *atpA* | -1.649 | 3.09E-05 | K02111 | F0F1 ATP synthase subunit alpha | TTGTTGTTAATAAA |  | GATAAC |
| APPSER1_RS07180 | *scrA* | -1.643 | 0.00114 | K02810 | sucrose-specific PTS transporter subunit IIBC | TTGATATAAAAAAA | GTACAAAATTCAACCAA |  |
| APPSER1_RS00800 | *nqrD* | -1.640 | 2.13E-05 | K00349 | NADH:ubiquinone reductase (Na(-)-transporting) subunit D | TTGATTTATTTCAA | GGCGGTTTTTTCTACTC |  |
| APPSER1_RS09930 | *-* | -1.636 | 0.000288 | - | - |  |  |  |
| APPSER1_RS09405 | *rplJ* | -1.628 | 0.000207 | K02864 | 50S ribosomal protein L10 | TTGATCAACAGCAA | CTTTAATTTCTAATCGA |  |
| APPSER1_RS00970 | *pyk* | -1.617 | 4.42E-05 | K00873 | pyruvate kinase | TTGATTATTTAGAA |  |  |
| APPSER1_RS06390 | *rpsR* | -1.614 | 0.000211 | K02963 | 30S ribosomal protein S18 |  | GACGGTATTTTAAACGG |  |
| APPSER1_RS09705 | *rplR* | -1.610 | 0.000116 | K02881 | 50S ribosomal protein L18 |  |  |  |
| APPSER1_RS03780 | *-* | -1.609 | 0.000165 | K09802 | DUF496 family protein | TTAATATTTATCTT | GTTGTTTCTTTGAACAA |  |
| APPSER1_RS03275 | *rpsI* | -1.600 | 0.000194 | K02996 | 30S ribosomal protein S9 | TTAACATGGTACAA | GCTCAAATTTTCAAAAT |  |
| APPSER1_RS08955 | *-* | -1.598 | 0.000172 | - | DeoR family transcriptional regulator | TTGATACGAAGCCA | TGTCATTCATTAAACAA |  |
| APPSER1_RS08170 | *-* | -1.597 | 0.000245 | - | XRE family transcriptional regulator | TTGATTTCAATGCA | GCTTAATTTTTTGGCAA |  |
| APPSER1_RS10515 | *tpiA* | -1.596 | 7.56E-05 | K01803 | triose-phosphate isomerase | TTGATATAGGTCGC | GCTCACTTTAAAAGCGA | GATAAC |
| APPSER1_RS09685 | *rplE* | -1.595 | 0.000183 | K02931 | 50S ribosomal protein L5 |  |  |  |
| APPSER1_RS04120 | *lpd* | -1.593 | 0.000498 | K00382 | dihydrolipoyl dehydrogenase | TTGATTTTAAACAC | GTCTATAATTTCATCAA | GATAAC |
| APPSER1_RS07150 | *nudE* | -1.589 | 2.55E-05 | K08312 | ADP compounds hydrolase NudE | TTCATGAAAAACAC | GATCTCTTTTTCATCGT |  |
| APPSER1_RS02810 | *-* | -1.586 | 0.0013 | - | Clp protease ClpP |  |  |  |
| APPSER1_RS07010 | *-* | -1.577 | 6.51E-05 | - | hypothetical protein | TTAATGATTATTAC | GGTTAATTTTTGTACTT | GATATC |
| APPSER1_RS08350 | *frdC* | -1.571 | 3.80E-05 | K00246 | fumarate reductase subunit FrdC | TAGATATTAATCAA | TGTTAATTTTTAAGCAA |  |
| APPSER1_RS07620 | *manY* | -1.570 | 0.000141 | K02795 | PTS mannose/fructose/sorbose transporter subunit IIC |  | GTATGTTATATAACCAA |  |
| APPSER1_RS07230 | *hypE* | -1.563 | 7.40E-05 | K04655 | hydrogenase expression/formation protein HypE | TTGATCAGGATCAC | GTTTAAATTTTCTCTAA |  |
| APPSER1_RS08260 | *-* | -1.563 | 0.000838 | - | - |  |  |  |
| APPSER1_RS10530 | *-* | -1.559 | 0.000104 | - | DedA family protein | TTGATATAGGTCGC | GCTCACTTTAAAAGCGA | GATAAC |
| APPSER1_RS06030 | *-* | -1.558 | 0.001258 | - | hypothetical protein | GTGATTTAAAAGAC | TACCGTTTTTTACGCTA | GATAAC |
| APPSER1_RS02820 | *-* | -1.555 | 0.002078 | - | phage tail protein |  |  |  |
| APPSER1_RS04095 | *ushA* | -1.552 | 0.000127 | K11751 | bifunctional UDP-sugar hydrolase/5'-nucleotidase UshA | TTGATTGATATAAA | GGCGGTTTTTATGGCAA | GATATC |
| APPSER1_RS02650 | *-* | -1.552 | 0.004671 | - | hypothetical protein |  |  |  |
| APPSER1_RS02295 | *ridA* | -1.548 | 2.44E-05 | K09022 | RidA family protein | TTAACGTTACTGAA |  | GATATC |
| APPSER1_RS03095 | *tsf* | -1.548 | 0.000212 | K02357 | translation elongation factor Ts |  | GACGAAATTACCAGCAA |  |
| APPSER1_RS09410 | *rplL* | -1.543 | 0.000112 | K02935 | 50S ribosomal protein L7/L12 |  |  |  |
| APPSER1_RS09050 | *atpG* | -1.535 | 3.83E-05 | K02115 | F0F1 ATP synthase subunit gamma | TTGTTGTTAATAAA |  | GATAAC |
| APPSER1_RS02705 | *-* | -1.526 | 0.005689 | - | helix-turn-helix domain-containing protein | TTGTTTACACGCAA |  |  |
| APPSER1_RS00940 | *gloA* | -1.523 | 3.56E-05 | K01759 | lactoylglutathione lyase | GTGACATAAATTAA |  | GATAAC |
| APPSER1_RS07525 | *-* | -1.518 | 0.000245 | - | DUF5718 family protein | TTGTTCTTTAACAA | TATTACTTTTTTATCAG |  |
| APPSER1_RS04480 | *pntB* | -1.517 | 0.000516 | K00325 | Re/Si-specific NAD(P)(-) transhydrogenase subunit beta | TTGACTCAAATCTG | GTCGAATTTTTTACAAA | GATAAC |
| APPSER1_RS07145 | *cysQ* | -1.515 | 3.05E-05 | K01082 | 3'(2')%2C5'-bisphosphate nucleotidase CysQ | TTCATGAAAAACAC | GATCTCTTTTTCATCGT |  |
| APPSER1_RS04865 | *-* | -1.512 | 0.000643 | - | DDE-type integrase/transposase/recombinase |  |  |  |
| APPSER1_RS02815 | *-* | -1.506 | 0.005227 | - | DUF2190 family protein |  |  |  |
| APPSER1_RS05595 | *-* | -1.497 | 4.24E-05 | - | - |  |  |  |
| APPSER1_RS09045 | *atpD* | -1.496 | 5.39E-05 | K02112 | F0F1 ATP synthase subunit beta | TTGTTGTTAATAAA |  | GATAAC |
| APPSER1_RS00185 | *slyB* | -1.493 | 4.39E-05 | K06077 | glycine zipper 2TM domain-containing protein |  | TTCTAATTTATCGGCAA | GATAAC |
| APPSER1_RS09635 | *rplW* | -1.486 | 0.000175 | K02892 | 50S ribosomal protein L23 | TTGACATTAAATAA | GGTTTTTTTATTAGCTA |  |
| APPSER1_RS08345 | *frdD* | -1.480 | 0.000105 | K00247 | fumarate reductase subunit FrdD | TAGATATTAATCAA | TGTTAATTTTTAAGCAA |  |
| APPSER1_RS03470 | *ackA* | -1.477 | 0.000117 | K00925 | acetate kinase | TAAATTTTGAGCAA | TCTTAAATTTTGAGCAA | GATAAC |
| APPSER1_RS03550 | *aspC* | -1.471 | 4.21E-05 | K00813 | aspartate/tyrosine/aromatic aminotransferase | TTGGTATTGAACAC | GTTTATTAATCGCCCAA |  |
| APPSER1_RS04920 | *-* | -1.468 | 0.008463 | - | transglycosylase SLT domain-containing protein |  |  |  |
| APPSER1_RS09210 | *-* | -1.461 | 0.001171 | - | DUF6037 family protein |  |  | GATAAC |
| APPSER1_RS05270 | *hlyA* | -1.452 | 6.34E-05 | K11005 | RTX family hemolysin | TTAACAAAAATTAA |  |  |
| APPSER1_RS03130 | *tadA* | -1.450 | 0.000324 | K11991 | tRNA adenosine(34) deaminase TadA | TTGATAGAAATTGT | GGCTATTTATTCGGCAT |  |
| APPSER1_RS08220 | *-* | -1.449 | 0.00046 | - | peptidoglycan-binding protein LysM | TTGATGATAAAAAA | TTTCAAATTTTCTTCAT |  |
| APPSER1_RS04485 | *pntA* | -1.447 | 6.20E-05 | K00324 | Re/Si-specific NAD(P)(-) transhydrogenase subunit alpha | TTGACTCAAATCTG | GTCGAATTTTTTACAAA | GATAAC |
| APPSER1_RS11105 | *-* | -1.444 | 0.015265 | - | - |  |  |  |
| APPSER1_RS04125 | *aceF* | -1.441 | 0.001556 | K00627 | pyruvate dehydrogenase complex dihydrolipoyllysine-residue acetyltransferase | TTGATTTTAAACAC | GTCTATAATTTCATCAA | GATAAC |
| APPSER1_RS03035 | *cpaB* | -1.438 | 0.002707 | K02279 | SAF domain-containing protein |  |  |  |
| APPSER1_RS07235 | *hypF* | -1.427 | 6.01E-05 | K04656 | carbamoyltransferase HypF | TTGATAAGAATCAT | GGCTCTTTAATATGCAA |  |
| APPSER1_RS06025 | *pepA* | -1.417 | 0.004426 | K01255 | leucyl aminopeptidase | TTGATATGAAAAAA |  | GATATC |
| APPSER1_RS06610 | *hxpB* | -1.416 | 4.32E-05 | K24204 | hexitol phosphatase HxpB | TTGGCTACGATCAA | GACCGCTTTTTGATCGT | GATAAC |
| APPSER1_RS05275 | *apxIIC* | -1.408 | 6.99E-05 | K07389 | RTX-II toxin-activating lysine-acyltransferase ApxIIC | TTAACAAAAATTAA | GTTCTTTTTTTAATCAA | GATAAC |
| APPSER1_RS04875 | *-* | -1.408 | 0.000864 | - | helix-turn-helix domain-containing protein | TTGTTAAAGAGCAA | GGCGGAATTTAGAGCGA |  |
| APPSER1_RS10380 | *ppa* | -1.406 | 9.46E-05 | K01507 | inorganic diphosphatase | TTGACTTTCATCGC | GTTTCCAATTTAAACAA |  |
| APPSER1_RS04695 | *proB* | -1.386 | 0.000103 | K00931 | glutamate 5-kinase | TTGTTGAAAAGGAA | GAATATTATTTTGTCAC |  |
| APPSER1_RS03110 | *frr* | -1.385 | 0.00018 | K02838 | ribosome recycling factor |  | GCTTTCTTTATTTGCAA | GATATC |
| APPSER1_RS04925 | *-* | -1.383 | 0.033389 | - | - |  |  |  |
| APPSER1_RS02635 | *-* | -1.381 | 0.005578 | - | hypothetical protein |  | AGTTAATTTTTCAGCGT | GATAAC |
| APPSER1_RS05935 | *arcD* | -1.378 | 0.000413 | K03758 | basic amino acid/polyamine antiporter | TTGATGTATTCGAT | TGGCAATTTTTTTGCAA | GATAAC |
| APPSER1_RS02825 | *-* | -1.372 | 0.000847 | - | phage tail protein |  |  |  |
| APPSER1_RS04160 | *-* | -1.364 | 0.000635 | - | CidB/LrgB family autolysis modulator |  | GGGCGTTCTTTAATCAG |  |
| APPSER1_RS10440 | *xylG* | -1.353 | 0.002309 | K10545 | D-xylose ABC transporter ATP-binding protein |  | GATTATTTATAAACCGC |  |
| APPSER1_RS00310 | *talA* | -1.343 | 0.000249 | K00616 | transaldolase | TTGTTCTTTAACAA | GTTCTTGTTTTACTCAA | GATAAC |
| APPSER1_RS08915 | *cbiK* | -1.343 | 0.000163 | K10094 | DUF4198 domain-containing protein |  | GCCGATATTTTAAGAAA |  |
| APPSER1_RS09755 | *rplQ* | -1.327 | 0.000112 | K02879 | 50S ribosomal protein L17 | TTCACGTTTAACAA |  |  |
| APPSER1_RS05745 | *ccmG* | -1.326 | 0.000773 | K02199 | redoxin family protein | TTGATCAAATACGA | GACCGCTTCTTCCGCTA | GATAAC |
| APPSER1_RS04915 | *-* | -1.326 | 0.011507 | - | putative holin | TTGATAGTCAGCAT | TATCAATTTTTTCTCAT |  |
| APPSER1_RS06545 | *mmsB* | -1.324 | 7.40E-05 | K00020 | NAD(P)-dependent oxidoreductase | TTGGCATAGATTAA | TATTATTTTTTACTCGG | GATAAC |
| APPSER1_RS09560 | *comM* | -1.315 | 0.004884 | K07391 | YifB family Mg chelatase-like AAA ATPase | GTGACATAGAACAT | GCTTATGATTTACGCAT |  |
| APPSER1_RS07625 | *manZ* | -1.314 | 0.000276 | K02796 | PTS mannose transporter subunit IID |  | GTATGTTATATAACCAA |  |
| APPSER1_RS00115 | *-* | -1.314 | 0.000198 | K03531 | - | TTGATCTAATACTT |  | GATAAC |
| APPSER1_RS09280 | *kpsC* | -1.311 | 6.00E-05 | K07266 | capsular polysaccharide biosynthesis protein | TAGATATAACTCAA | GCCGAATTTAAAACCAA | GATAAC |
| APPSER1_RS03825 | *-* | -1.307 | 7.15E-05 | K07220 | TIGR00153 family protein | TTAATCAAAATCGA | GGTTAATACTTCGGCAT | GATATC |
| APPSER1_RS10750 | *-* | -1.290 | 0.004733 | - | sulfurtransferase-like selenium metabolism protein YedF | TTGACTTATTTTGA | TTCTATTTCTTCATCAT |  |
| APPSER1_RS03465 | *pta* | -1.279 | 0.000309 | K13788 | phosphate acetyltransferase | TAAATTTTGAGCAA | TCTTAAATTTTGAGCAA | GATAAC |
| APPSER1_RS01300 | *-* | -1.279 | 0.000397 | K04564 | superoxide dismutase [Mn] | TTGATTTAGATTAA | GGTTTTTTTATATCCAA |  |
| APPSER1_RS08910 | *cbiL* | -1.279 | 9.59E-05 | K16915 | hypothetical protein |  | GCCGATATTTTAAGAAA |  |
| APPSER1_RS06350 | *yfeX* | -1.279 | 0.000117 | K07223 | Dyp-type peroxidase | TTGACTTATTACAT | GTATATTTTTTAAATGT |  |
| APPSER1_RS07495 | *hda* | -1.275 | 3.19E-05 | K10763 | DnaA regulatory inactivator Hda | TTGATGTCTTTCGT |  | GATAAC |
| APPSER1_RS02255 | *argG* | -1.271 | 0.000907 | K01940 | argininosuccinate synthase | TTGATATAGATGAA | CCCTAATTTTTTACCGA |  |
| APPSER1_RS09040 | *atpC* | -1.269 | 6.91E-05 | K02114 | F0F1 ATP synthase subunit epsilon | TTGTTGTTAATAAA |  | GATAAC |
| APPSER1_RS10445 | *xylH* | -1.266 | 0.002565 | K10544 | sugar ABC transporter permease |  | GATTATTTATAAACCGC |  |
| APPSER1_RS06385 | *rplI* | -1.262 | 0.000215 | K02939 | 50S ribosomal protein L9 |  | GACGGTATTTTAAACGG |  |
| APPSER1_RS07655 | *apxIA* | -1.257 | 0.000125 | K11005 | RTX family hemolysin ApxIA | TTGATTGTAAATAC |  |  |
| APPSER1_RS03385 | *glpX* | -1.256 | 0.000201 | K02446 | class II fructose-bisphosphatase | TTGATTTCATTGAA | TACCAATTCTTGACCAA | GATAAC |
| APPSER1_RS09825 | *torC* | -1.252 | 0.000614 | K03532 | hypothetical protein | TTGATATAAGTCAT | GTTTAATTATTCGGCTA |  |
| APPSER1_RS04165 | *cidA* | -1.251 | 0.000172 | K06518 | CidA/LrgA family protein |  | GGGCGTTCTTTAATCAG |  |
| APPSER1_RS10285 | *mscS* | -1.251 | 0.000397 | K03442 | mechanosensitive ion channel |  | GGTGATTTTATTTGCCA |  |
| APPSER1_RS04780 | *dapA* | -1.245 | 3.80E-05 | K01714 | 4-hydroxy-tetrahydrodipicolinate synthase | TTGATTTTAGTGAA | GGGTTTATTTTAATCGT |  |
| APPSER1_RS00245 | *-* | -1.242 | 0.000842 | - | porin family protein | TTGACTTTACATAA | CCACAATTTTACAACAA |  |
| APPSER1_RS06045 | *dnaE* | -1.241 | 3.89E-05 | K02337 | DNA polymerase III subunit alpha | TTGTTATGTATTAA | TCACGTTTTTTGGTCAG |  |
| APPSER1_RS06980 | *-* | -1.239 | 0.005578 | - | - |  |  |  |
| APPSER1_RS10730 | *rpmG* | -1.238 | 0.000104 | K02913 | 50S ribosomal protein L33 |  | GATTATTTTTCCTTCGA |  |
| APPSER1_RS07695 | *pgdX* | -1.238 | 0.000168 | K24136 | glutathione peroxidase | TTAATTAATAGGAA | TTAGAATATTTAATCGC | GATAAC |
| APPSER1_RS00385 | *ilvE* | -1.234 | 6.34E-05 | K00826 | branched-chain amino acid aminotransferase | TTGTTATGTATTAC | GCTTAATTCTTTTACAA |  |
| APPSER1_RS06020 | *-* | -1.232 | 7.02E-05 | - | hypothetical protein | TTGATTATTAGCTT | GAGGGCTTTTTGTTCAA |  |
| APPSER1_RS10175 | *merT* | -1.213 | 0.00114 | K08363 | membrane protein | TTGTTATATTTCAT | GTAGAAATTTTCTACAA |  |
| APPSER1_RS10035 | *-* | -1.211 | 0.003307 | - | type II toxin-antitoxin system YafO family toxin |  |  |  |
| APPSER1_RS00625 | *cspA* | -1.209 | 0.000992 | K03704 | cold-shock protein | TTGATTTTATATAG | GCAATTCTTTTCATCAA |  |
| APPSER1_RS10755 | *-* | -1.208 | 0.015423 | K07112 | selenium metabolism membrane protein YedE/FdhT | TTGACTTATTTTGA | TTCTATTTCTTCATCAT | GATAAC |
| APPSER1_RS08840 | *-* | -1.206 | 0.000131 | - | tellurite resistance TerB family protein |  | GGTCGTATTTTCATCGG |  |
| APPSER1_RS05425 | *tktA* | -1.206 | 0.000198 | K00615 | transketolase | TTGTTTTTGATTAA | GCATATTATTTATTCAC |  |
| APPSER1_RS03940 | *cmk* | -1.202 | 0.000251 | K00945 | (d)CMP kinase |  |  |  |
| APPSER1_RS01545 | *cydA* | -1.200 | 0.001287 | K00425 | cytochrome ubiquinol oxidase subunit I | TTGACATAAATCAA | TTTGAATATTTGAACGA |  |
| APPSER1_RS07510 | *-* | -1.182 | 0.008043 | - | - |  |  |  |
| APPSER1_RS01815 | *ppc* | -1.180 | 9.46E-05 | K01595 | phosphoenolpyruvate carboxylase | TTGATGATAATTCT | GGCAGAATTATCATCAA | GATAAC |
| APPSER1_RS01550 | *cydB* | -1.180 | 0.000944 | K00426 | cytochrome d ubiquinol oxidase subunit II | TTGACATAAATCAA | TTTGAATATTTGAACGA |  |
| APPSER1_RS01175 | *-* | -1.179 | 7.94E-05 | - | - |  | GGCTGCTTTTGCCGCAT |  |
| APPSER1_RS07980 | *-* | -1.176 | 0.001261 | - | glycine zipper 2TM domain-containing protein | TTGATGGAAAACGA | TCCTATCTATTGATCAC |  |
| APPSER1_RS05235 | *prmB* | -1.175 | 8.14E-05 | K07320 | 50S ribosomal protein L3 N(5)-glutamine methyltransferase | TTAATTTTGCGGAA |  |  |
| APPSER1_RS05750 | *nrfE* | -1.173 | 0.001209 | K04016 | heme lyase NrfEFG subunit NrfE | TTGATCAAATACGA | GACCGCTTCTTCCGCTA | GATAAC |
| APPSER1_RS02000 | *-* | -1.173 | 8.40E-05 | K09909 | DUF2057 domain-containing protein | TTAACTTAAATCAA |  | GATAAC |
| APPSER1_RS00080 | *murD* | -1.172 | 0.000336 | K01925 | UDP-N-acetylmuramoyl-L-alanine--D-glutamate ligase | TTGTTCACGATCGT |  |  |
| APPSER1_RS07600 | *-* | -1.170 | 0.000136 | - | HlyC/CorC family transporter | TTGATATAAGGTAA | GGTGAATCTGTGAGCAA |  |
| APPSER1_RS05665 | *pflA* | -1.168 | 7.94E-05 | K04069 | pyruvate formate lyase 1-activating protein | TTGATAGTAAGTAT | TGTTATTATTTTAGCAG |  |
| APPSER1_RS01670 | *pyrE* | -1.167 | 6.91E-05 | K00762 | orotate phosphoribosyltransferase | TTAATCACAATCAT |  | GATAAC |
| APPSER1_RS05865 | *-* | -1.163 | 0.001776 | - | - |  |  |  |
| APPSER1_RS02400 | *hns* | -1.158 | 0.000471 | K03746 | H-NS histone family protein |  |  |  |
| APPSER1_RS08485 | *-* | -1.157 | 0.002948 | - | - |  |  |  |
| APPSER1_RS03870 | *ilvB* | -1.157 | 7.13E-05 | K01652 | acetolactate synthase 3 large subunit | GTGATAAATCTCAC | GATCATTTCTTCCACTT | GATAAC |
| APPSER1_RS11040 | *sacA* | -1.155 | 0.000729 | K01193 | glycoside hydrolase family 32 protein | TTGATTTAATTCCG | GTCCAATATTTAAACTT |  |
| APPSER1_RS10295 | *-* | -1.147 | 7.93E-05 | - | esterase family protein | TTGATTGTATGGAA | GTTCAGATTCTAAGCTA |  |
| APPSER1_RS08930 | *-* | -1.143 | 0.00064 | - | SLC13 family permease | GTGATGAAGATCTT |  |  |
| APPSER1_RS01645 | *dgt* | -1.140 | 7.66E-05 | K01129 | deoxyguanosinetriphosphate triphosphohydrolase family protein | TTGATTTTACACTA |  | GATAAC |
| APPSER1_RS04840 | *-* | -1.133 | 0.008182 | - | hypothetical protein |  |  |  |
| APPSER1_RS02800 | *-* | -1.133 | 0.006618 | - | hypothetical protein |  |  |  |
| APPSER1_RS08065 | *oapA* | -1.131 | 0.001714 | K07268 | GlcNAc transferase | TTGTCATAAAATAA | GCTCAATTTACAGGCGA | GATAAC |
| APPSER1_RS03975 | *aroC* | -1.124 | 7.83E-05 | K01736 | chorismate synthase | TTGATAATTAAGAT | TGTTATTTTTTATTCAA |  |
| APPSER1_RS03475 | *cpdB* | -1.122 | 0.000118 | K01119 | 2'%2C3'-cyclic-nucleotide 2'-phosphodiesterase | TTGACATGTCTAAA | TCTTAAATTTTGAGCAA | GATAAC |
| APPSER1_RS07250 | *-* | -1.120 | 0.003815 | - | Ni/Fe-hydrogenase cytochrome b subunit | TTGATAAGAATCAT | GTTAATATTCTAATCAA |  |
| APPSER1_RS02830 | *-* | -1.115 | 0.002612 | - | phage tail protein |  |  |  |
| APPSER1_RS00085 | *ftsW* | -1.104 | 0.000198 | K03588 | putative lipid II flippase FtsW | TTGTTCACGATCGT |  |  |
| APPSER1_RS00325 | *-* | -1.103 | 0.004014 | - | - |  |  |  |
| APPSER1_RS09250 | *fucO* | -1.102 | 0.000427 | K00048 | lactaldehyde reductase |  | TTCTGATTTTTTTGCAA |  |
| APPSER1_RS08810 | *-* | -1.099 | 0.004145 | - | amino acid ABC transporter ATP-binding protein | TTGACCAAATTCTA | GTTGAAACTTTCATCGC |  |
| APPSER1_RS07550 | *-* | -1.094 | 0.00013 | K00428 | cytochrome-c peroxidase | TTGACCAAGCTCAA | GCATAATTTTACCGCAA |  |
| APPSER1_RS00120 | *-* | -1.094 | 0.00166 | - | UDP-3-O-acyl-N-acetylglucosamine deacetylase | TTGATCTAATACTT |  |  |
| APPSER1_RS08390 | *anmK* | -1.092 | 0.000119 | K09001 | anhydro-N-acetylmuramic acid kinase |  | CGACGATTTTTCTCCAC | GATAAC |
| APPSER1_RS04860 | *-* | -1.087 | 0.000828 | - | AAA family ATPase |  |  |  |
| APPSER1_RS06380 | *-* | -1.083 | 0.003137 | K09794 | YdcH family protein | TTGATACTACTCCT | TTTTATTTATTCGGCAT |  |
| APPSER1_RS08480 | *-* | -1.075 | 0.032633 | - | - |  |  |  |
| APPSER1_RS03835 | *ygiM* | -1.071 | 7.74E-05 | K07184 | SH3 domain-containing protein | TTAATCAAAATCGA | GGTTAATACTTCGGCAT | GATATC |
| APPSER1_RS06785 | *sapF* | -1.069 | 0.000103 | K19230 | ABC transporter ATP-binding protein | TTCATTTTGATCGC |  | GATAAC |
| APPSER1_RS10855 | *-* | -1.069 | 0.000136 | - | patatin-like phospholipase family protein | TTGATTAAAACGAC | GGTAGAATTATCGCCAA | GATAAC |
| APPSER1_RS00580 | *-* | -1.061 | 0.004605 | - | - | TTGATTAAATTCGG | GGCTATTACACAATCAA | GATAAC |
| APPSER1_RS09215 | *purL* | -1.059 | 0.00035 | K01952 | phosphoribosylformylglycinamidine synthase |  |  | GATAAC |
| APPSER1_RS10525 | *surE* | -1.059 | 0.000186 | K03787 | 5'/3'-nucleotidase SurE | TTGATATAGGTCGC | GCTCACTTTAAAAGCGA | GATAAC |
| APPSER1_RS10805 | *tatB* | -1.059 | 9.92E-05 | K03117 | Sec-independent protein translocase protein TatB | TTGATTTTGCAAAA | GCACGTCTTATCAGCGT | GATAAC |
| APPSER1_RS03830 | *-* | -1.056 | 0.000284 | K03306 | inorganic phosphate transporter | TTAATCAAAATCGA | GGTTAATACTTCGGCAT | GATATC |
| APPSER1_RS05940 | *arcD* | -1.055 | 0.002456 | K03758 | basic amino acid/polyamine antiporter | TTGATGTATTCGAT | TGGCAATTTTTTTGCAA |  |
| APPSER1_RS02560 | *-* | -1.053 | 0.00772 | K17218 | NAD(P)/FAD-dependent oxidoreductase |  |  |  |
| APPSER1_RS07000 | *-* | -1.042 | 0.000693 | - | carboxymuconolactone decarboxylase family protein | TAGATTTTAAAGAA | GTGAAATTTTTAAGAAA | GATATC |
| APPSER1_RS07005 | *pspE* | -1.036 | 0.000851 | K03972 | rhodanese-like domain-containing protein | TAGATTTTAAAGAA | GTGAAATTTTTAAGAAA |  |
| APPSER1_RS10290 | *-* | -1.031 | 0.000117 | - | ATP-grasp domain-containing protein | TTGATTGTATGGAA | GTTCAGATTCTAAGCTA | GATATC |
| APPSER1_RS10675 | *hfq* | -1.027 | 0.002078 | K03666 | RNA chaperone Hfq | TTGACGATAACCGA | GACGATATTTACCACAA | GATAAC |
| APPSER1_RS07805 | *rfbB* | -1.026 | 0.00039 | K01710 | dTDP-glucose 4%2C6-dehydratase | TTAATTTAGTTTAA | GCTCATTTTATTTCCTA | GATAAC |
| APPSER1_RS06435 | *-* | -1.021 | 0.000134 | - | SulP family inorganic anion transporter | TTGGTTTATATCAG | TGTAATTTTTTAATCAA |  |
| APPSER1_RS08680 | *-* | -1.020 | 0.000303 | - | hypothetical protein | TTAATCTTATTTAA | TTTCGTTTTTTAGACAT |  |
| APPSER1_RS08660 | *-* | -1.018 | 0.000615 | K07002 | alpha/beta hydrolase | TTGATAAGAAACTC | GCTGATTTTTTAGGCTA |  |
| APPSER1_RS08905 | *cbiM* | -1.013 | 0.000363 | K02007 | cobalt transporter CbiM |  | GCCGATATTTTAAGAAA |  |
| APPSER1_RS05335 | *-* | -1.011 | 0.004758 | K07085 | putative transporter | TTGTTAAAATTTAA | TCCTTATTTTTAAACAT |  |
| APPSER1_RS10390 | *rraA* | -1.011 | 0.000598 | K02553 | ribonuclease E activity regulator RraA | TTGATTTACATTAT | GGTTATATCTCACGCAT | GATAAC |
| APPSER1_RS07565 | *-* | -1.010 | 0.002417 | K09923 | YggL family protein | TTGATATAAGGTAA |  |  |
| APPSER1_RS07680 | *mpl* | -1.009 | 7.72E-05 | K02558 | UDP-N-acetylmuramate:L-alanyl-gamma-D-glutamyl-meso-diaminopimelate ligase | GTGACATTGATCAC |  | GATAAC |
| APPSER1_RS05945 | *-* | -1.008 | 7.94E-05 | - | hypothetical protein | TTGATGTATTCGAT | TGGCAATTTTTTTGCAA |  |
| APPSER1_RS00345 | *dppA* | -1.007 | 0.000168 | K12368 | ABC transporter substrate-binding protein |  | GACGACTTATTTCCCAA |  |
| APPSER1_RS02755 | *-* | -1.002 | 0.005035 | K07504 | type I restriction enzyme HsdR N-terminal domain-containing protein |  |  |  |
| APPSER1_RS10800 | *tatA* | -1.000 | 0.000127 | K03116 | Sec-independent protein translocase subunit TatA | TTGATTTTGCAAAA | GCACGTCTTATCAGCGT | GATAAC |

^a,^ The reference binding motifs of Fnr and Fis were extracted from DB database (<http://regulondb.ccg.unam.mx/>). The Fnr motif used for searching wasTTGATNWNDMKCAH. The Fis motif used for searching was GNNBRWWWWWTVNNCRN. The H-NS motif used for searching was RATAWH [45].

^b,^ The MEME-FIMO online tool (Find Individual Motif Occurences: https://meme-suite.org/meme/tools/fimo, *p*-value < 0.001) was used to search the promoter regions of selected genes for the specific binding motifs of Fnr, Fis and H-NS.
