# Supplementary material for: The morphology and metabolic changes of Actinobacillus pleuropneumoniae during its growth as a biofilm
Source: Vet Res. 2023 May 26;54:42. doi: 10.1186/s13567-023-01173-x (PMC10224306; doi:10.1186/s13567-023-01173-x)
Supplement: Supplementary file 5 — Additional file 5: The differentially expressed genes of BF compared with Δpga. [file 13567_2023_1173_MOESM5_ESM.docx]

| **Additional file 5. The differentially expressed genes of BF compared with Δ*pga*.** | | | | | |
| --- | --- | --- | --- | --- | --- |
| **Gene_ID** | **Gene_Name** | **log_2_FC (BF/Δ*pga*)** | **FDR** | **KO_ID** | **Gene_Description** |
| APPSER1_RS10505 | *pgaC* | 8.793 | 7.70E-07 | K11936 | poly-beta-1%2C6 N-acetyl-D-glucosamine synthase |
| APPSER1_RS10500 | *pgaB* | 8.658 | 1.23E-06 | K11931 | poly-beta-1%2C6-N-acetyl-D-glucosamine N-deacetylase PgaB |
| APPSER1_RS10495 | *pgaA* | 7.890 | 7.66E-08 | - | poly-beta-1%2C6 N-acetyl-D-glucosamine export porin PgaA |
| APPSER1_RS10510 | *pgaD* | 6.756 | 5.66E-08 | - | hypothetical protein |
| APPSER1_RS09585 | *nanE* | 4.965 | 4.96E-08 | K01788 | N-acetylmannosamine-6-phosphate 2-epimerase |
| APPSER1_RS07450 | *-* | 4.813 | 5.29E-06 | - | - |
| APPSER1_RS09580 | *-* | 4.793 | 4.96E-08 | - | GDSL-type esterase/lipase family protein |
| APPSER1_RS06725 | *malK* | 4.791 | 7.66E-08 | K10111 | maltose/maltodextrin ABC transporter ATP-binding protein MalK |
| APPSER1_RS09590 | *nanK* | 4.758 | 4.96E-08 | K00885 | N-acetylmannosamine kinase |
| APPSER1_RS10115 | *sbp* | 4.499 | 4.96E-08 | K23163 | sulfate ABC transporter substrate-binding protein |
| APPSER1_RS10120 | *cysU* | 4.257 | 4.96E-08 | K02046 | sulfate ABC transporter permease subunit CysT |
| APPSER1_RS10125 | *cysW* | 4.225 | 2.54E-07 | K02047 | sulfate ABC transporter permease subunit CysW |
| APPSER1_RS10140 | *lldD* | 3.743 | 1.05E-06 | K00101 | FMN-dependent L-lactate dehydrogenase LldD |
| APPSER1_RS09920 | *-* | 3.651 | 4.99E-06 | - | - |
| APPSER1_RS04110 | *-* | 3.372 | 1.93E-05 | - | - |
| APPSER1_RS04515 | *-* | 3.324 | 4.15E-07 | K02035 | ABC transporter substrate-binding protein |
| APPSER1_RS09595 | *nanA* | 3.237 | 7.26E-07 | K01639 | N-acetylneuraminate lyase |
| APPSER1_RS02045 | *ribD* | 3.149 | 3.90E-07 | K11752 | bifunctional diaminohydroxyphosphoribosylaminopyrimidine deaminase/5-amino-6-(5-phosphoribosylamino)uracil reductase RibD |
| APPSER1_RS10110 | *cysG* | 3.129 | 1.31E-06 | K02302 | siroheme synthase CysG |
| APPSER1_RS07965 | *mglA* | 3.087 | 1.90E-06 | K10542 | galactose/methyl galactoside ABC transporter ATP-binding protein MglA |
| APPSER1_RS06155 | *-* | 3.044 | 4.02E-07 | K03310 | sodium:alanine symporter family protein |
| APPSER1_RS04760 | *-* | 3.004 | 2.36E-06 | - | - |
| APPSER1_RS06720 | *lamB* | 2.938 | 1.05E-06 | K02024 | maltoporin |
| APPSER1_RS10325 | *ppdA* | 2.877 | 9.07E-07 | K02679 | type II secretion system GspH family protein |
| APPSER1_RS09600 | *nagB* | 2.821 | 4.83E-06 | K02564 | glucosamine-6-phosphate deaminase |
| APPSER1_RS06590 | *betT* | 2.819 | 3.57E-07 | K02168 | BCCT family transporter |
| APPSER1_RS07280 | *-* | 2.818 | 2.29E-05 | - | - |
| APPSER1_RS09225 | *fucI* | 2.782 | 4.02E-07 | K01818 | L-fucose isomerase |
| APPSER1_RS06405 | *purD* | 2.712 | 6.66E-06 | K01945 | phosphoribosylamine--glycine ligase |
| APPSER1_RS10320 | *ppdB* | 2.682 | 4.89E-07 | K02680 | hypothetical protein |
| APPSER1_RS09145 | *lacI* | 2.570 | 3.14E-06 | K02529 | substrate-binding domain-containing protein |
| APPSER1_RS09220 | *fucR* | 2.547 | 2.36E-06 | K02430 | DeoR/GlpR family DNA-binding transcription regulator |
| APPSER1_RS07975 | *-* | 2.527 | 7.61E-07 | - | PACE efflux transporter |
| APPSER1_RS10100 | *cysD* | 2.487 | 9.79E-06 | K00957 | sulfate adenylyltransferase subunit CysD |
| APPSER1_RS07970 | *mglC* | 2.411 | 8.27E-07 | K10541 | galactose/methyl galactoside ABC transporter permease MglC |
| APPSER1_RS10105 | *cysH* | 2.408 | 7.33E-06 | K00390 | phosphoadenylyl-sulfate reductase |
| APPSER1_RS06730 | *malE* | 2.391 | 7.53E-06 | K10108 | maltose/maltodextrin ABC transporter substrate-binding protein MalE |
| APPSER1_RS04675 | *ppdD* | 2.387 | 3.65E-06 | K02682 | prepilin-type N-terminal cleavage/methylation domain-containing protein |
| APPSER1_RS07045 | *mlaF* | 2.376 | 3.14E-06 | K02065 | phospholipid ABC transporter ATP-binding protein MlaF |
| APPSER1_RS05835 | *pbuG* | 2.363 | 9.49E-07 | K06901 | NCS2 family permease |
| APPSER1_RS10130 | *cysA* | 2.275 | 6.19E-06 | K02045 | sulfate ABC transporter ATP-binding protein |
| APPSER1_RS07940 | *yidC* | 2.248 | 7.33E-06 | K03217 | membrane protein insertase YidC |
| APPSER1_RS06915 | *-* | 2.240 | 4.60E-05 | - | - |
| APPSER1_RS10370 | *nudH* | 2.229 | 4.33E-05 | K08311 | RNA pyrophosphohydrolase |
| APPSER1_RS02050 | *ribE* | 2.216 | 3.35E-06 | K00793 | riboflavin synthase |
| APPSER1_RS10935 | *-* | 2.211 | 1.03E-05 | K07034 | GPR1/FUN34/YaaH family transporter |
| APPSER1_RS03430 | *rimP* | 2.162 | 2.29E-05 | K09748 | ribosome maturation factor RimP |
| APPSER1_RS10665 | *adhP* | 2.142 | 5.43E-06 | K13953 | alcohol dehydrogenase AdhP |
| APPSER1_RS05850 | *bpeT* | 2.139 | 2.87E-06 | K18900 | hypothetical protein |
| APPSER1_RS10620 | *nhaA* | 2.120 | 4.15E-06 | K03313 | Na-/H- antiporter NhaA |
| APPSER1_RS03085 | *-* | 2.109 | 7.26E-07 | - | ABC transporter ATP-binding protein |
| APPSER1_RS09940 | *rpe* | 2.097 | 7.29E-06 | K01783 | ribulose-phosphate 3-epimerase |
| APPSER1_RS03865 | *cynT* | 2.084 | 7.06E-06 | K01673 | carbonate dehydratase |
| APPSER1_RS10945 | *fhuD* | 2.066 | 5.43E-06 | K23227 | iron-siderophore ABC transporter substrate-binding protein |
| APPSER1_RS10365 | *-* | 2.046 | 2.87E-06 | K07090 | sulfite exporter TauE/SafE family protein |
| APPSER1_RS06050 | *purT* | 2.008 | 1.03E-05 | K08289 | formate-dependent phosphoribosylglycinamide formyltransferase |
| APPSER1_RS05920 | *-* | 2.003 | 4.55E-06 | - | purine permease |
| APPSER1_RS09140 | *gntR* | 1.960 | 4.27E-06 | K06145 | gluconate operon transcriptional repressor GntR |
| APPSER1_RS10780 | *corA* | 1.952 | 1.03E-05 | K03284 | magnesium/cobalt transporter CorA |
| APPSER1_RS06810 | *-* | 1.934 | 2.95E-05 | K14445 | DASS family sodium-coupled anion symporter |
| APPSER1_RS04320 | *ycaO* | 1.930 | 2.72E-06 | K09136 | 30S ribosomal protein S12 methylthiotransferase accessory protein YcaO |
| APPSER1_RS07685 | *fbp* | 1.927 | 1.28E-05 | K03841 | class 1 fructose-bisphosphatase |
| APPSER1_RS10970 | *hisG* | 1.915 | 1.31E-05 | K00765 | ATP phosphoribosyltransferase |
| APPSER1_RS10310 | *ppdC* | 1.891 | 5.05E-06 | K02681 | DUF5374 domain-containing protein |
| APPSER1_RS07580 | *plsX* | 1.888 | 4.83E-06 | K03621 | phosphate acyltransferase PlsX |
| APPSER1_RS10595 | *cpdA* | 1.884 | 1.12E-05 | K03651 | 3'%2C5'-cyclic-AMP phosphodiesterase |
| APPSER1_RS10645 | *-* | 1.868 | 0.0027 | - | - |
| APPSER1_RS10090 | *cysJ* | 1.865 | 4.07E-05 | K00380 | assimilatory sulfite reductase (NADPH) flavoprotein subunit |
| APPSER1_RS10095 | *cysN* | 1.864 | 7.24E-05 | K00956 | GTP-binding protein |
| APPSER1_RS09605 | *nagA* | 1.856 | 2.62E-05 | K01443 | N-acetylglucosamine-6-phosphate deacetylase |
| APPSER1_RS04525 | *ddpD* | 1.843 | 2.79E-05 | K02031 | dipeptide/oligopeptide/nickel ABC transporter permease/ATP-binding protein |
| APPSER1_RS03300 | *-* | 1.828 | 1.25E-05 | K06956 | L-cystine transporter |
| APPSER1_RS03080 | *-* | 1.824 | 6.01E-05 | - | - |
| APPSER1_RS10940 | *fhuC* | 1.821 | 3.89E-05 | K10829 | ATP-binding cassette domain-containing protein |
| APPSER1_RS09130 | *-* | 1.806 | 7.33E-06 | K03299 | GntP family permease |
| APPSER1_RS02395 | *lctP* | 1.793 | 2.60E-05 | K03303 | L-lactate permease |
| APPSER1_RS02905 | *-* | 1.779 | 8.20E-06 | - | ABC transporter ATP-binding protein |
| APPSER1_RS11165 | *-* | 1.757 | 1.28E-05 | - | - |
| APPSER1_RS05190 | *ogt* | 1.754 | 4.41E-05 | K00567 | methylated-DNA--[protein]-cysteine S-methyltransferase |
| APPSER1_RS08435 | *-* | 1.736 | 0.00055 | - | - |
| APPSER1_RS04775 | *-* | 1.721 | 1.93E-05 | - | OFA family MFS transporter |
| APPSER1_RS05255 | *-* | 1.700 | 1.21E-05 | - | IS1595 family transposase |
| APPSER1_RS03130 | *tadA* | 1.698 | 4.49E-06 | K11991 | tRNA adenosine(34) deaminase TadA |
| APPSER1_RS10315 | *-* | 1.691 | 6.53E-06 | - | DUF2572 family protein |
| APPSER1_RS04080 | *sstT* | 1.687 | 2.01E-05 | K07862 | serine/threonine transporter SstT |
| APPSER1_RS10565 | *-* | 1.677 | 2.83E-05 | - | membrane protein |
| APPSER1_RS02890 | *-* | 1.674 | 1.21E-05 | - | DUF1007 family protein |
| APPSER1_RS08145 | *mnmE* | 1.669 | 3.09E-05 | K03650 | tRNA uridine-5-carboxymethylaminomethyl(34) synthesis GTPase MnmE |
| APPSER1_RS02390 | *lldE* | 1.658 | 4.50E-05 | K18928 | (Fe-S)-binding protein |
| APPSER1_RS06715 | *malM* | 1.652 | 6.40E-05 | K05775 | maltose operon protein MalM |
| APPSER1_RS03135 | *deaD* | 1.648 | 4.22E-05 | K05592 | DEAD/DEAH box helicase |
| APPSER1_RS07040 | *mlaE* | 1.640 | 4.99E-06 | K02066 | lipid asymmetry maintenance ABC transporter permease subunit MlaE |
| APPSER1_RS09375 | *ulaA* | 1.635 | 1.31E-05 | K03475 | PTS ascorbate transporter subunit IIC |
| APPSER1_RS10975 | *hisD* | 1.632 | 1.93E-05 | K00013 | histidinol dehydrogenase |
| APPSER1_RS09230 | *fucK* | 1.616 | 3.69E-05 | K00879 | L-fuculokinase |
| APPSER1_RS10020 | *glpT* | 1.612 | 1.31E-05 | K02445 | MFS transporter |
| APPSER1_RS04630 | *pepE* | 1.604 | 7.44E-05 | K05995 | dipeptidase PepE |
| APPSER1_RS02900 | *-* | 1.571 | 6.36E-06 | K03310 | alanine:cation symporter family protein |
| APPSER1_RS03765 | *-* | 1.563 | 4.33E-05 | - | MurR/RpiR family transcriptional regulator |
| APPSER1_RS10360 | *lgt* | 1.561 | 5.05E-06 | K13292 | prolipoprotein diacylglyceryl transferase |
| APPSER1_RS05985 | *-* | 1.556 | 3.69E-05 | - | DUF1294 domain-containing protein |
| APPSER1_RS08655 | *dlgD* | 1.549 | 1.54E-05 | K08092 | 3-dehydro-L-gulonate 2-dehydrogenase |
| APPSER1_RS02925 | *thiF* | 1.545 | 1.11E-05 | K03148 | HesA/MoeB/ThiF family protein |
| APPSER1_RS02260 | *rnhA* | 1.528 | 4.22E-05 | K03469 | ribonuclease HI |
| APPSER1_RS10085 | *cysI* | 1.525 | 0.00017 | K00381 | assimilatory sulfite reductase (NADPH) hemoprotein subunit |
| APPSER1_RS09120 | *manY* | 1.520 | 1.62E-05 | K02795 | PTS mannose/fructose/sorbose transporter subunit IIC |
| APPSER1_RS07490 | *ccmA* | 1.519 | 1.31E-05 | K02193 | cytochrome c biogenesis heme-transporting ATPase CcmA |
| APPSER1_RS09795 | *-* | 1.519 | 0.00379 | K02013 | ABC transporter ATP-binding protein |
| APPSER1_RS03535 | *purE* | 1.517 | 5.45E-05 | K01588 | 5-(carboxyamino)imidazole ribonucleotide mutase |
| APPSER1_RS09380 | *ulaB* | 1.516 | 1.29E-05 | K02822 | PTS sugar transporter subunit IIB |
| APPSER1_RS06910 | *rlmN* | 1.495 | 2.42E-05 | K06941 | bifunctional tRNA (adenosine(37)-C2)-methyltransferase TrmG/ribosomal RNA large subunit methyltransferase RlmN |
| APPSER1_RS09265 | *-* | 1.489 | 5.14E-05 | K02029 | ABC transporter permease subunit |
| APPSER1_RS02005 | *-* | 1.488 | 2.29E-05 | K02440 | aquaporin |
| APPSER1_RS05185 | *fadL* | 1.475 | 8.88E-05 | K06076 | outer membrane protein transport protein |
| APPSER1_RS09260 | *-* | 1.452 | 8.88E-05 | K02029 | amino acid ABC transporter permease |
| APPSER1_RS04795 | *truA* | 1.421 | 3.68E-05 | K06173 | tRNA pseudouridine(38-40) synthase TruA |
| APPSER1_RS05465 | *galK* | 1.410 | 2.62E-05 | K00849 | galactokinase |
| APPSER1_RS10560 | *-* | 1.399 | 4.95E-05 | - | YjiG family protein |
| APPSER1_RS05460 | *galT* | 1.385 | 8.09E-05 | K00965 | galactose-1-phosphate uridylyltransferase |
| APPSER1_RS06815 | *parE* | 1.380 | 4.24E-05 | K02622 | DNA topoisomerase IV subunit B |
| APPSER1_RS02910 | *-* | 1.359 | 0.00012 | K15600 | - |
| APPSER1_RS09535 | *-* | 1.339 | 0.00295 | - | - |
| APPSER1_RS03540 | *ansA* | 1.335 | 5.29E-05 | K01424 | asparaginase |
| APPSER1_RS07485 | *ccmB* | 1.335 | 2.70E-05 | K02194 | heme exporter protein CcmB |
| APPSER1_RS09090 | *gidA* | 1.331 | 4.45E-05 | K03495 | tRNA uridine-5-carboxymethylaminomethyl(34) synthesis enzyme MnmG |
| APPSER1_RS09790 | *-* | 1.330 | 0.00056 | K02015 | iron ABC transporter permease |
| APPSER1_RS07480 | *ccmC* | 1.324 | 3.37E-05 | K02195 | heme ABC transporter permease |
| APPSER1_RS09500 | *-* | 1.319 | 1.54E-05 | - | - |
| APPSER1_RS02930 | *thiG* | 1.317 | 3.23E-05 | K03149 | thiazole synthase |
| APPSER1_RS07495 | *hda* | 1.313 | 8.05E-05 | K10763 | DnaA regulatory inactivator Hda |
| APPSER1_RS09365 | *smf* | 1.310 | 3.38E-05 | K04096 | DNA-processing protein DprA |
| APPSER1_RS04440 | *lysE* | 1.306 | 3.69E-05 | K06895 | LysE/ArgO family amino acid transporter |
| APPSER1_RS06585 | *frmA* | 1.302 | 0.00011 | K00121 | S-(hydroxymethyl)glutathione dehydrogenase/class III alcohol dehydrogenase |
| APPSER1_RS02215 | *uppS* | 1.301 | 5.52E-05 | K00806 | polyprenyl diphosphate synthase |
| APPSER1_RS07990 | *-* | 1.300 | 7.23E-05 | - | - |
| APPSER1_RS09235 | *fucU* | 1.297 | 0.00015 | K02431 | L-fucose mutarotase |
| APPSER1_RS07585 | *rpmF* | 1.296 | 0.00203 | K02911 | 50S ribosomal protein L32 |
| APPSER1_RS09215 | *purL* | 1.294 | 0.00013 | K01952 | phosphoribosylformylglycinamidine synthase |
| APPSER1_RS02025 | *glpQ* | 1.263 | 4.48E-05 | K01126 | glycerophosphodiester phosphodiesterase |
| APPSER1_RS05990 | *purM* | 1.261 | 4.22E-05 | K01933 | phosphoribosylformylglycinamidine cyclo-ligase |
| APPSER1_RS00030 | *-* | 1.257 | 8.65E-05 | - | porin |
| APPSER1_RS03850 | *tgt* | 1.256 | 7.50E-05 | K00773 | tRNA guanosine(34) transglycosylase Tgt |
| APPSER1_RS06025 | *pepA* | 1.255 | 0.00068 | K01255 | leucyl aminopeptidase |
| APPSER1_RS00565 | *putP* | 1.252 | 0.00019 | K11928 | sodium/proline symporter PutP |
| APPSER1_RS09925 | *-* | 1.252 | 0.02506 | - | - |
| APPSER1_RS02405 | *tet35* | 1.251 | 2.29E-05 | K18218 | Na-/H- antiporter NhaC family protein |
| APPSER1_RS01855 | *glgB* | 1.247 | 0.00011 | K00700 | 1%2C4-alpha-glucan branching protein GlgB |
| APPSER1_RS03770 | *-* | 1.243 | 0.00011 | - | YhcH/YjgK/YiaL family protein |
| APPSER1_RS09115 | *manZ* | 1.230 | 3.69E-05 | K02796 | PTS mannose transporter subunit IID |
| APPSER1_RS10190 | *-* | 1.229 | 0.00013 | - | LysR family transcriptional regulator |
| APPSER1_RS09135 | *idnK* | 1.218 | 4.33E-05 | K00851 | gluconokinase |
| APPSER1_RS05570 | *-* | 1.216 | 0.0001 | K03317 | NupC/NupG family nucleoside CNT transporter |
| APPSER1_RS07575 | *fabH* | 1.215 | 5.52E-05 | K00648 | ketoacyl-ACP synthase III |
| APPSER1_RS03660 | *-* | 1.210 | 9.86E-05 | - | DUF423 domain-containing protein |
| APPSER1_RS00905 | *thiB* | 1.197 | 0.00014 | K02064 | thiamine ABC transporter substrate binding subunit |
| APPSER1_RS05435 | *katE* | 1.196 | 0.00011 | K03781 | catalase |
| APPSER1_RS08545 | *wecA* | 1.194 | 4.22E-05 | K02851 | UDP-N-acetylglucosamine--undecaprenyl-phosphate N-acetylglucosaminephosphotransferase |
| APPSER1_RS02895 | *rcnA* | 1.192 | 8.75E-05 | K08970 | nickel/cobalt transporter |
| APPSER1_RS10585 | *-* | 1.183 | 0.00015 | K08998 | membrane protein insertion efficiency factor YidD |
| APPSER1_RS07475 | *ccmD* | 1.182 | 0.00013 | K02196 | heme exporter protein CcmD |
| APPSER1_RS08975 | *rimO* | 1.180 | 5.70E-05 | K14441 | 30S ribosomal protein S12 methylthiotransferase RimO |
| APPSER1_RS07470 | *ccmE* | 1.179 | 4.48E-05 | K02197 | cytochrome c maturation protein CcmE |
| APPSER1_RS05575 | *-* | 1.173 | 0.00011 | K07149 | membrane protein |
| APPSER1_RS06750 | *-* | 1.167 | 0.00011 | - | carbon starvation protein A |
| APPSER1_RS10720 | *radC* | 1.143 | 8.88E-05 | K03630 | DNA repair protein RadC |
| APPSER1_RS02590 | *maeB* | 1.140 | 8.88E-05 | K00029 | malic enzyme |
| APPSER1_RS02385 | *lldF* | 1.137 | 0.00015 | K18929 | LutB/LldF family L-lactate oxidation iron-sulfur protein |
| APPSER1_RS00945 | *rnt* | 1.134 | 6.29E-05 | K03683 | ribonuclease T |
| APPSER1_RS00045 | *mraZ* | 1.134 | 0.00013 | K03925 | division/cell wall cluster transcriptional repressor MraZ |
| APPSER1_RS00720 | *-* | 1.126 | 4.48E-05 | - | DUF2301 domain-containing membrane protein |
| APPSER1_RS00440 | *dhaK* | 1.125 | 0.00012 | K05878 | dihydroxyacetone kinase subunit DhaK |
| APPSER1_RS02440 | *znuC* | 1.121 | 4.33E-05 | K09817 | zinc ABC transporter ATP-binding protein ZnuC |
| APPSER1_RS05415 | *-* | 1.116 | 0.00054 |  | glycosyltransferase |
| APPSER1_RS02265 | *cvpA* | 1.115 | 6.49E-05 | K03558 | CvpA family protein |
| APPSER1_RS08955 | *-* | 1.115 | 0.00034 | - | DeoR family transcriptional regulator |
| APPSER1_RS10395 | *fdhC* | 1.100 | 0.00016 | K21993 | formate/nitrite transporter family protein |
| APPSER1_RS09370 | *-* | 1.099 | 4.16E-05 | - | oligopeptide transporter%2C OPT family |
| APPSER1_RS05335 | *-* | 1.095 | 0.00012 | K07085 | putative transporter |
| APPSER1_RS05640 | *-* | 1.089 | 0.00014 | - | polysaccharide pyruvyl transferase family protein |
| APPSER1_RS03435 | *nusA* | 1.081 | 0.00033 | K02600 | transcription termination factor NusA |
| APPSER1_RS06805 | *-* | 1.073 | 0.00011 | - | SLC13 family permease |
| APPSER1_RS04335 | *cmoB* | 1.073 | 0.00012 | K15257 | tRNA 5-methoxyuridine(34)/uridine 5-oxyacetic acid(34) synthase CmoB |
| APPSER1_RS09085 | *gidB* | 1.070 | 0.00037 | K03501 | 16S rRNA (guanine(527)-N(7))-methyltransferase RsmG |
| APPSER1_RS10625 | *-* | 1.067 | 0.00032 | - | ricin-type beta-trefoil lectin domain protein |
| APPSER1_RS03425 | *-* | 1.051 | 0.01525 | - | - |
| APPSER1_RS03250 | *tyrP* | 1.048 | 0.00117 | K03834 | aromatic amino acid transporter |
| APPSER1_RS05815 | *secD* | 1.047 | 0.00012 | K03072 | protein translocase subunit SecD |
| APPSER1_RS04520 | *-* | 1.041 | 0.00054 | K02033 | ABC transporter permease |
| APPSER1_RS07150 | *nudE* | 1.040 | 0.00017 | K08312 | ADP compounds hydrolase NudE |
| APPSER1_RS10980 | *hisC* | 1.033 | 0.00093 | K00817 | histidinol-phosphate transaminase |
| APPSER1_RS05810 | *yajC* | 1.032 | 0.00029 | K03210 | preprotein translocase subunit YajC |
| APPSER1_RS10985 | *rayT* | 1.031 | 0.00038 | K07491 | IS200/IS605 family transposase |
| APPSER1_RS06035 | *lptF* | 1.029 | 7.23E-05 | K07091 | LPS export ABC transporter permease LptF |
| APPSER1_RS03235 | *guaB* | 1.021 | 0.0003 | K00088 | IMP dehydrogenase |
| APPSER1_RS06555 | *marC* | 1.016 | 0.00069 | K05595 | MarC family protein |
| APPSER1_RS01835 | *abiQ* | 1.015 | 0.01529 | K19167 | hypothetical protein |
| APPSER1_RS09785 | *-* | 1.009 | 0.00127 | - | ABC transporter substrate-binding protein |
| APPSER1_RS06740 | *malG* | 1.002 | 0.00117 | K10110 | maltose ABC transporter permease MalG |
| APPSER1_RS09680 | *rplX* | -1.002 | 0.04477 | K02895 | 50S ribosomal protein L24 |
| APPSER1_RS00530 | *nrfB* | -1.010 | 0.00029 | K04013 | cytochrome c nitrite reductase pentaheme subunit |
| APPSER1_RS00525 | *nrfA* | -1.012 | 0.0001 | K03385 | ammonia-forming nitrite reductase cytochrome c552 subunit |
| APPSER1_RS09180 | *dmsB* | -1.020 | 0.00017 | K07307 | dimethylsulfoxide reductase subunit B |
| APPSER1_RS04825 | *-* | -1.043 | 0.00203 | - | DNA-binding protein |
| APPSER1_RS04340 | *-* | -1.053 | 0.00971 | - | hypothetical protein |
| APPSER1_RS04830 | *-* | -1.099 | 0.00075 | - | regulatory protein GemA |
| APPSER1_RS09185 | *dmsC* | -1.137 | 0.0001 | K07308 | dimethyl sulfoxide reductase anchor subunit |
| APPSER1_RS09190 | *dmsD* | -1.323 | 0.00011 | K23349 | Tat proofreading chaperone DmsD |
| APPSER1_RS07915 | *napA* | -1.324 | 6.76E-05 | K02567 | nitrate reductase catalytic subunit NapA |
| APPSER1_RS07240 | *hyaA* | -1.532 | 0.00013 | K06282 | hydrogenase 2 small subunit |
| APPSER1_RS09175 | *dmsA* | -1.542 | 1.73E-05 | K07306 | molybdopterin-dependent oxidoreductase |
| APPSER1_RS03675 | *torY* | -1.679 | 5.45E-05 | K07821 | NapC/NirT family cytochrome c |
| APPSER1_RS07905 | *napF* | -1.884 | 6.66E-06 | K02572 | ferredoxin-type protein NapF |
| APPSER1_RS07910 | *napD* | -2.155 | 2.42E-05 | K02570 | chaperone NapD |
